# The evolving SARS-CoV-2 epidemic in Africa: Insights from rapidly expanding genomic surveillance

**DOI:** 10.1126/science.abq5358

**Published:** 2022-09-15

**Authors:** Houriiyah Tegally, James E. San, Matthew Cotten, Monika Moir, Bryan Tegomoh, Gerald Mboowa, Darren P. Martin, Cheryl Baxter, Arnold W. Lambisia, Amadou Diallo, Daniel G. Amoako, Moussa M. Diagne, Abay Sisay, Abdel-Rahman N. Zekri, Abdou Salam Gueye, Abdoul K. Sangare, Abdoul-Salam Ouedraogo, Abdourahmane Sow, Abdualmoniem O. Musa, Abdul K. Sesay, Abe G. Abias, Adam I. Elzagheid, Adamou Lagare, Adedotun-Sulaiman Kemi, Aden Elmi Abar, Adeniji A. Johnson, Adeola Fowotade, Adeyemi O. Oluwapelumi, Adrienne A. Amuri, Agnes Juru, Ahmed Kandeil, Ahmed Mostafa, Ahmed Rebai, Ahmed Sayed, Akano Kazeem, Aladje Balde, Alan Christoffels, Alexander J. Trotter, Allan Campbell, Alpha K. Keita, Amadou Kone, Amal Bouzid, Amal Souissi, Ambrose Agweyu, Amel Naguib, Ana V. Gutierrez, Anatole Nkeshimana, Andrew J. Page, Anges Yadouleton, Anika Vinze, Anise N. Happi, Anissa Chouikha, Arash Iranzadeh, Arisha Maharaj, Armel L. Batchi-Bouyou, Arshad Ismail, Augustina A. Sylverken, Augustine Goba, Ayoade Femi, Ayotunde E. Sijuwola, Baba Marycelin, Babatunde L. Salako, Bamidele S. Oderinde, Bankole Bolajoko, Bassirou Diarra, Belinda L. Herring, Benjamin Tsofa, Bernard Lekana-Douki, Bernard Mvula, Berthe-Marie Njanpop-Lafourcade, Blessing T. Marondera, Bouh Abdi Khaireh, Bourema Kouriba, Bright Adu, Brigitte Pool, Bronwyn McInnis, Cara Brook, Carolyn Williamson, Cassien Nduwimana, Catherine Anscombe, Catherine B. Pratt, Cathrine Scheepers, Chantal G. Akoua-Koffi, Charles N. Agoti, Chastel M. Mapanguy, Cheikh Loucoubar, Chika K. Onwuamah, Chikwe Ihekweazu, Christian N. Malaka, Christophe Peyrefitte, Chukwa Grace, Chukwuma E. Omoruyi, Clotaire D. Rafaï, Collins M. Morang’a, Cyril Erameh, Daniel B. Lule, Daniel J. Bridges, Daniel Mukadi-Bamuleka, Danny Park, David A. Rasmussen, David Baker, David J. Nokes, Deogratius Ssemwanga, Derek Tshiabuila, Dominic S. Y. Amuzu, Dominique Goedhals, Donald S. Grant, Donwilliams O. Omuoyo, Dorcas Maruapula, Dorcas W. Wanjohi, Ebenezer Foster-Nyarko, Eddy K. Lusamaki, Edgar Simulundu, Edidah M. Ong’era, Edith N. Ngabana, Edward O. Abworo, Edward Otieno, Edwin Shumba, Edwine Barasa, El Bara Ahmed, Elhadi A. Ahmed, Emmanuel Lokilo, Enatha Mukantwari, Eromon Philomena, Essia Belarbi, Etienne Simon-Loriere, Etilé A. Anoh, Eusebio Manuel, Fabian Leendertz, Fahn M. Taweh, Fares Wasfi, Fatma Abdelmoula, Faustinos T. Takawira, Fawzi Derrar, Fehintola V. Ajogbasile, Florette Treurnicht, Folarin Onikepe, Francine Ntoumi, Francisca M. Muyembe, Frank E. Z. Ragomzingba, Fred A. Dratibi, Fred-Akintunwa Iyanu, Gabriel K. Mbunsu, Gaetan Thilliez, Gemma L. Kay, George O. Akpede, Gert U. van Zyl, Gordon A. Awandare, Grace S. Kpeli, Grit Schubert, Gugu P. Maphalala, Hafaliana C. Ranaivoson, Hannah E. Omunakwe, Harris Onywera, Haruka Abe, Hela Karray, Hellen Nansumba, Henda Triki, Herve Albéric Adje Kadjo, Hesham Elgahzaly, Hlanai Gumbo, Hota Mathieu, Hugo Kavunga-Membo, Ibtihel Smeti, Idowu B. Olawoye, Ifedayo M. O. Adetifa, Ikponmwosa Odia, Ilhem Boutiba Ben Boubaker, Iluoreh Ahmed Mohammad, Isaac Ssewanyana, Isatta Wurie, Iyaloo S. Konstantinus, Jacqueline Wemboo Afiwa Halatoko, James Ayei, Janaki Sonoo, Jean-Claude C. Makangara, Jean-Jacques M. Tamfum, Jean-Michel Heraud, Jeffrey G. Shaffer, Jennifer Giandhari, Jennifer Musyoki, Jerome Nkurunziza, Jessica N. Uwanibe, Jinal N. Bhiman, Jiro Yasuda, Joana Morais, Jocelyn Kiconco, John D. Sandi, John Huddleston, John K. Odoom, John M. Morobe, John O. Gyapong, John T. Kayiwa, Johnson C. Okolie, Joicymara S. Xavier, Jones Gyamfi, Joseph F. Wamala, Joseph H. K. Bonney, Joseph Nyandwi, Josie Everatt, Joweria Nakaseegu, Joyce M. Ngoi, Joyce Namulondo, Judith U. Oguzie, Julia C. Andeko, Julius J. Lutwama, Juma J. H. Mogga, Justin O’Grady, Katherine J. Siddle, Kathleen Victoir, Kayode T. Adeyemi, Kefentse A. Tumedi, Kevin S. Carvalho, Khadija Said Mohammed, Koussay Dellagi, Kunda G. Musonda, Kwabena O. Duedu, Lamia Fki-Berrajah, Lavanya Singh, Lenora M. Kepler, Leon Biscornet, Leonardo de Oliveira Martins, Lucious Chabuka, Luicer Olubayo, Lul Deng Ojok, Lul Lojok Deng, Lynette I. Ochola-Oyier, Lynn Tyers, Madisa Mine, Magalutcheemee Ramuth, Maha Mastouri, Mahmoud ElHefnawi, Maimouna Mbanne, Maitshwarelo I. Matsheka, Malebogo Kebabonye, Mamadou Diop, Mambu Momoh, Maria da Luz Lima Mendonça, Marietjie Venter, Marietou F. Paye, Martin Faye, Martin M. Nyaga, Mathabo Mareka, Matoke-Muhia Damaris, Maureen W. Mburu, Maximillian G. Mpina, Michael Owusu, Michael R. Wiley, Mirabeau Y. Tatfeng, Mitoha Ondo’o Ayekaba, Mohamed Abouelhoda, Mohamed Amine Beloufa, Mohamed G. Seadawy, Mohamed K. Khalifa, Mooko Marethabile Matobo, Mouhamed Kane, Mounerou Salou, Mphaphi B. Mbulawa, Mulenga Mwenda, Mushal Allam, My V. T. Phan, Nabil Abid, Nadine Rujeni, Nadir Abuzaid, Nalia Ismael, Nancy Elguindy, Ndeye Marieme Top, Ndongo Dia, Nédio Mabunda, Nei-yuan Hsiao, Nelson Boricó Silochi, Ngiambudulu M. Francisco, Ngonda Saasa, Nicholas Bbosa, Nickson Murunga, Nicksy Gumede, Nicole Wolter, Nikita Sitharam, Nnaemeka Ndodo, Nnennaya A. Ajayi, Noël Tordo, Nokuzola Mbhele, Norosoa H. Razanajatovo, Nosamiefan Iguosadolo, Nwando Mba, Ojide C. Kingsley, Okogbenin Sylvanus, Oladiji Femi, Olubusuyi M. Adewumi, Olumade Testimony, Olusola A. Ogunsanya, Oluwatosin Fakayode, Onwe E. Ogah, Ope-Ewe Oludayo, Ousmane Faye, Pamela Smith-Lawrence, Pascale Ondoa, Patrice Combe, Patricia Nabisubi, Patrick Semanda, Paul E. Oluniyi, Paulo Arnaldo, Peter Kojo Quashie, Peter O. Okokhere, Philip Bejon, Philippe Dussart, Phillip A. Bester, Placide K. Mbala, Pontiano Kaleebu, Priscilla Abechi, Rabeh El-Shesheny, Rageema Joseph, Ramy Karam Aziz, René G. Essomba, Reuben Ayivor-Djanie, Richard Njouom, Richard O. Phillips, Richmond Gorman, Robert A. Kingsley, Rosa Maria D. E. S. A. Neto Rodrigues, Rosemary A. Audu, Rosina A. A. Carr, Saba Gargouri, Saber Masmoudi, Sacha Bootsma, Safietou Sankhe, Sahra Isse Mohamed, Saibu Femi, Salma Mhalla, Salome Hosch, Samar Kamal Kassim, Samar Metha, Sameh Trabelsi, Sara Hassan Agwa, Sarah Wambui Mwangi, Seydou Doumbia, Sheila Makiala-Mandanda, Sherihane Aryeetey, Shymaa S. Ahmed, Side Mohamed Ahmed, Siham Elhamoumi, Sikhulile Moyo, Silvia Lutucuta, Simani Gaseitsiwe, Simbirie Jalloh, Soa Fy Andriamandimby, Sobajo Oguntope, Solène Grayo, Sonia Lekana-Douki, Sophie Prosolek, Soumeya Ouangraoua, Stephanie van Wyk, Stephen F. Schaffner, Stephen Kanyerezi, Steve Ahuka-Mundeke, Steven Rudder, Sureshnee Pillay, Susan Nabadda, Sylvie Behillil, Sylvie L. Budiaki, Sylvie van der Werf, Tapfumanei Mashe, Thabo Mohale, Thanh Le-Viet, Thirumalaisamy P. Velavan, Tobias Schindler, Tongai G. Maponga, Trevor Bedford, Ugochukwu J. Anyaneji, Ugwu Chinedu, Upasana Ramphal, Uwem E. George, Vincent Enouf, Vishvanath Nene, Vivianne Gorova, Wael H. Roshdy, Wasim Abdul Karim, William K. Ampofo, Wolfgang Preiser, Wonderful T. Choga, Yahaya Ali Ahmed, Yajna Ramphal, Yaw Bediako, Yeshnee Naidoo, Yvan Butera, Zaydah R. de Laurent, Africa Pathogen Genomics Initiative (Africa PGI), Ahmed E. O. Ouma, Anne von Gottberg, George Githinji, Matshidiso Moeti, Oyewale Tomori, Pardis C. Sabeti, Amadou A. Sall, Samuel O. Oyola, Yenew K. Tebeje, Sofonias K. Tessema, Tulio de Oliveira, Christian Happi, Richard Lessells, John Nkengasong, Eduan Wilkinson

**Affiliations:** ^1^ Centre for Epidemic Response and Innovation (CERI), School of Data Science and Computational Thinking, Stellenbosch University, Stellenbosch, South Africa.; ^2^ KwaZulu-Natal Research Innovation and Sequencing Platform (KRISP), Nelson R Mandela School of Medicine, University of KwaZulu-Natal, Durban, South Africa.; ^3^ MRC/UVRI and LSHTM Uganda Research Unit, Entebbe, Uganda.; ^4^ MRC-University of Glasgow Centre for Virus Research, Glasgow, UK.; ^5^ The Biotechnology Centre of the University of Yaoundé I, Yaoundé, Cameroon.; ^6^ CDC Foundation, Atlanta, Georgia, Nebraska Department of Health and Human Services, Lincoln, NE, USA.; ^7^ Institute of Pathogen Genomics, Africa Centres for Disease Control and Prevention (Africa CDC), Addis Ababa, Ethiopia.; ^8^ Institute of Infectious Diseases and Molecular Medicine, Department of Integrative Biomedical Sciences, Computational Biology Division, University of Cape Town, Cape Town, South Africa.; ^9^ Division of Medical Virology, Wellcome Centre for Infectious Diseases in Africa, Institute of Infectious Disease and Molecular Medicine, University of Cape Town, Cape Town, South Africa.; ^10^ Centre for the AIDS Programme of Research in South Africa (CAPRISA), Durban, South Africa.; ^11^ KEMRI-Wellcome Trust Research Programme, Kilifi, Kenya.; ^12^ Virology Department, Institut Pasteur de Dakar, Dakar, Senegal.; ^13^ National Institute for Communicable Diseases (NICD) of the National Health Laboratory Service (NHLS), Johannesburg, South Africa.; ^14^ School of Health Sciences, College of Health Sciences, University of KwaZulu-Natal, Durban, KwaZulu-Natal, South Africa.; ^15^ Department of Medical Laboratory Sciences, College of Health Sciences, Addis Ababa University, Addis Ababa, Ethiopia.; ^16^ Department of Microbial, Cellular and Molecular Biology, College of Natural and Computational Sciences, Addis Ababa University, Addis Ababa, Ethiopia.; ^17^ Cancer Biology Department, Virology and Immunology Unit, National Cancer Institute, Cairo University, Cairo, Egypt.; ^18^ World Health Organization, Africa Region, Brazzaville, Republic of the Congo.; ^19^ Centre d’Infectiologie Charles Mérieux-Mali (CICM-Mali), Bamako, Mali.; ^20^ Bacteriology and Virology Department Souro Sanou University Hospital, Bobo-Dioulasso, Burkina Faso.; ^21^ West African Health Organisation, Bobo-Dioulasso, Burkina Faso.; ^22^ Faculty of Medicine and Health Sciences, Kassala University, Kassala City, Sudan.; ^23^ Department of Microbiology, Faculty of Medical Laboratory Sciences, University of Gezira, Gezira, Sudan.; ^24^ General Administration of Laboratories and Blood Banks, Ministry of Health, Kassala State, Sudan.; ^25^ MRC Unit The Gambia at LSHTM, Fajara, Gambia.; ^26^ National Public Health Laboratory, Ministry of Health, Juba, Republic of South Sudan.; ^27^ Libyan Biotechnology Research Center, Tripoli, Libya.; ^28^ Center for Medical and Sanitary Research (CERMES), Niamey, Niger.; ^29^ The Nigerian Institute of Medical Research, Yaba, Lagos, Nigeria.; ^30^ Laboratoire de la Caisse Nationale de Sécurité Sociale, Djibouti, Republic of Djibouti.; ^31^ Department of Virology, College of Medicine, University of Ibadan, Ibadan, Nigeria.; ^32^ Infectious Disease Institute, College of Medicine, University of Ibadan, Ibadan, Nigeria.; ^33^ Medical Microbiology and Parasitology Department, College of Medicine, University of Ibadan, Ibadan, Nigeria.; ^34^ Biorepository Clinical Virology Laboratory, College of Medicine, University of Ibadan, Ibadan, Nigeria.; ^35^ Department of Medical Microbiology and Parasitology, Faculty of Basic Clinical Sciences, College of Health Sciences, University of Ilorin, Ilorin, Kwara State, Nigeria.; ^36^ The Pirbright Institute, Woking, UK.; ^37^ Pathogen Sequencing Lab, Institut National de Recherche Biomédicale (INRB), Kinshasa, the Democratic Republic of the Congo.; ^38^ Université de Kinshasa (UNIKIN), Kinshasa, the Democratic Republic of the Congo.; ^39^ National Microbiology Reference Laboratory, Harare, Zimbabwe.; ^40^ Center of Scientific Excellence for Influenza Viruses, National Research Centre (NRC), Cairo, Egypt.; ^41^ Laboratory of Molecular and Cellular Screening Processes, Centre of Biotechnology of Sfax, University of Sfax, Sfax, Tunisia.; ^42^ Genomics and Epigenomics Program, Research Department CCHE57357, Cairo, Egypt.; ^43^ African Centre of Excellence for Genomics of Infectious Diseases (ACEGID), Redeemer’s University, Ede, Osun State, Nigeria.; ^44^ Department of Biological Sciences, Faculty of Natural Sciences, Redeemer’s University, Ede, Osun State, Nigeria.; ^45^ Laboratório de Biologia Molecular Jean Piaget, Bissau, Guinea-Bissau.; ^46^ University Jean Piaget in Guinea-Bissau, Bissau, Guinea-Bissau.; ^47^ SAMRC Bioinformatics Unit, SA Bioinformatics Institute, University of the Western Cape, Cape Town, South Africa.; ^48^ Quadram Institute Bioscience, Norwich, UK.; ^49^ Central Public Health Reference Laboratories, Freetown, Sierra Leone.; ^50^ Centre de Recherche et de Formation en Infectiologie de Guinée (CERFIG), Université de Conakry, Conakry, Guinea.; ^51^ TransVIHMI, Institut de Recherche pour le Développement, Institut National de la Santé et de la Recherche Médicale (INSERM), Montpellier University, 34090, Montpellier, France.; ^52^ University Clinical Research Center (UCRC), University of Sciences, Techniques and Technology of Bamako, Bamako, Mali.; ^53^ Sharjah Institute for Medical Research, College of Medicine, University of Sharjah, Sharjah, United Arab Emirates.; ^54^ Central Public Health Laboratories (CPHL), Cairo, Egypt.; ^55^ National Institute of Public Health, Bujumbura, Burundi.; ^56^ Laboratoire des Fièvres Hémorragiques Virales du Benin, Cotonou, Benin.; ^57^ Infectious Disease and Microbiome Program, Broad Institute of Harvard and MIT, Cambridge, MA, USA.; ^58^ Laboratory of Clinical Virology, WHO Reference Laboratory for Poliomyelitis and Measles in the Eastern Mediterranean Region, Pasteur Institute of Tunis, University Tunis El Manar (UTM), Tunis 1002, Tunisia.; ^59^ Research Laboratory “Virus, Vectors and Hosts: One Health Apporach and Technological Innovation for a Better Health”, LR20IPT02, Pasteur Institute, Tunis 1002, Tunisia.; ^60^ Fondation Congolaise pour la Recherche Médicale, Brazzaville, Republic of the Congo.; ^61^ Marien Ngouabi, Brazzaville, Republic of the Congo.; ^62^ Kwame Nkrumah University of Science and Technology, Department of Theoretical and Applied Biology, Kumasi, Ghana.; ^63^ Kumasi Centre for Collaborative Research in Tropical Medicine, Kwame Nkrumah University of Science and Technology, Kumasi, Ghana.; ^64^ Viral Haemorrhagic Fever Laboratory, Kenema Government Hospital, Kenema, Sierra Leone.; ^65^ Ministry of Health and Sanitation, Freetown, Sierra Leone.; ^66^ Department of Immunology, University of Maiduguri Teaching Hospital, P.M.B. 1414, Maiduguri, Nigeria.; ^67^ Department of Medical Laboratory Science, College of Medical Sciences, University of Maiduguri, P.M.B. 1069, Maiduguri, Borno State, Nigeria.; ^68^ Centre Interdisciplinaires de Recherches Medicales de Franceville (CIRMF), Franceville, Gabon.; ^69^ Département de Parasitologie-Mycologie Université des Sciences de la Santé (USS), Libreville, Gabon.; ^70^ National HIV Reference Laboratory, Community Health Sciences Unit, Ministry of Health, Lilongwe, Malawi.; ^71^ African Society for Laboratory Medicine, Addis Ababa, Ethiopia.; ^72^ National Medical and Molecular Biology Laboratory, Ministry of Health, Djibouti, Republic of Djibouti.; ^73^ Africa CDC, Rapid Responder, Team Djibouti, Djibouti, Djibouti.; ^74^ Noguchi Memorial Institute for Medical Research, University of Ghana, Legon, Ghana.; ^75^ Seychelles Public Health Laboratory, Public Health Authority, Ministry of Health Seychelles, Victoria, Seychelles.; ^76^ Department of Ecology and Evolution, University of Chicago, Chicago, IL, USA.; ^77^ Virology Unit, Institut Pasteur de Madagascar, Antananarivo, Madagascar.; ^78^ National Health Laboratory Service (NHLS), Cape Town, South Africa.; ^79^ Malawi-Liverpool-Wellcome Trust Clinical Research Programme, Blantyre, Malawi.; ^80^ Liverpool School of Tropical Medicine, Liverpool, UK.; ^81^ University of Nebraska Medical Center (UNMC), Omaha, NE, USA.; ^82^ SAMRC Antibody Immunity Research Unit, School of Pathology, University of the Witwatersrand, Johannesburg, South Africa.; ^83^ CHU de Bouaké, Laboratoire/Unité de Diagnostic des Virus des Fièvres Hémorragiques et Virus Émergents, Bouaké, Côte d’Ivoire.; ^84^ UFR Sciences Médicales, Universite Alassane Ouattara, Bouaké, Côte d’Ivoire.; ^85^ School of Public Health, Pwani University, Kilifi, Kenya.; ^86^ Faculty of Science and Techniques, University Marien Ngouabi, Brazzaville, Republic of the Congo.; ^87^ Centre for Human Virology and Genomics, Nigerian Institute of Medical Research, Yaba, Lagos, Nigeria.; ^88^ Nigeria Centre for Disease Control and Prevention, Abuja, Nigeria.; ^89^ Laboratoire des Arbovirus, Fièvres Hémorragiques virales, Virus Emergents et Zoonoses, Institut Pasteur de Bangui, Bangui, Central African Republic.; ^90^ Le Laboratoire National de Biologie Clinique et de Santé Publique (LNBCSP), Bangui, Central African Republic.; ^91^ West African Centre for Cell Biology of Infectious Pathogens (WACCBIP), College of Basic and Applied Sciences, University of Ghana, Accra, Ghana.; ^92^ Institute of Lassa Fever Research and Control, Irrua Specialist Teaching Hospital, Irrua, Nigeria.; ^93^ PATH, Lusaka, Zambia.; ^94^ Department of Entomology and Plant Pathology, North Carolina State University, Raleigh, NC, USA.; ^95^ Bioinformatics Research Center, North Carolina State University, Raleigh, NC, USA.; ^96^ School of Life Sciences and Zeeman Institute for Systems Biology and Infectious Disease Epidemiology Research (SBIDER), University of Warwick, Coventry, UK.; ^97^ Uganda Virus Research Institute, Entebbe, Uganda.; ^98^ PathCare Vermaak, Pretoria, South Africa and Division of Virology, University of the Free State, Bloemfontein, South Africa.; ^99^ College of Medicine and Allied Health Sciences, University of Sierra Leone, Freetown, Sierra Leone.; ^100^ Botswana Harvard AIDS Institute Partnership and Botswana Harvard HIV Reference Laboratory, Gaborone, Botswana.; ^101^ Macha Research Trust, Choma, Zambia.; ^102^ International Livestock Research Institute (ILRI), Nairobi, Kenya.; ^103^ INRSP, Nouakchott, Mauritania.; ^104^ Faculté de Médecine de Nouakchott, Nouakchott, Mauritani.; ^105^ Rwanda National Reference Laboratory, Kigali, Rwanda.; ^106^ Robert Koch-Institute, Berlin, Germany.; ^107^ G5 Evolutionary Genomics of RNA Viruses, Institut Pasteur, Paris, France.; ^108^ Direcção Nacional da Saúde Pública, Ministério da Saúde, Luanda, Angola.; ^109^ National Public Health Reference Laboratory–National Public Health Institute of Liberia, Monrovia, Liberia.; ^110^ Faculty of Pharmacy of Monastir, Monastir, Tunisia.; ^111^ National Influenza Centre, Institut Pasteur d’Algérie, Algiers, Algeria.; ^112^ Department of Virology, National Health Laboratory Service (NHLS), Charlotte Maxeke Johannesburg Academic Hospital, Johannesburg, South Africa.; ^113^ School of Pathology, Faculty of Health Science, University of the Witwatersrand, Johannesburg, South Africa.; ^114^ Institute of Tropical Medicine, Universitätsklinikum Tübingen, Tübingen, Germany.; ^115^ Ministère de Santé Publique et de la Solidarité Nationale, Ndjamena, Chad.; ^116^ WHO Int Comoros, Moroni, Union of Comoros.; ^117^ World Health Organization, Africa Region, Brazzaville, Republic of the Congo.; ^118^ Division of Medical Virology, Faculty of Medicine and Health Sciences, Stellenbosch University, Tygerberg, Cape Town, South Africa.; ^119^ National Health Laboratory Service (NHLS), Tygerberg, Cape Town, South Africa.; ^120^ UHAS COVID-19 Testing and Research Centre, University of Health and Allied Sciences, Ho, Ghana.; ^121^ Department of Biomedical Sciences, University of Health and Allied Sciences, PMB 31, Ho, Ghana.; ^122^ Ministry of Health, COVID-19 Testing Laboratory, Mbabane, Kingdom of Eswatini.; ^123^ Satellite Molecular Laboratory, Rivers State University Teaching Hospital, Port Harcourt, Nigeria.; ^124^ Department of Emerging Infectious Diseases, Institute of Tropical Medicine, Nagasaki University, Nagasaki, Japan.; ^125^ CHU Habib Bourguiba, Laboratory of Microbiology, Faculty of Medicine of Sfax, University of Sfax, Sfax, Tunisia.; ^126^ Central Public Health Laboratories (CPHL), Kampala, Uganda.; ^127^ Institut Pasteur de Côte d’Ivoire, Departement des Virus Epidemiques, Abidjan, Côte d’Ivoire.; ^128^ Faculty of Medicine Ain Shams Research Institute (MASRI), Ain Shams University, Cairo, Egypt.; ^129^ Doctoral School of Technical and Environmental Sciences, Department of Biology and Human Health, N’Djamena, Chad.; ^130^ Department of Infectious Diseases Epidemiology, London School of Hygiene and Tropical Medicine, London, UK.; ^131^ Charles Nicolle Hospital, Laboratory of Microbiology, National Influenza Center, Tunis, Tunisia.; ^132^ University of Tunis El Manar, Faculty of Medicine of Tunis, Research Laboratory LR99ES09, Tunis, Tunisia.; ^133^ College of Medicine and Allied Health Science, University of Sierra Leone, Freetown, Sierra Leone.; ^134^ Namibia Institute of Pathology, Windhoek, Namibia.; ^135^ National Institute of Hygiene, Lomé, Togo.; ^136^ Virology/Molecular Biology Department, Central Health Laboratory, Victoria Hospital, Ministry of Health and Wellness, Port Louis, Mauritius.; ^137^ Department of Biostatistics and Data Science, School of Public Health and Tropical Medicine, Tulane University, New Orleans, LA, USA.; ^138^ WHO Burundi, Gitega, Burundi.; ^139^ Grupo de Investigação Microbiana e Imunológica, Instituto Nacional de Investigação em Saúde (National Institute for Health Research), Luanda, Angola.; ^140^ Departamento de Bioquímica, Faculdade de Medicina, Universidade Agostinho Neto, Luanda, Angola.; ^141^ Vaccine and Infectious Disease Division, Fred Hutchinson Cancer Center, Seattle, WA, USA.; ^142^ Universidade Federal de Minas Gerais, Belo Horizonte, Brazil.; ^143^ Institute of Agricultural Sciences, Universidade Federal dos Vales do Jequitinhonha e Mucuri, Unaí, Brazil.; ^144^ WHO South Sudan, Juba, South Sudan.; ^145^ Faculty of Medicine, University of Burundi, Bujumbura, Burundi.; ^146^ Pasteur Network, Institut Pasteur, Paris, France.; ^147^ Botswana Institute for Technology Research and Innovation, Gaborone, Botswana.; ^148^ Instituto Nacional de Saúde Pública, Praia, Cape Verde.; ^149^ Zambia National Public Health Institute, Lusaka, Zambia.; ^150^ Public Health Institute of Malawi, Lilongwe, Malawi.; ^151^ National Health Laboratory, Gaborone, Botswana.; ^152^ Laboratory of Transmissible Diseases and Biologically Active Substances (LR99ES27), Faculty of Pharmacy, University of Monastir, Monastir, Tunisia.; ^153^ Laboratory of Microbiology, University Hospital of Monastir, Monastir, Tunisia.; ^154^ Biomedical Informatics and Chemoinformatics Group, Informatics and Systems Department, National Research Centre, Cairo, Egypt.; ^155^ Ministry of Health and Wellness, Gaborone, Botswana.; ^156^ Eastern Technical University of Sierra Leone, Kenema, Sierra Leone.; ^157^ Zoonotic Arbo and Respiratory Virus Program, Centre for Viral Zoonoses, Department of Medical Virology, University of Pretoria, Pretoria, South Africa.; ^158^ Next Generation Sequencing Unit and Division of Virology, Faculty of Health Sciences, University of the Free State, Bloemfontein, South Africa.; ^159^ National Reference Laboratory Lesotho, Maseru, Lesotho.; ^160^ Centre for Biotechnology Research and Development, Kenya Medical Research Institute, Nairobi, Kenya.; ^161^ Swiss Tropical and Public Health Institute, Basel, Switzerland.; ^162^ Laboratorio de Investigaciones de Baney, Baney, Equatorial Guinea.; ^163^ Ifakara Health Insitute, Ifakara, Tanzania.; ^164^ Department of Medical Diagnostics, Kumasi Centre for Collaborative Research in Tropical Medicine, Kwame Nkrumah University of Science and Technology, Kumasi, Ghana.; ^165^ PraesensBio, Lincoln, NE, USA.; ^166^ Department of Medical Laboratory Science, Niger Delta University, Bayelsa State, Nigeria.; ^167^ Systems and Biomedical Engineering Department, Faculty of Engineering, Cairo University, Cairo, Egypt.; ^168^ King Faisal Specialist Hospital and Research Center, Riyadh, Kingdom of Saudi Arabia.; ^169^ Biological Prevention Department, Ministry of Defence, Cairo, Egypt.; ^170^ Faculty of Science, Fayoum University, Fayoum, Egypt.; ^171^ Molecular Pathology Lab, Children’s Cancer Hospital, Cairo, Egypt.; ^172^ Laboratoire Biolim FSS/Université de Lomé, Lomé, Togo.; ^173^ Department of Genetics and Genomics, College of Medicine and Health Sciences, United Arab Emirates University, Abu Dhabi, United Arab Emirates.; ^174^ High Institute of Biotechnology of Monastir, University of Monastir, Rue Taher Haddad 5000, Monastir, Tunisia.; ^175^ Rwanda National Joint Task Force COVID-19, Rwanda Biomedical Centre, Ministry of Health, Kigali, Rwanda.; ^176^ School of Health Sciences, College of Medicine and Health Sciences, University of Rwanda, Kigali, Rwanda.; ^177^ Department of Microbiology, Faculty of Medical Laboratory Sciences, Omdurman Islamic University, Sudan.; ^178^ Instituto Nacional de Saúde (INS), Marracuene, Mozambique.; ^179^ Department of Disease Control, School of Veterinary Medicine, University of Zambia, Lusaka, Zambia.; ^180^ Internal Medicine Department, Alex Ekwueme Federal University Teaching Hospital, Abakaliki, Nigeria.; ^181^ Institut Pasteur de Guinée, Conarky, Guinea.; ^182^ Virology Laboratory, Alex Ekwueme Federal University Teaching Hospital, Abakaliki, Nigeria.; ^183^ Department of Epidemiology and Community Health, Faculty of Clinical Sciences. College of Health Sciences. University of Ilorin, Ilorin, Kwara State, Nigeria.; ^184^ Department of Public Health, Ministry of Health, Ilorin, Kwara State, Nigeria.; ^185^ Alex Ekwueme Federal University Teaching Hospital, Abakaliki, Nigeria.; ^186^ Mayotte Hospital Center, Mayotte, France.; ^187^ The African Center of Excellence in Bioinformatics and Data-Intensive Sciences, The Infectious Diseases Institute, Kampala, Uganda.; ^188^ Immunology and Molecular Biology, Makerere University, Kampala, Uganda.; ^189^ Department of Medicine, Faculty of Clinical Sciences, College of Medicine, Ambrose Alli University, Ekpoma, Edo State, Nigeria.; ^190^ Division of Virology, National Health Laboratory Service and University of the Free State, Bloemfontein, South Africa.; ^191^ Infectious Hazards Preparedness, World Health Organization, Eastern Mediterranean Regional Office, Cairo, Egypt.; ^192^ Department of Microbiology and Immunology, Faculty of Pharmacy, Cairo University, Cairo, Egypt.; ^193^ Microbiology and Immunology Research Program, Children’s Cancer Hospital Egypt, Cairo, Egypt.; ^194^ National Public Health Laboratory, Ministry of Public Health of Cameroon, Yaoundé, Cameroon.; ^195^ Faculty of Medicine and Biomedical Sciences, University of Yaoundé, Yaoundé, Cameroon.; ^196^ Virology Service, Centre Pasteur of Cameroun, Yaounde, Cameroon.; ^197^ Coordenadora da rede do Diagnóstico Tuberculose/HIV/COVID-19 na Instituição - Laboratório Nacional de Referência da Tuberculose em São Tomé e Príncipe, São Tomé, São Tomé and Principe.; ^198^ Ponto focal para Melhoria da qualidade dos Laboratórios (SLIPTA) ao nível de São Tomé e Príncipe, São Tomé, São Tomé and Principe.; ^199^ National Public Health Reference Laboratory (NPHRL), Mogadishu, Somalia.; ^200^ Faculty of Medicine of Monastir, University of Monastir, Monastir, Tunisia.; ^201^ University of Basel, Basel, Switzerland.; ^202^ Clinical and Experimental Pharmacology Lab, LR16SP02, National Center of Pharmacovigilance, University of Tunis El Manar, Tunis, Tunisia.; ^203^ Harvard T.H. Chan School of Public Health, Boston, MA, USA.; ^204^ Centre MURAZ, Ouagadougou, Burkina Faso.; ^205^ National Institute of Public Health of Burkina Faso (INSP/BF), Ouagadougou, Burkina Faso.; ^206^ National Reference Center for Respiratory Viruses, Molecular Genetics of RNA Viruses, UMR 3569 CNRS, Université Paris Cité, Institut Pasteur, Paris, France.; ^207^ World Health Organization, Harare, Zimbabwe.; ^208^ Vietnamese-German Center for Medical Research, Hanoi, Vietnam.; ^209^ Howard Hughes Medical Institute, Fred Hutchinson Cancer Center, Seattle, WA, USA.; ^210^ Sub-Saharan African Network For TB/HIV Research Excellence (SANTHE), Durban, South Africa.; ^211^ World Health Organization, WHO Lesotho, Maseru, Lesotho.; ^212^ Med24 Medical Centre, Ruwa, Zimbabwe.; ^213^ Department of Virology, Noguchi Memorial Institute for Medical Research, University of Ghana, Legon, Ghana.; ^214^ Division of Human Genetics, Department of Pathology, University of Cape Town, Cape Town, South Africa.; ^215^ Yemaachi Biotech, Accra, Ghana.; ^216^ Center for Human Genetics, College of Medicine and Health Sciences, University of Rwanda, Kigali, Rwanda.; ^217^ Laboratory of Human Genetics, GIGA Research Institute, Liège, Belgium.; ^218^ Department of Biochemistry and Biotechnology, Pwani University, Kilifi, Kenya.; ^219^ Department of Global Health, University of Washington, Seattle, WA, USA.

## Abstract

Investment in SARS-CoV-2 sequencing in Africa over the past year has led to a major increase in the number of sequences generated, now exceeding 100,000 genomes, used to track the pandemic on the continent. Our results show an increase in the number of African countries able to sequence domestically, and highlight that local sequencing enables faster turnaround time and more regular routine surveillance. Despite limitations of low testing proportions, findings from this genomic surveillance study underscore the heterogeneous nature of the pandemic and shed light on the distinct dispersal dynamics of Variants of Concern, particularly Alpha, Beta, Delta, and Omicron, on the continent. Sustained investment for diagnostics and genomic surveillance in Africa is needed as the virus continues to evolve, while the continent faces many emerging and re-emerging infectious disease threats. These investments are crucial for pandemic preparedness and response and will serve the health of the continent well into the 21st century.

What originally started as a small cluster of pneumonia cases in Wuhan, China over two years ago (*1*), quickly turned into a global pandemic. Coronavirus Disease 2019 (COVID-19) is the clinical manifestation of severe acute respiratory syndrome coronavirus 2 (SARS-CoV-2) infection; and by March 2022 there had been over 437 million reported cases and over 5.9 million reported deaths (*2*). Though Africa accounts for the lowest number of reported cases and deaths thus far, with ~11.3 million reported cases and 245 000 reported deaths as of February 2022, the continent has played an important role in shaping the scientific response to the pandemic with the implementation of genomic surveillance and the identification of two of the five variants of concerns (VOCs) (*3*, *4*).

Since it emerged in 2019, SARS-CoV-2 has continued to evolve and adapt (*5*). This has led to the emergence of several viral lineages that carry mutations that confer some viral adaptive advantages that increase transmission and infection (*6*, *7*), or counter the effect of neutralizing antibodies from vaccination (*8*) or previous infections (*9*–*11*). The World Health Organization (WHO) classifies certain viral lineages as variants of concert (VOCs) or variants of interest (VOIs) based on the potential impact they may have on the pandemic, with VOCs regarded as the highest risk. To date, five VOCs have been classified by the WHO, two of which were first detected on the African continent (Beta and Omicron) (*3*, *4*, *12*), while two more (Alpha and Delta) (*12*, *13*) have spread extensively on the continent in successive waves. The remaining VOC, Gamma (*14*), originated in Brazil and had a limited influence in Africa with only four recorded sequenced cases.

For genomic surveillance to be useful for public health responses, sampling for sequencing needs to be both spatially and temporally representative. In the case of SARS-CoV-2 in Africa, this means extending the geographic coverage of sequencing capacity to capture the dynamic genomic epidemiology in as many locations as possible. In a meta-analysis of the first 10 000 SARS-CoV-2 sequences generated in 2020 from Africa (*15*) several blind spots were identified with regards to genomic surveillance on the continent. Since then, much investment has been devoted to building capacity for genomic surveillance in Africa, coordinated mostly by the Africa Centers for Disease Control (Africa CDC) and the regional office of the WHO in Africa (or WHO AFRO), but also provided by several national and international partners resulting in an additional 90 000 sequences shared over the past year (April 2021 - March 2022). This makes the sequencing effort for SARS-CoV-2 a phenomenal milestone. In comparison, only 12 000 whole genome influenza sequences (*16*) and only ~3 700 whole genome HIV sequences (*17*) from Africa have been shared publicly even though HIV has plagued the continent for decades.

Here we describe how the first 100 000 SARS-CoV-2 sequences from Africa have helped describe the pandemic on the continent, how this genomic surveillance in Africa has expanded, and how we adapted our sequencing methods to deal with an evolving virus. We also highlight the impact that genomic sequencing in Africa has had on the global public health response, particularly through the identification and early analysis of new variants. Finally, we also describe here for the first time how the Delta and Omicron variants have spread across the continent, and how their transmission dynamics were distinct from the Alpha and Beta variants that preceded them.

## Results

### Epidemic waves driven by variant dynamics and geography

**Fig. 1.**
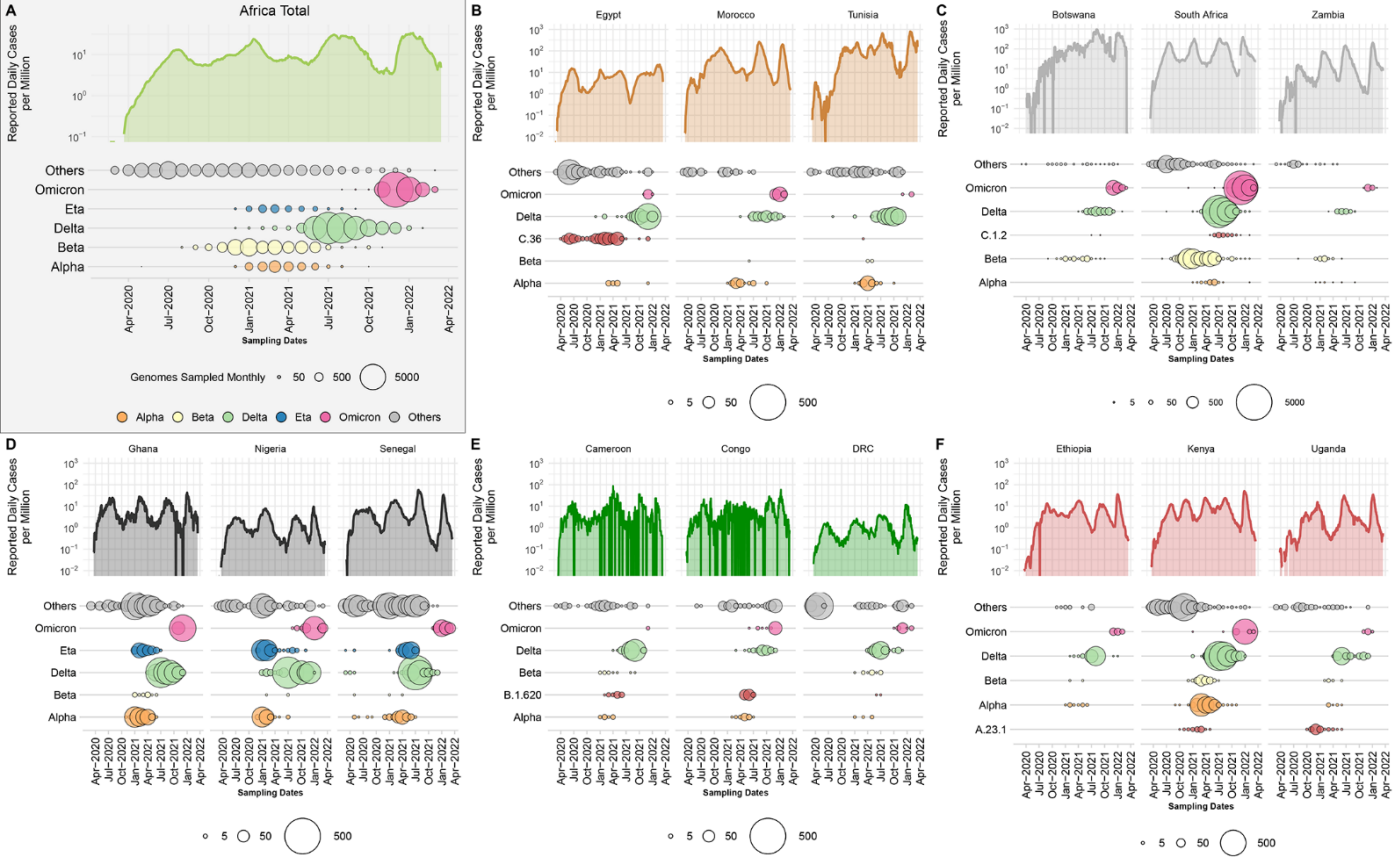
Epidemiological progression of the COVID-19 pandemic on the African continent. (**A**) Total reported new case counts per million inhabitants in Africa (Data Source: Our World in Data; OWID; log-transformed) along with the distribution of VOCs, the Eta VOI and other lineages through time (size of circles proportional to the number of genomes sampled per month for each category). (**B** to **F**) Breakdown of reported new cases per million (Data Source: Our World in Data; OWID; log-transformed) and monthly sampling of VOCs, regional variant or lineage of interest and other lineages for three selected countries for North, Southern, West, Central and East Africa respectively. For each region, a different variant or lineage of interest is shown, relevant to that region (C.36, C.1.2, Eta, B.1.620 and A.23.1, respectively).

Scaling up sequencing in Africa has provided a wealth of information on how the pandemic unfolded on the continent. The epidemic has largely been spatially heterogeneous across Africa, but most countries have experienced multiple waves of infection (*18*–*29*), with significant local and regional diversity in the first and to a lesser extent the second waves, followed by successive sweeps of the continent with Delta and Omicron (Fig. 1A). In all regions of the continent, different lineages and VOIs evolved and co-circulated with VOCs and in some cases, contributed considerably to epidemic waves.

In North Africa (Fig. 1B and fig. S1A), B.1 lineages and Alpha dominated in the first and second wave of the pandemic and were replaced by Delta and Omicron in the third and fourth waves, respectively. Interestingly, the C.36 and C.36.3 sub-lineage dominated the epidemic in Egypt (~40% of reported infections) before July 2021 when it was replaced by Delta (*30*). Similarly, in Tunisia the first and second waves were associated with the B.1.160 lineage and were replaced by Delta during the country’s third wave of infections. In southern Africa (Fig. 1C and fig. S1C), we see a similar pandemic profile with B.1 dominating the first wave, but instead of Alpha, Beta was responsible for the second wave, followed by Delta and Omicron. Another lineage that was flagged for close monitoring in the region was C.1.2, due to its mutational profile and predicted capacity for immune escape (*31*). However, the C.1.2 lineage did not cause many infections in the region as it was circulating at a time when Delta was dominant. In West Africa (Fig. 1D and fig. S1B), the B.1.525 lineage caused a large proportion of infections in the second and third waves where it shared the pandemic landscape with the Alpha variant. As with other regions on the continent, these variants were later replaced by the Delta and then Omicron VOCs in successive waves. In Central Africa (Fig. 1E and fig. S1D), the B.1.620 lineage caused most of the infections between January and June 2021 (*32*) before systematically being replaced by Delta and then Omicron. Lastly, in East Africa (Fig. 1F and fig. S1E) the A.23.1 lineage dominated the second wave of infections in Uganda (*33*) and much of East Africa. In all of these regions, minor lineages such as B.1.525, C.36 and A.23.1 were eventually replaced by VOCs that emerged in later waves.

Finally, we directly compared the official recorded cases in Africa with the ongoing SARS-CoV-2 genomic surveillance (GISAID date of access 2022-03-31) for a crude estimation of variants’ contribution to cases. We observe that Delta was responsible for an epidemic wave between May and October 2021 (Fig. 1A) and had the greatest impact on the continent with almost 34.2% of overall infections in Africa possibly attributed to it. Beta was responsible for an epidemic wave at the end of 2020 and beginning of 2021 (Fig. 1A), with 13.3% of infections overall attributed to it. Notably, Alpha, despite being predominant in other parts of the world at the beginning of 2021, had only minimal significance in Africa, accounting for just 4.3% of infections. At the time of writing, the Omicron VOC had contributed to 21.6% of overall sequenced infections. At this time the Omicron wave was still unfolding globally and in Africa with the expansion of several sub-lineages (*34*), such that its full impact is yet to be determined. However, due to increased population immunity (*35*), from SARS-CoV-2 infection and vaccination (fig. S2), the impact of Omicron on mortality has been less in comparison to the other VOCs, as can be observed by the relatively low death rate in South Africa during the Omicron wave (*36*). The findings from mapping epidemiological numbers onto genomic surveillance data are reliable as far as the proportional scaling of genomic sampling across Africa with the size and timing of epidemic waves (fig. S3; b = 0.011, SE = 0.001, *p* < 2 × 10^−16^).

This comes with the obvious caveats that testing and reporting practices have varied widely across the continent, along with genomic surveillance volumes throughout the pandemic. Countries in Africa with reported data have tested in proportions from as little as 0.1 daily tests per million population to more than 1 000 tests per million (fig. S4). Some countries have consistently tested at high proportions, for example South Africa, Botswana, Morocco and Tunisia. Incidentally, these countries have also generally reported more cases per million, providing an indication that recorded low incidence in other parts of the continent has been an underestimate due to low testing rates. However, even for these countries, epidemic numbers are certainly under represented and under detected, given that in several timeframes, test positivity rates were still on the higher end, approaching or exceeding 20% (fig. S4), and as concluded by seroprevalence surveys and estimates of true infection burdens in Africa (*37*, *38*). Findings of attributing case numbers of variants must therefore be interpreted in context of this limitation but can nevertheless provide a qualitative overview of the spatial and temporal dynamics of VOCs in relation to epidemic progression in Africa.

The African regional- (table S1) and country-specific (table S2) NextStrain builds also clearly support the changing nature of the pandemic over time. From these builds we observe a strong association of B.1-like viruses circulating on the continent during the first wave. These “ancestral” lineages were subsequently replaced by the Alpha and Beta variants which dominated the pandemic landscape during the second wave, and were later replaced by the Delta and Omicron variants during the third and fourth waves.

### Optimizing surveillance coverage in Africa

**Fig. 2.**
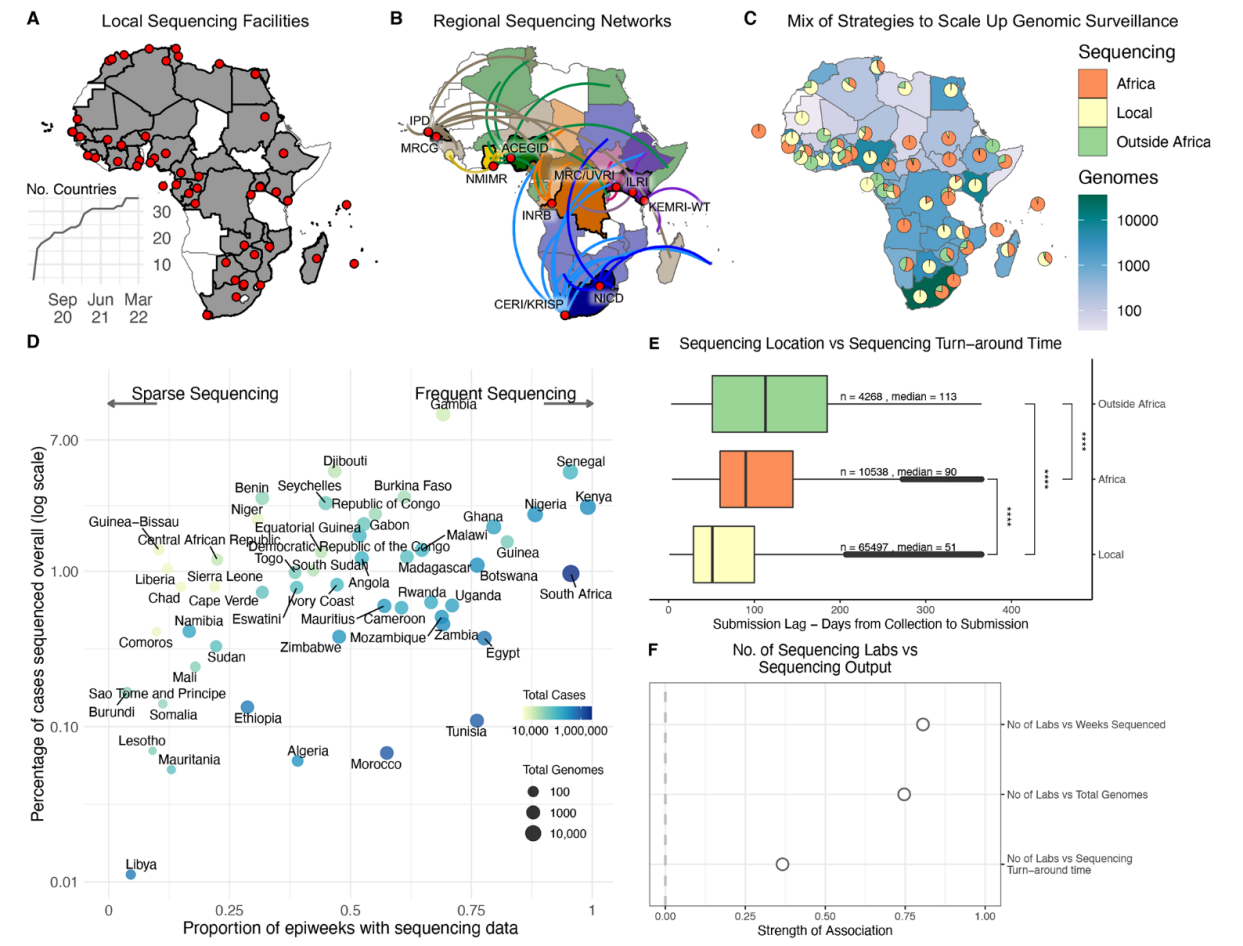
Sequencing strategies and outputs in Africa. (**A**) Geographical representation of all countries (shaded in gray) and institutions (red dots) in Africa with their own on-site sequencing facilities. The inset graph shows the number of countries in Africa able to carry out sequencing locally over time. (**B**) Key regional sequencing hubs and networks in Africa showing countries (shaded in bright colors) and institutions (red dots) that have sequenced for other countries (shaded in corresponding light colors and linking curves) on the continent. CERI: Centre for Epidemic Response and Innovation; KRISP: KwaZulu-Natal Research Innovation and Sequencing Platform; NICD: National Institute for Communicable Diseases; KEMRI-WT: Kenya Medical Research Institute - Wellcome Trust; ILRI: International Livestock Research Institute; MRC/UVRI: Medical Research Council/Uganda Virus Research Institute; INRB: Institut National de Recherche Biomédicale; ACEGID: African Centre of Excellence for Genomics of Infectious Diseases; NMIMR: Noguchi Memorial Institute for Medical Research; MRCG: Medical Research Council Unit - The Gambia; IPD: Institut Pasteur de Dakar. (**C**) Geographical representation of the total number of SARS-CoV-2 whole genomes produced over the course of the pandemic in each country, as well as the proportion of those sequences that were produced locally, regionally or abroad. (**D**) Correlation of the proportion of COVID-19 positive cases that have been sequenced and the corresponding number of epidemiological weeks since the start of the pandemic that are represented with genomes for each African country. The color of each circle represents the number of cases and its size the number of genomes. (**E**) Comparison of sequencing turn-around times (lag times from sample collection to sequence submission) for the three strategies of sequencing in Africa, showing a significant difference in the means (p-value<0.0001). The box and whisker plot denote the lower quartile, median and upper quartile (box), the minimum and maximum values (whisker), and outliers (black dots). (**F**) Pearson correlations of the total number of sequencing laboratories per country against key sequencing outputs.

By mapping and comparing the locations of specimen sampling laboratories to the sequencing laboratories, a number of aspects regarding the expansion of genomic surveillance on the continent became clear. First, even though several countries in Africa started sequencing SARS-CoV-2 in the first months of the pandemic, local sequencing capacity was initially limited. However, local sequencing capabilities slowly expanded over time, particularly after the emergence of VOCs (Fig. 2A). The fact that almost half of all SARS-CoV-2 sequencing in Africa was performed using the Oxford Nanopore technology (ONT), which is relatively low-cost compared to other sequencing technologies and better adapted to modest laboratory infrastructures, illustrates one component of how this rapid scale-up of local sequencing was achieved (fig. S5). Yet, to rely only on local sequencing would have thwarted the continent’s chance at a reliable genomic surveillance program. At the time of writing, there were 52/55 countries in Africa with SARS-CoV-2 genomes deposited in GISAID, however, there were still 16 countries with no reported local sequencing capacity (Fig. 2A) and undoubtedly many with limited capacity to meet demand during pandemic waves.

To tackle this, three centers of excellence and various regional sequencing hubs were established to maximize resources available in a few countries to assist in genomic surveillance across the continent. This sequencing is done either as the sole source of viral genomes for those countries (e.g., Angola, South Sudan and Namibia) or concurrently with local efforts to increase capacity during resurgences (Fig. 2B). Sequencing is further supplemented by a number of countries utilizing facilities outside of Africa. Ultimately, a mix of strategies from local sequencing, collaborative resource sharing among African countries and sequencing with academic collaborators outside the continent helped close surveillance blind spots (Fig. 2C). Countries in sub-Saharan Africa, particularly in Southern and East Africa, most benefited from the regional sequencing networks, while countries in West and North Africa often partnered with collaborators outside of Africa.

The success of pathogen genomic surveillance programs relies on how representative it is of the epidemic under investigation. For SARS-CoV-2, this is often measured in terms of the percentage of reported cases sequenced and the regularity of sampling. African countries were positioned across a range of different combinations of overall proportion and frequency of genomic sampling (Fig. 2D). While the ultimate goal would be to optimize both of these parameters, a lower proportion of sampling can also be useful if frequency of sampling is maintained as high as possible. For instance, South Africa and Nigeria, who have both sequenced ~1% of cases overall, can be considered to have successful genomic surveillance programs on the basis that sampling is representative over time, and has enabled the timely detection of variants (Beta, Eta, Omicron).

Additionally, for genomic surveillance to be most useful for rapid public health response during a pandemic, sequencing would ideally be done in real-time or in a framework as close as possible to that. We show a general trend of decreasing sequencing turnaround time in Africa (fig. S6), particularly from a mean of 182 days between October to December 2020 to a mean of 50 days over the same period a year later, although this does come with several caveats. First, we measure sequencing turnaround time in the most accessible manner, which is by comparing the date of sampling of a specimen to the date its sequence was deposited in GISAID. Generally, the genomic data potentially informs the public health response more rapidly than reflected here, particularly when it comes to local outbreak investigations or variant detection. This analysis is also confounded by various factors such as country-to-country variation in these trends (fig. S7), delays in data sharing, and potential retrospective sequencing, particularly by countries joining sequencing efforts at later stages of the pandemic. The most critical caveat is the fact that sequencing from the most recently collected samples (e.g., over the last six months) may still be ongoing. The shortening duration between sampling and genomic data sharing is nevertheless a positive takeaway, given that this data also feeds into continental and global genomic monitoring networks. Overall, the continental average delay from specimen collection to sequencing submission is 87 days with 10 countries having an average turnaround time of less than 60 days and Botswana of less than 30 days (fig. S8).

Most importantly in the context of optimizing genomic surveillance, we found that the route taken to sequencing impacts the speed of data generation. Local sequencing has significantly faster sequencing turn-around times of the three frameworks we investigated (median of 51 days), followed by sequencing within regional sequencing networks in Africa (median of 93 days) and finally outsourced sequencing to countries outside Africa (median of 113 days) (Fig. 2E). This finding strongly supports the investments in local genomic surveillance, to generate timely and regular data for local and regional decision making. Finally, we show that it is beneficial in several ways for countries to undertake genomic surveillance through several sequencing laboratories, rather than centralizing efforts. For instance, we estimate strong correlations between the numbers of sequencing laboratories per country with the total number of genomes produced by that country (method, correlation value), the total number of *epiweeks* for which sequencing data was produced (method, correlation value), and importantly, sequencing turnaround time (method, correlation value) (Fig. 2F).

With the increase in sequencing capacity on the continent, a decrease in the time taken to detect new variants was observed. For example, the Beta variant was identified in December 2020 in South Africa (*4*), but sampling and molecular clock analyses suggest the variant originated in September 2020. This three-month lag in detection means that a new variant, like Beta, has ample time to spread over a large geographic region prior to its detection. However, by the end of 2021, the time to detect a new variant was substantially improved. Phylogenetic and molecular clock analyses suggest that the Omicron variant originated around 9 October 2021 (95% Highest posterior density or HPD: 30 September - 20 October 2021) and the variant was described on 23rd November 2021 (*3*). Thus, Omicron was detected within ~5 weeks from origin compared to the Beta variant (~16 weeks) and the Alpha variant, detected in the UK (~10 weeks). More importantly, the time from sequence deposition to the WHO declaring the new variant a VOC was substantially shortened to 72 hours for the Omicron variant.

To interpret insights from the described genomic surveillance in Africa, it is important to understand the context of epidemiological reporting and sampling strategies utilized for sequencing on the continent (table S3). Most countries provided daily reports of newly recorded cases, while a few provided weekly and monthly reports. For most countries, surveillance was mainly focused on the major cities, suggesting potential cryptic circulation in rural areas. We find that at the onset of the pandemic, surveillance was focused on identification of imported cases from incoming travelers or local residents returning from various countries. As community transmissions began to emerge, the focus shifted toward regular surveillance and outbreak investigations. Together, these three strategies account for the vast majority of samples generated on the continent and analyzed here. As the pandemic progressed and vaccines were made available, some countries on the continent began to explore other sampling strategies such as reinfections, environmental samples such as waste water samples, and vaccine breakthrough cases to gain new insights into the evolutionary dynamics of SARS-CoV-2. The utility of sequencing for viral evolution tracking and VOC detection in the way described above is obviously also dependent on sampling proportions, especially within sampling for regular surveillance.

The speed of SARS-CoV-2 evolution has complicated sequencing efforts. Common methods of RNA sequencing include reverse transcription followed by double stranded DNA amplification using sequence-specific primer sets (*39*). Ongoing SARS-CoV-2 evolution has necessitated the continual evaluation and updating of these primer sets to ensure their sustained utility during genomic surveillance efforts. Here, we examined the current set of genomes to determine aspects of the sequencing that might be improved in the future. Many of the primer sets used were designed using viral sequences from the start of the pandemic and may require updating to keep pace with evolution. Indeed, the ARTIC primer sets are currently in version 4.1 (*40*). The Entebbe primer set was designed mid-2020 well into the first year of the epidemic and used an algorithm and design that accommodates evolution (*41*).

**Fig. 3.**
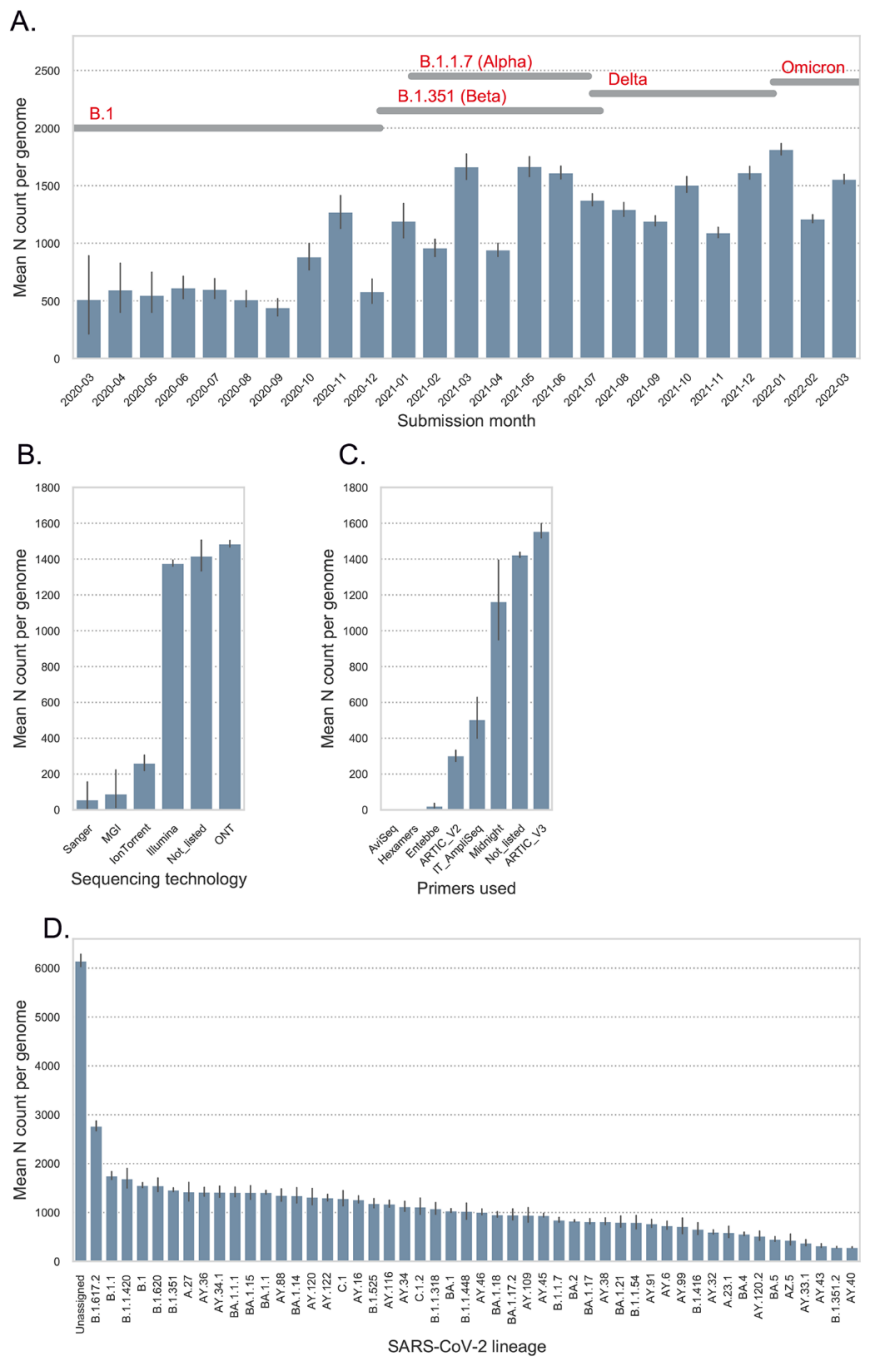
Genome gap analysis. (**A**) Shows the mean N count per genome by month of submission to GISAID. The dates for the detection of important SARS-CoV-2 lineages are indicated at the top of the figure. (**B**) Illustrates the mean N count per genome stratified by sequencing technology. (**C**) Shows the mean N count per genome stratified by the sequencing primers sets used. For panels A to C, error bars indicate 95% confidence intervals. (**D**) Mean N count per genome by lineage. The mean N data were stratified by SARS-CoV-2 lineages to investigate lineage-specific frequency of genome gaps, an indirect measure of primer mismatch. All lineages present at least 100 times in the genome data were presented.

**Fig. 4.**
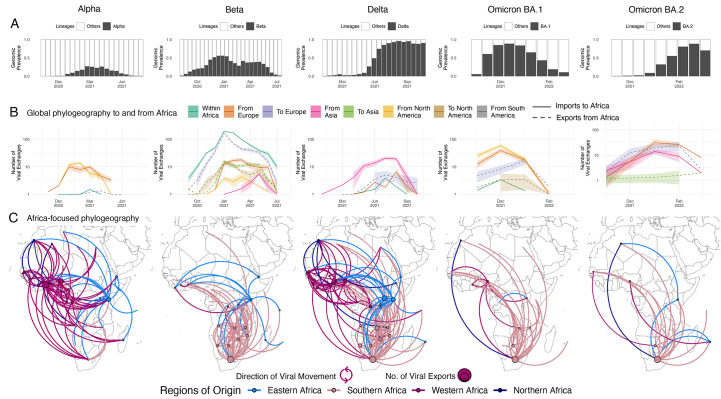
Inferred viral dissemination patterns of VOCs within Africa. (**A**) Genomic prevalence of VOCs Alpha, Beta, Delta and Omicron in Africa over time. (**B**) Inferred viral exchange patterns to, from and within the Africa continent for the four VOCs (Omicron as BA.1 and BA.2) based on case-sensitive phylogeographic inference. Introductions and viral transitions within Africa are shown in solid lines and exports from Africa are shown in dotted lines and these are colored by continent. The shaded areas around the lines represent uncertainty of this analysis from ten replicates (+/− s.d.). (**C**) Dissemination patterns of the VOCs within Africa, from inferred ancestral state reconstructions performed on Africa enriched datasets, annotated and colored by region in Africa. The countries of origin of viral exchange routes are also shown with dots and the curves go from country of origin to destination country in an anti-clockwise direction.

The effects of viral evolution on sequencing patterns can be seen with low median unspecified nucleotide (N)-values (a consequence of primer dropout or low coverage at that site) observed for the first 12 months of the epidemic with an increase from October 2020 (Fig. 3A). Additional challenges appear (indicated by increasing median N values) as the virus further evolved into Delta and Omicron lineages from January 2021 onward (Fig. 3A). Examining the role of sequencing technology, it appears that the two major technologies used (Illumina and ONT) have similar gap profiles (as measured by mean N count per genome) while Ion Torrent, MGI and Sanger show reduced mean N count per genome (Fig. 3B). Likely factors for this pattern are the primers used in sequencing, with primer choice playing a key role in the quantity of gaps (Fig. 3C). The mean N count per genome varied with viral lineage (Fig. 4D). There was a modest difference in mean N count per genome across the lineages. Lineages that returned no classification with Pangolin (“None”) showed the highest mean N count, suggesting that high mean N count per genome was probably the basis for failed classification. The more recent lineages Delta (e.g., AY.39, AY.75) and Omicron (BA.1.1, BA.2) also showed higher mean N count per genome consistent with virus evolution impairing primer function. This pattern is further explored in fig. S9 with position of gaps showing an enrichment in the genome regions after position 19 000 with frequent gaps disrupting the spike coding region.

### Phylogenetic insights into the rise and spread of variants of concern in Africa

During the first wave of infections in 2020 in Africa, as was the case globally, the majority of corresponding genomes were classified as PANGO B.1 (n=2 456) or B.1.1 viruses (n=1 329). Toward the end of 2020, more distinct viral lineages started to appear. The most important of which that impacted the African continent are: B.1.525 (n=797), B.1.1.318 (n=398) (*42*), B.1.1.418 (n=395), A.23.1 (n=358) (*15*, *29*, *31*, *33*), C.1 (n=446) (*29*), C.1.2 (n=300) (*31*), C.36 (n=305) (*30*, *43*), B.1.1.54 (n=287) (*15*, *29*, *31*, *33*), B.1.416 (n=272), B.1.177 (n=203), B.1.620 (n=138), and B.1.160 (n=61), (*32*) (fig. S10, A and B). Our discrete state phylogeographic inference from phylogenetic reconstruction of non-VOC African sequences and an equal number of external references revealed that African countries were primarily seeded by multiple introductions of viral lineages from abroad (mainly Europe) at the beginning of the pandemic. The observed pattern of non-VOC viral lineage movement then consistently shifted toward more intercontinental exchanges (fig. S10C). Mapping out the spatial routes of dissemination shows that various countries in all subregions of the continent acted as sources of these viral lineages at one point or another (fig. S10D). While uneven testing rates and proportions of samples sequenced on the continent may have influenced these inferences (discussed below), the results presented here are in line with the fact that these most predominant non-VOC lineages in Africa, except B.1.177, emerged and circulated widely in different sub-regions (Fig. 1).

Similar to the pandemic globally, VOCs became increasingly important in Africa toward the end of 2020. The Alpha, Beta, Delta and Omicron variants demonstrate many similarities as well as differences in the way they spread on the continent. For all these VOCs, we observe large regional monophyletic transmission clusters in each of their phylogenetic reconstructions in Africa (fig. S11). This suggests an important extent of continental dissemination within Africa. Alpha and Beta were epidemiologically important in distinct regions of the continent with Alpha primarily circulating in West, North and most of Central Africa, Beta in southern and most of East Africa, and only substantially co-circulated in a few countries such as Angola, Kenya, Comoros, Burundi and Ghana (Fig. 1 and fig. S12). However, we may not have enough resolution in the geospatial data to know how much they were truly co-circulating throughout these countries, or whether there were regional outbreaks of Alpha and Beta within these countries. In Kenya, for example, Beta was detected more in coastal regions, and Alpha more inland (*26*, *44*). In contrast, Delta and Omicron variants sequentially dominated the majority of infections on the entire continent shortly after their emergence (Fig. 4A and fig. S12).

The Alpha variant was first identified in December 2020 in the UK and has since spread globally. In Africa, Alpha was detected in 43 countries with evidence of community transmission, based on phylogenetic clustering, in many countries including Ghana, Nigeria, Kenya, Gabon and Angola (fig. S11). Discrete state maximum likelihood reconstruction from a globally case-sensitive genomic subsampling inferred at least 80 introductions (95% CI: 78 - 82) into Africa with the bulk of imports attributed to the US (>47%) and the UK (>25%) (Fig. 4B). Only 1% of imports into any particular African country were attributed to another African nation. Phylogeographic reconstruction enriched in African sequences revealed that of those, >85% of the intercontinental Alpha exchanges in Africa originated from West African countries (Fig. 4C). This occurred in spite of initial importations of the Alpha variant from Europe into all regions of the continent (fig. S13B), but is in line with Alpha having dominated circulation mostly in West Africa (fig. S12). In countries where Alpha was introduced but did not grow and cause an expansion of cases, this can be explained by competition with the already established Beta variant, which simultaneously circulated. The characteristics of multiple introductions of Alpha intro Africa and between African countries is similar to the spread of Alpha documented in the UK, Scotland and Ireland (*45*–*47*).

The second VOC, Beta, was identified in December 2020 in South Africa (*4*). However, sampling and molecular clock analyses suggest that the variant originated around September 2020 (fig. S11). At the end of 2020 and beginning of 2021, Beta was driving a second wave of infection in South Africa and quickly spread to other countries within the region. The concurrent introductions and spread of Alpha and other variants (Eta, A.23.1) in other regions of the continent may have reduced the Beta variant’s initial growth, limiting its spread to largely southern Africa, and to a lesser extent the East Africa region. Beta spread to at least 114 countries globally, including 37 countries and territories in Africa. For this variant, viral circulation and geographical exchanges occurred predominantly within the continent. Indeed, phylogeographic reconstruction from a globally case-sensitive sampling revealed that of the 810 (95% CI: 803 - 818) inferred introductions of the Beta variant into African countries, only 110 (95% CI: 105 - 115; 13%) were attributed to sources outside the continent (fig. S13C), while more than half of introductions were attributed to South Africa (63%) (Fig. 4C). This is in line with expectations as the variant originated in South Africa. Beyond southern Africa, most of the introductions back into the continent were attributed to France and other EU countries into the French overseas territories, Mayotte and Reunion, and other Francophone African countries. Africa-focused phylogeographic analysis revealed a similar spatial pattern showing southern countries as substantial sources of the variant, followed in small numbers by countries in East Africa (Fig. 4C).

The fourth VOC observed was Delta (*13*), which rose to prominence in April 2021 in India, where it fuelled an explosive second wave. Since its emergence, Delta was detected in >170 countries, including 37 African countries and territories (fig. S11). Our global case-sensitive subsampled analysis infers at least 100 (95% CI: 93 - 106) introductions of the Delta variant into Africa, with the bulk attributed to India (~72%), mainland Europe (~8%), the UK (~5%), and the US (~2.5%). Viral introductions of Delta also occurred from one African country to others, in 7% of inferred introductions. From our Africa-focused phylogeographic inferences, we infer that viral dissemination of Delta within Africa was not restricted to or dominated by any particular region unlike Alpha and Beta, but rather spread across the entire continent (Fig. 4C). Following introductions from Asia in the middle of 2021, Delta rapidly replaced the other circulating variants (Fig. 4A). For example, in southern African countries, the Delta variant rapidly displaced Beta and by June-2021 was circulating at very high (>90%) frequencies (*48*).

The latest VOC, Omicron, was identified and characterized in November 2021, in southern Africa (*3*). At the time of writing, the variant has been detected and caused waves of infections in >160 countries including 39 African countries and two overseas territories (fig. S11). Due to the genetic distance between them and their sequential epidemic expansion globally (rather than simultaneous), phylogenies were reconstructed separately for Omicron BA.1 and BA.2. Our discrete ancestral state reconstruction from a global case-sensitive sampling for Omicron BA.1 infers at least 55 (95% CI: 47 - 62) viral exports of BA.1 out of various African countries, of which 31 (95% CI: 25 - 36) were toward Europe and 8 (95% CI: 6 - 10) toward North America (Fig. 4B). Following explosive expansion of Omicron around the world, we inferred even more reintroductions of the variant back into Africa, at least 69 (95% CI: 60 - 78) from Europe and 102 (95% CI: 92 - 112) from North America (Fig. 4B). From our Africa-focused phylogeographic reconstructions, we determine that, as with Delta, routes of dissemination of this variant involved all regions of the continent spatially (Fig. 4C). Yet, ~75% of all BA.1 viral movement volume in Africa happened between southern African countries, likely due to rapid epidemic expansion in the region soon after its detection (*3*). Omicron BA.2’s reach in Africa was limited at the time of writing, with only 3 260 sequences from 19 countries attributed to BA.2 on GISAID (Date of access: 2022-03-31) (15% of all Omicron sequences from Africa). Our discrete ancestral state reconstruction from a global case-sensitive sampling for Omicron BA.2 infers at least 68 (95% CI: 53 - 84) viral exports out of African countries, of which the majority were toward Europe (~88%) (Fig. 4B). We also infer at least 99 (95% CI: 87 - 109) separate introduction or reintroduction events of BA.2 back into African countries, of which ~65% are from Europe and ~30% from Asia, primarily from India (Fig. 4B). This is consistent with India having experienced one of the earliest large BA.2 waves globally. In the context of global incidence of BA.2, this case-sensitive phylogeographic analysis revealed that only 0.01% of viral movements of this lineage globally happened from one African country to another. Our Africa-focused analysis inferred a similar pattern of BA.2 spatial diffusion within African to BA.1 (Fig. 4C). However, given that this accounted for such a small percentage of global BA.2 movements, BA.2 diffusion from one African country to another is unlikely to have had a significant impact on epidemiological expansion, compared to introductions from Asia, Europe or North America.

Globally, dissemination of the SARS-CoV-2 virus throughout the pandemic was intricately linked with human mobility patterns (*49*–*53*). To determine the validity of the VOC movement patterns that we infer into and within the Africa continent in this study, we compared viral import and export events to and from South Africa with travel to the country. In December 2020, the UK accounted for the 5th highest number of passengers entering South Africa, while other countries with the top 9 sources of travellers were all neighboring countries in southern Africa (fig. S14A). Considering that incidence of the Alpha variant was insignificant in the region, this supports our inference of the UK contributing 60% of Alpha introductions to South Africa (fig. S15A). In March 2021, the US, Germany, the UK and India were among the top 12 sources of travellers to South Africa behind 8 African countries (fig. S14B). During this time of Delta dissemination globally, we infer that ~90% of introductions of Delta into South Africa originated in the UK, the US and India (fig. S15B). At the end of 2021, most introductions or re-introductions of Omicron to the country came from the UK, the US or Botswana, corresponding to locations of both high Omicron incidence at the time, and high numbers of passengers to South Africa (figs. S14C and S15C). These travel patterns also fit the findings that ~89%,~70% and ~75% of Beta, Delta and Omicron exports respectively from South Africa to other African countries were directed to locations of southern Africa (figs. S14, D and E, and S15, D and E).

## Discussion, limitations and conclusions

By April 2020, a total of 20 African countries were able to sequence the virus within their own borders. This was largely made possible by other pre-existing sequencing efforts on the continent focused on other human pathogens (e.g., HIV, TB, Ebola and H1N1). However, these efforts were quickly limited by global supply chain issues and in many countries sequencing efforts dramatically slowed down or stopped toward the end of 2020. In order to facilitate more sequencing on the continent over the course of the past year (April 2021 - March 2022) the Africa CDC and partners invested heavily to support genomic surveillance on the continent. This included the transfer of 24 new sequencing platforms (including MinIon, GridIon, MiSeq and NextSeq), the distribution of reagents and flow cells to support the sequencing of 100 000 positive samples, the training of >230 students and technicians in wet laboratory and bioinformatic techniques and additional grants to support 10 regional sequencing hubs. This investment has started bearing fruit and should be intensified as the virus continues to evolve, requiring the adaptation of methodologies locally on the continent to keep pace with the emergence of variants. The continued development of sequencing protocols in Africa is of crucial importance (*41*, *54*, *55*) given the number of variants and lineages that emerged in, and were introduced to, the continent. In Northern Africa, the SARS-CoV-2 pandemic was caused by waves of infections that were similar to those seen in Europe (first wave = B.1 descendants, second wave = Alpha, third wave = Delta and forth wave = Omicron), in southern Africa the pattern was similar but with a Beta wave instead of an Alpha one. In East Africa, the pandemic was more complex, involving both Alpha and Beta as well as its own lineage A.23.1 before the arrival of Delta and Omicron. Central Africa experienced epidemic patterns sometimes mirroring East Africa and other times southern Africa. In West Africa, Eta made a significant contribution to both a second wave (together with alpha) and a third wave (together with Delta). The factors that resulted in these regional differences are not clear but could be due to differences in human mobility, founder effects, competition between lineages or the immunity induced by earlier waves in a region.

Public health benefits of such broadly inclusive genomic surveillance are manifold. The most prominent insight from this expanded genomic surveillance in Africa has been an early warning capacity for the world following the detection of new lineages and variants, most recently relevant in the detection of Omicron BA.1, BA.2, BA.3, BA.4 and BA.5 sub-variants (*3*, *4*, *34*). Furthermore, the reporting of local SARS-CoV-2 sequences made the epidemic more immediate to the Ministries of Health from the reporting African countries. It became clear early on that the viral evolution is global and the transmission of the virus is extremely rapid which guided mitigation strategies. The generation and the availability of local sequences also validated local diagnostics and allowed investigators to determine if nucleic acid based diagnostics in use could still detect local variants. The detection of SARS-CoV-2 in returning travellers and truck drivers indicated routes that the virus might be using to enter a country and guided early efforts to slow the virus entry and gain time to establish vaccination plans. Later the difficulty of stopping the virus at borders combined with the data that the variants were already in community circulation allowed public health officials to focus efforts and limited resources on vaccination rather than on border controls. The detection and reporting of the more recent lineages with enhanced transmission (i.e., Omicron) and the ability to bypass existing immunity is important information and an early alert to the public health officials globally that the epidemic was still proceeding. As the pandemic progresses in an evolving global context, we provide evidence that with each new variant, transmission dynamics are changing and the use of sequencing with phylogenetics could potentially alter decisions of public health measures. For example, the demonstrated shift away from regional dynamics of Alpha and Beta toward more global patterns with Delta and Omicron can provide insights to public health officials as they anticipate epidemic developments locally. With Omicron it became clear that although the variant expanded first in Africa, the continent ultimately had a minimal role in global dissemination, and continental expansion beyond southern Africa was most influenced by external introductions, in contrast to the Beta variant. All of these public health benefits to sequencing SARS-CoV-2 is primarily amplified, as we show in this study, if the sequencing can be conducted locally within a country, which strongly supports the continued investment into pathogen sequencing on the continent.

In spite of the recent successful expansion of genomics surveillance in Africa, additional work remains necessary. Even with the Africa CDC - Africa PGI’s and other investments, there are still 16 countries with no sequencing capacity within their own borders. These countries' only option is to send samples to continental sequencing hubs or to centers outside of the continent, which increases the turnaround times and limits the utility of genomic surveillance for public health decision making. Secondly, not all countries are willing to share data openly in a timely fashion for fear of being subject to travel bans or restrictions which could bring substantial economic harm. Such hesitancy has obvious potential ramifications for the future of genomic surveillance on the continent. Furthermore, with the expansion of sequencing on the continent there is a growing need for more bioinformatics support and knowledge to allow investigators to analyze and report their data in a reasonable timeframe that makes it useful for public health response. It is also clear the SARS-CoV-2 sequencing primers are not a static development and may require updating as the virus evolves. A number of research groups have been addressing the SARS-CoV-2 sequencing primer questions. Issues of gaps in the genomes due to missing amplicons have been discussed (*56*, *57*). The ARTIC primer set has gone through a number of revisions to accommodate virus evolution (*39*, *40*). Additional longer amplicon methods have been published (*58*–*60*) including methods to use a subset of ARTIC primers (*61*).

The patterns we describe here are of course limited to reported cases, and applies to both the phylogeographic as well as the epidemiology inferences. As such, the results need to be interpreted with these limitations in mind. Our primary phylogeographic inference relied on a sampling strategy considering all high quality African sequences and an equal number of external references. Though this strategy has the advantage of placing all African sequences in a phylogenetic context, it introduces a bias when applied to discrete ancestral state reconstruction as more internal nodes are inferred to be from Africa. To address this we performed an even sampling of global cases, based on reported case counts through time, to compare against our over sampled inference. The even sampling approach has the benefit that the discrete ancestral state reconstruction is not biased by uneven sampling. Comparing the two there are obvious differences, most notably that the number of inferred introductions into Africa is proportional to sampling proportions (fig. S16), as we no longer consider all African sequences but just a small subset against a global sample. However, inferences from the two approaches correspond well with one another. For example, considering Alpha we still observed the vast majority of introductions into Africa to originate from Western Europe. Patterns of dissemination within Africa are more robustly comparable between the two, for instance that countries in West Africa were the biggest source of Alpha within the continent. High concordance between the two inference methods were also observed for other VOCs for dispersal routes within Africa which gives us confidence in the inferred patterns we observe here. Although we represent an inference based on over sampling and case sensitive sampling, it is currently not possible to explore how under sampling affects the phylogeographic reconstruction due to uneven testing rates. Additionally, the robustness of the phylogeographic inference can also be affected by the underlying methodology used. Broad consensus would favor the use of Bayesian methods for phylogeographic reconstruction, which is often considered to be the “gold standard” in the field. The main drawbacks of Bayesian methods are that they can only be applied to a relatively small number of sequences at a time (<1,000) and are extremely computationally and time intensive. Given the explosion of sequence data over the past two years, the scientific community will have to adapt or put forth new analytical methods to fully capitalize on the global sequencing efforts for SARS-CoV-2.

Despite our best attempts to consider and minimize genomic sampling bias, the accuracy of the resulting phylogenetic inferences is limited by the available epidemiological and genomic data, leading to unaccounted biases in the estimates of viral movements. This includes limited testing and subsequent sequencing in many African countries. Although the percentage of reported cases sequenced in African countries (0.01 - 10%, mean = 1.27%) is not far from global figures (0.01-16%, mean = 1.31%), testing rates and infection-to-detection ratios in Africa were some of the lowest globally (*38*, *62*). Together with estimates of excess mortality being as much as 20-fold more than the reported numbers in African countries (*63*), these are strong indications of undetected and underreported epidemic sizes in Africa, leading to undersampling of genomic data (*62*) and thus underestimates of viral exchange inferences in our study. Some countries with no publicly available SARS-CoV-2 sequences are by definition completely missing in our inference. This in turn means that inferred routes of viral transmission within Africa could be missing important intermediate locations, although this is potentially true around the world. Nevertheless, we believe that the viral movement inferences that we discuss in this study provide a likely qualitative description of the patterns of SARS-CoV-2 migration into, out of, and within Africa.

Finally, we should also mention uneven sequencing and reporting standards across the different laboratories on the continent - and globally, for that matter. Different groups use different measures for what constitutes a high quality sequence (e.g., 70% vs 80% sequence coverage) or using different sequencing depth coverage. This lack of standardization globally complicates the direct comparison of sequences that may have been submitted to GISIAD using different criteria further biasing any inference. Given the sheer size of SARS-CoV-2 sequencing, with ~10 million whole genome sequences shared on the GISAID database (31st March 2022), there is an urgent need for global standards with regards to sequence quality and associated metadata.

In conclusion, Africa needs to continue expanding genomic sequencing technologies on the continent in conjunction with diagnostics capabilities. This holds true not just for SARS-CoV-2 but for other emerging or re-emerging pathogens on the continent. For example, WHO announced in February 2022 the re-emergence of wild polio in Africa, while sporadic influenza H1N1, measles and Ebola outbreaks continue to occur on the continent. The Africa CDC has estimated that over 200 pathogen outbreaks are reported across the continent every year. Beyond the current pandemic, continued investment in diagnostic and sequencing capacity for these pathogens could serve the public health of the continent well into the 21st century.

## Methods and methods

### ​​Ethics statement

This project relied on sequence data and associated metadata publicly shared by the GISAID data repository and adhere to the terms and conditions laid out by GISAID (*16*). The African samples processed in this study were obtained anonymously from material exceeding the routine diagnosis of SARS-CoV-2 in African public and private health laboratories. Individual institutional review board (IRB) references or material transfer agreements (MTAs) for countries are listed below.

Angola - (MTA - CON8260), Botswana - Genomic surveillance in Botswana was approved by the Health Research and Development Committee (Protocol HPDME 13/18/1), Egypt - Surveillance in Egypt was approved by the Research Ethics Committee of the National Research Centre (Egypt) (protocol number 14 155, dated March 22, 2020), Kenya - samples were collected under the Ministry of Health protocols as part of the national COVID-19 public health response. The whole genome sequencing study protocol was reviewed and approved by the Scientific and Ethics Review Committee (SERU) at Kenya Medical Research Institute (KEMRI), Nairobi, Kenya (SERU protocol #4035), Nigeria – (NHREC/01/01/2007), Mali - study of the sequence of SARS-CoV-2 isolates in Mali - Letter of Ethical Committee (N0-2020 /201/CE/FMPOS/FAPH of 09/17/2020), Mozambique - (MTA - CON7800), Malawi - (MTA - CON8265), South Africa - The use of South African samples for sequencing and genomic surveillance were approved by University of KwaZulu-Natal Biomedical Research Ethics Committee (ref. BREC/00001510/2020); the University of the Witwatersrand Human Research Ethics Committee (HREC) (ref. M180832); Stellenbosch University HREC (ref. N20/04/008_COVID-19); the University of the Free State Research Ethics Committee (ref. UFS-HSD2020/1860/2710) and the University of Cape Town HREC (ref. 383/2020), Tunisia - for sequences derived from sampling in Tunisia, all patients provided their informed consent to use their samples for sequencing of the viral genomes. The ethical agreement was provided to the research project ADAGE (PRFCOVID19GP2) by the Committee of protection of persons (Tunisian Ministry of Health) under the reference (CPP SUD N 0265/2020), Uganda - The use of samples and sequences from Uganda were approved by the Uganda Virus Research Institute - Research and Ethics Committee UVRI-REC Federalwide Assurance [FWA] FWA No. 00001354, study reference - GC/127/20/04/771 and by the Uganda National Council for Science and Technology, reference number - HS936ES) and Zimbabwe (MTA - CON8271).

### Epidemiological and genomic data dynamics

We analyzed trends in daily numbers of cases of SARS-CoV-2 in Africa up to 31st March 2022 from publicly released data provided by the Our World in Data repository for the continent of Africa (https://github.com/owid/covid-19-data/tree/master/public/data) as a whole and for individual countries (*2*). To provide a comparable view of epidemiological dynamics over time in various countries, the variable under primary consideration for Fig. 1 was ‘new cases per million (smoothed)’. To calculate the genomic sampling proportion and frequency for each country for Fig. 2, the total number of recorded cases at 31st March was considered, as well as the total length of time for which each country has recorded cases of SARS-CoV-2.

Genomic metadata was downloaded for all African entries on GISAID for the same time period (date of access: 31st March 2022). From this, information extracted from all entries for this study included: date of sampling, country of sampling, viral lineage and clade, originating laboratory, sequencing laboratory, and date of submission to the GISAID database. The geographical locations of the originating and sequencing laboratories were manually curated. Sequences originating and sequenced in the same country were defined as locally sequenced, irrespective of specific laboratory or finer location. Sequences originating in one African country and sequenced in another were defined as sequenced within regional sequencing networks. Sequences sequenced in a location not within Africa were labeled as sequenced outside Africa. Sequencing turnaround time was defined as the number of days elapsed from specimen collection to sequence submission to GISAID. Sequencing technology information for all African entries was also downloaded from GISAID on 31st March 2022.

### Primer choice and sequencing outcomes

All SARS-CoV-2 genomes from African countries were retrieved from GISAID (*16*) for submission dates from 1 December 2019 to 31st March 2022 yielding 100 470 entries. Associated metadata for the entries were also retrieved, including collection date, submission date, country, viral strain and sequencing technology. Data on the primers used for the sequencing were requested from investigators and yielded primer data for 13 973 of the entries (~13%). The total N (bases with low sequence depth) per genome were counted, results from which were then used for genome quality analysis and visualization. Gap locations in the genomes were mapped and visualized compared to the original Wuhan strain (*64*).

### Phylogenetic investigation

All African sequences on the GISAID sequence database (*16*) were downloaded on the 31st of March 2022 (n=100 470). Of this, Alpha accounted for 3 851 sequences, Beta accounted for 14 548 sequences, Delta accounted for 35 027 sequences, Omicron for 21 708, while 25 336 sequences were classified as none-VOCs. Prior to any phylogenetic inference we performed some quality assessment on the sequences to exclude incomplete or problematic sequences as well as sequences lacking complete metadata. Briefly, all African sequences were passed through the NextClade analysis pipeline (*65*) in order to identify and exclude: (*i*) sequences missing >10% of the SARS-CoV-2 genome, (*ii*) sequences that deviate by >70 nucleotides from the Wuhan reference strain, (*iii*) sequences with >10 ambiguous bases, (*iv*) clustered mutations, and (*v*) sequences flagged with private mutations by NextClade. Additionally, Omicron variants were screened for traces of viral recombination with RDP5.23 (*66*) using default settings and a p-value of ≤0.05 as evidence of recombination. A large number of sequences were removed (n=57 421) with incomplete sequences (<90% genome coverage) being the biggest contributor. This produced a final African dataset of 43 049 high quality African sequences. Due to the sheer size of the dataset we opted to perform independent phylogenetic inferences on the main VOCs (Alpha, Beta, Delta and Omicron BA.1 and BA.2) that have spread on the African continent, as well as a separate inference for all non-VOC SARS-CoV-2 sequences.

In order to evaluate the spread of the virus on the African continent we aligned the African datasets against a large number of globally representative sequences from around the world. Due to the oversampling of some variants or lineages we performed a random down sampling while retaining the oldest two known variants from each country. Reference sequences were respectively aligned with their African counterparts independently with NextAlign (*65*). Each of the alignments were then used to infer maximum likelihood (ML) tree topologies in FastTree v 2.0 (*67*) using the General Time Reversible (GTR) model of nucleotide substitution and a total of 100 bootstrap replicates (*68*). The resulting ML tree topologies were first inspected in TempEst (*69*) to identify any sequences that deviate more than 0.0001 from the residual mean. Following the removal of potential outliers in R with the ape package (*70*), the resulting ML-trees were then transformed into time calibrated phylogenies in TreeTime (*71*) by applying a rate of 8x10e-4 substitution per site per year (*72*) in order to transform the branches into units of calendar time. Time calibrated trees were then visualized along with associated metadata in R using ggtree (*73*) and other packages.

We performed a basic viral dispersal analysis for each of the VOCs (excluding Gamma), as well as for the non-VOC dataset. Briefly, a migration model was fitted to each of the time calibrated tree topologies in TreeTime, mapping the country location of sampled sequences to the external tips of the trees. The *mugration* model of TreeTime also infer the most likely location for internal nodes in the trees. Using a custom python script we could then count the number of state changes by iterating over each phylogeny from the root to the external tips. We count state changes when an internal node transitions from one country to a different country in the resulting child-node or tip(s). The timing of transition events is then recorded which serve as the estimated import or export event. To infer some confidence around these estimates, we performed ten replicates for each of the dataset by random selection from the 100 bootstrap trees. Due to the high uncertainty in the inferred locations for deep internal nodes in the trees we truncated state changes to the earliest date of sampling in each dataset. All data analytics were performed using custom python and R scripts and results visualized using the ggplot libraries (*74*). Such phylogeographic methods are always subject to uneven sampling through time (i.e., over the course of the pandemic) and through space (by sampling location). To address this we have performed a case sensitive analysis to investigate the effects of oversampling African locations on the inferred number of viral introductions. Furthermore, in a previous analysis (*15*) we performed a sensitivity analysis to address some of these issues and found no substantial variations in estimates.

### Case sensitive phylogeographic inference

To address the potential over sampling of African sequences relative to global reference in the above mentioned analyses we performed another phylogeographic inference on subsamples based on global case counts to try and eliminate oversampling bias in our inference. To this end, we considered all high quality sequences for each of the VOCs (Alpha, Beta, Delta and Omicron BA.1 and BA.2) globally over the same sampling period (till 31st of March 2022). We used subsampler (https://github.com/andersonbrito/subsampler) to generate subsamples for each variant based on globally reported cases. In short, subsampler uses a case count matrix of daily cases, along with the fasta sequences and GISAID associated metadata to sample a user defined number of sequences. For each VOC and for BA.1 and BA.2 we performed 10 samplings using different number seeds in order to sample datasets of ~20 000. Once again, sampled sequences were screened for viral recombination as described above and sequences with signs of recombination were removed. Subsampler has the added advantage that it disregards poor quality sequences (e.g., <90% coverage) and sequences with missing metadata (e.g., exact date of sampling). Each dataset was then subjected to the same analytical pipeline as mentioned above to infer the viral transitions between Africa and the rest of the world.

### Regional and country specific NextStrain builds

In order to investigate more granular changes in lineage dynamics within a specific country or region in Africa we utilized the NextStrain pipeline (https://github.com/nextstrain/ncov) to generate the regional and country-specific builds for African countries (*75*). First, all sequence data and metadata were retrieved from the GISAID sequence database and filtered for Africa based on the 'region' tab, for inclusion in regional- and country-specific African builds. For country-specific builds ~4 000 sequences from a given country were randomly selected and analyzed against ~1 000 randomly selected sequences from the Africa 'nextregions' records that do not match the focal country of interest. For region specific (e.g., West Africa), ~4 000 sequences from the focal region are selected at random and analyzed against ~1 000 randomly selected sequences from the Africa 'nextregions' records that do not match the focal region of interest. The methodological pipeline for NextStrain is well documented and performs all analyses within one workflow, including filtering of sequences, alignment, tree inference, molecular clock and ancestral state reconstruction. For more information please visit, https://docs.nextstrain.org/en/latest/index.html.

All region- and country-specific builds are regularly updated to keep track of the evolving pandemic on the continent. All builds are publicly available under the links provided in tables S1 and S2 as well as on the NextStrain webpage (https://nextstrain.org/sars-cov-2/#datasets).

## Supplementary Materials

### This PDF file includes:

Figs. S1 to S16 Tables S1 to S4 Reference (*77*)

### Other Supplementary Material for this manuscript includes the following:

MDAR Reproducibility Checklist
