# Supplementary material for: The evolving SARS-CoV-2 epidemic in Africa: Insights from rapidly expanding genomic surveillance
Source: Science. 2022 Sep 15:eabq5358. doi: 10.1126/science.abq5358 (PMC9529057; doi:10.1126/science.abq5358)
Supplement: Supplementary file 1 — Figs. S1 to S16 Tables S1 to S4 Reference (77) [file science.abq5358_sm.pdf]

Science
AAAS

Supplementary Materials for

The evolving SARS-CoV-2 epidemic in Africa: Insights from rapidly expanding genomic surveillance

Houriiyah Tegally *et al.*

Corresponding authors: Tulio de Oliveira, tulio@sun.ac.za; Eduan Wilkinson, ewilkinson@sun.ac.za

DOI: 10.1126/science.abq5358

The PDF file includes:

Figs. S1 to S16
Tables S1 and S2
References

Other Supplementary Material for this manuscript includes the following:

MDAR Reproducibility Checklist
Tables S3 and S4

Supplementary Materials

Supplementary Figures

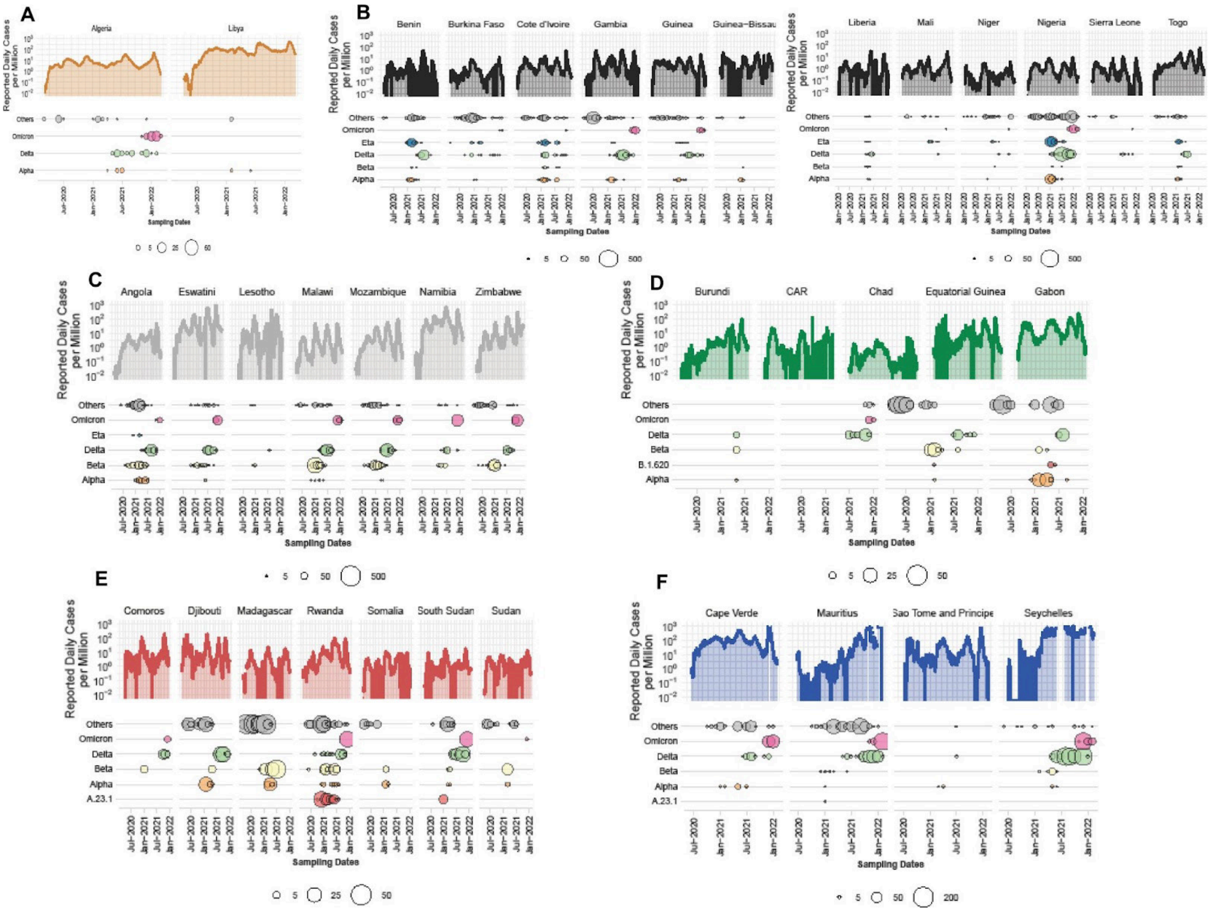

Supplementary Figure S1: Epidemiological progression of the COVID-19 pandemic in all African countries overlaid with the distribution of VOCs, the Eta VOI and other lineages through time (size of circles proportional to the number of genomes sampled per month for each category). The graphs show a breakdown of new cases per million and monthly sampling of VOCs, regional variant or lineage of interest and other lineages for all African countries not shown in Figure 1, grouped by region: A) North Africa, B) West Africa, C) Southern Africa, D) Central Africa, E) East Africa, F) Cape Verde, Mauritius, Sao Tome and Principe and Seychelles, from the beginning of the pandemic to February 2022.

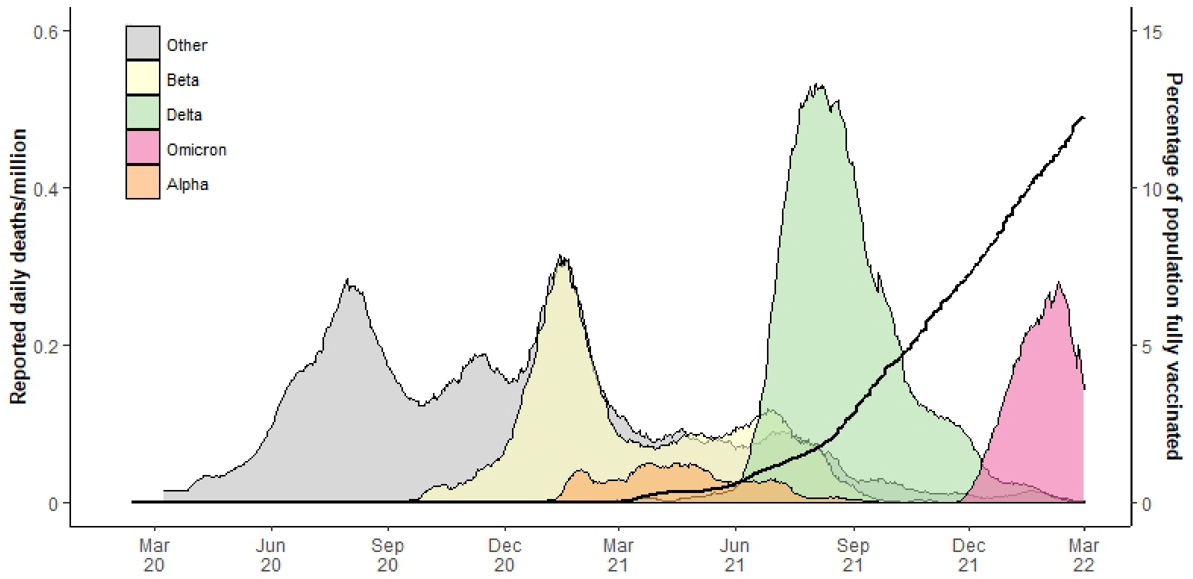

Supplementary Figure S2: Daily reported deaths per million people attributed to each variant of concern with vaccination coverage across Africa. The daily reported deaths were calculated to be attributed to each variant of concern based on the proportion of the variants in genomic surveillance data available from GISAID. We applied an assumption of a 20 day time lag from infection to death (77). Alpha caused the lowest peak in reported mortality in Africa, while the largest is attributed to Delta which coincided with the beginning of vaccinations on the continent. The impact of Omicron on mortality is to a much lesser extent than Delta, the peak of Omicron mortality occurred when approximately 12% of the African population was fully vaccinated (completed the initial vaccination protocol of 2 doses for most vaccines, 1 or 3 for some manufacturers).

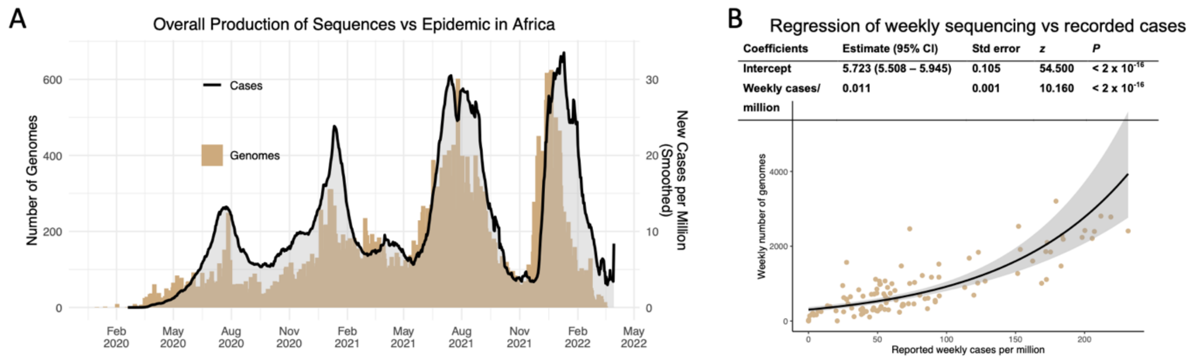

Supplementary Figure S3: Trends of genomic sequencing and epidemic size in Africa. A) Corresponding daily progression of genomic sequence production and epidemic size in Africa. B) Regression of weekly sequencing against recorded cases in Africa. We used a negative binomial regression, with a log link function and maximum likelihood estimation of theta, to investigate the relationship between the number of SARS-CoV-2 genomes produced per week and the weekly number of reported COVID-19 cases in Africa. The regression results indicated a significant positive effect of case numbers on the number of genomes produced, with each one-unit increase in reported weekly cases per million, the expected log count of genomes produced increased by 0.011 (θ = 2.23).

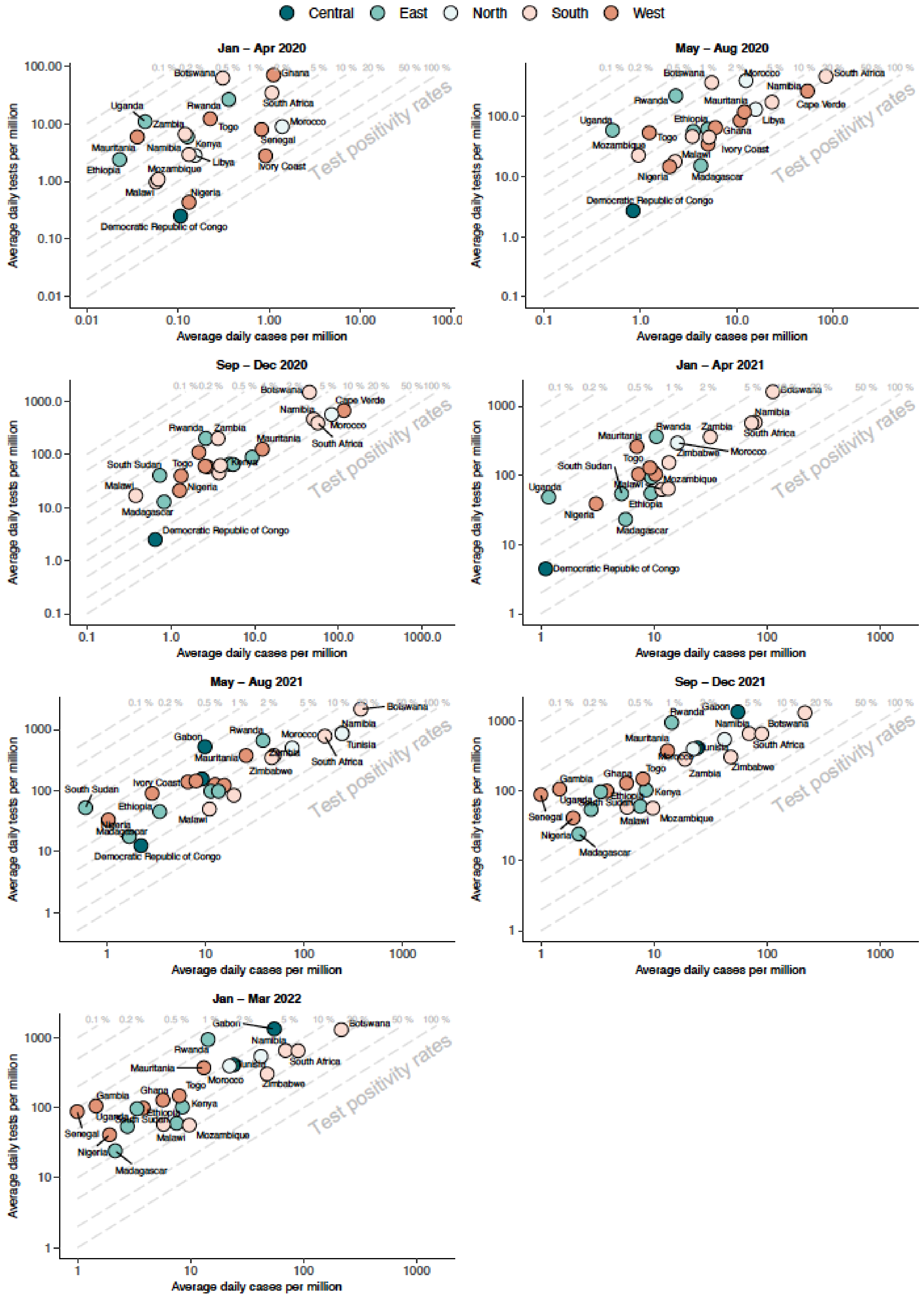

Supplementary Figure S4: COVID-19 testing rates in Africa against recorded cases per million for countries with available data. The extent of testing (average daily number of tests) is shown relative to the size of outbreaks (average daily number of reported cases) per million people per country in Africa. Data is obtained from Our World in Data (OWID). Panels span four-month intervals over the first two years of the epidemic. Test positive rates are displayed in gray segmented lines (0.02 – 100%). Testing efforts of most countries varied considerably

over the course of the epidemic with several southern African countries demonstrating generally the highest positive rate (highest testing rate per case) over the last year of the epidemic. Additionally, the spread of points mostly reduced to between 1 and 20% positive rate from January 2021 to March 2022. Points are shown for countries reporting the relevant data and with an average daily number of tests greater than zero for the relevant period. Axes are log scaled.

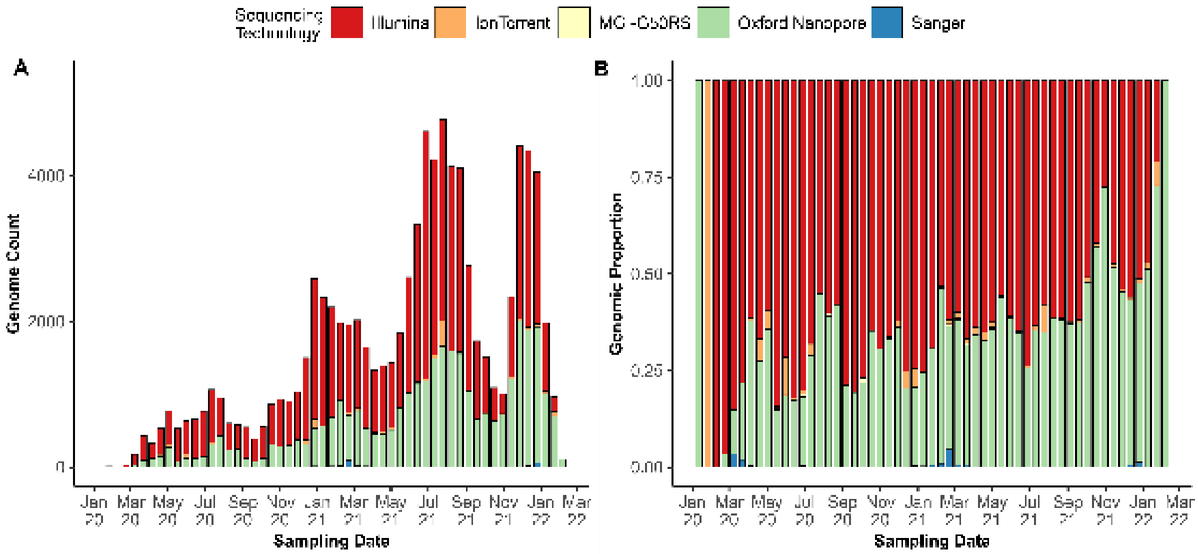

Supplementary Figure S5: The progression of sequencing technologies used for SARS-CoV-2 sequencing in Africa. Sequences are aggregated by associated sequencing technologies as reported on GISAID. Panel A represent the raw number of sequence generated using the five different technologies, while panel B represent the proportion of sequences generated by technology over time.

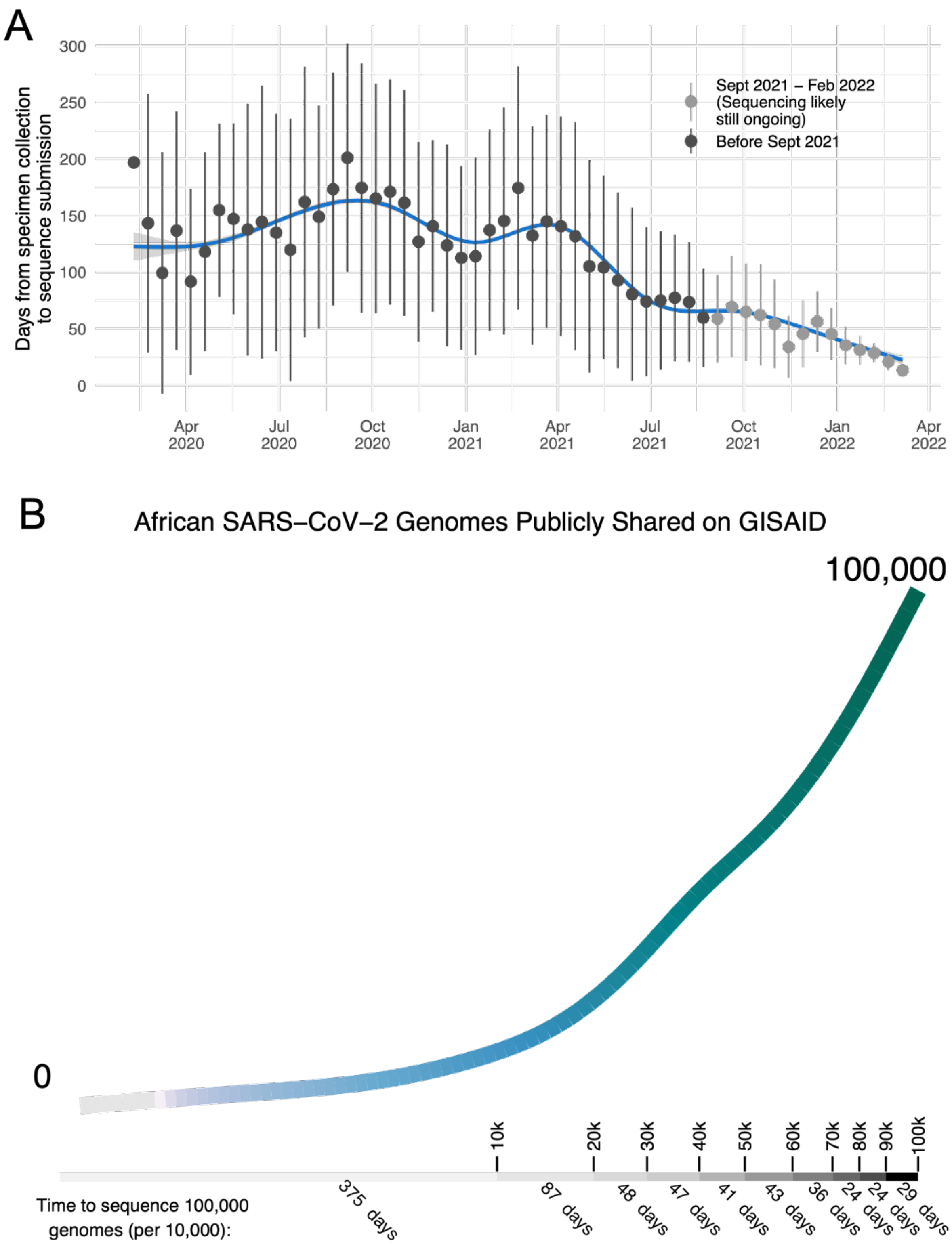

Supplementary Figure S6: Trends of sequencing turnaround time in Africa. A) Overall decreasing trend in sequencing turnaround time in Africa, with the caveat that sequencing for recent samples may not be completed. B) Number of days taken to sequence 10 000 sequences increment in Africa, until the 100 000 sequences milestone.

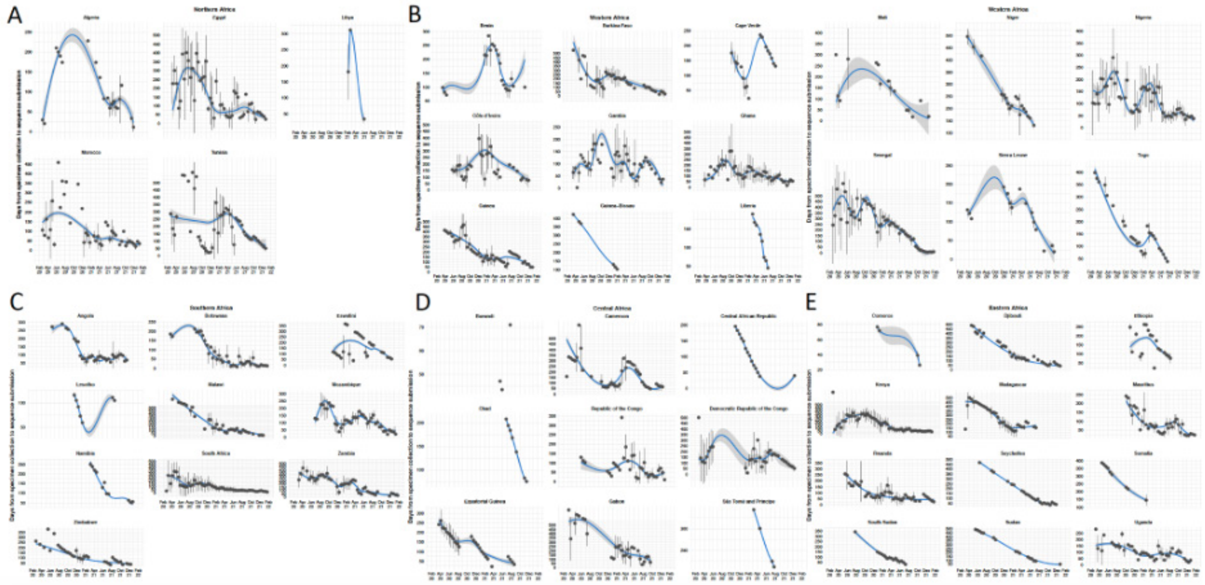

Supplementary Figure S7: Sequencing turnaround time progression per country in each region of Africa. Trends are shown for A) North Africa, B) West Africa, C) Southern Africa, D) Central Africa, and E) East Africa

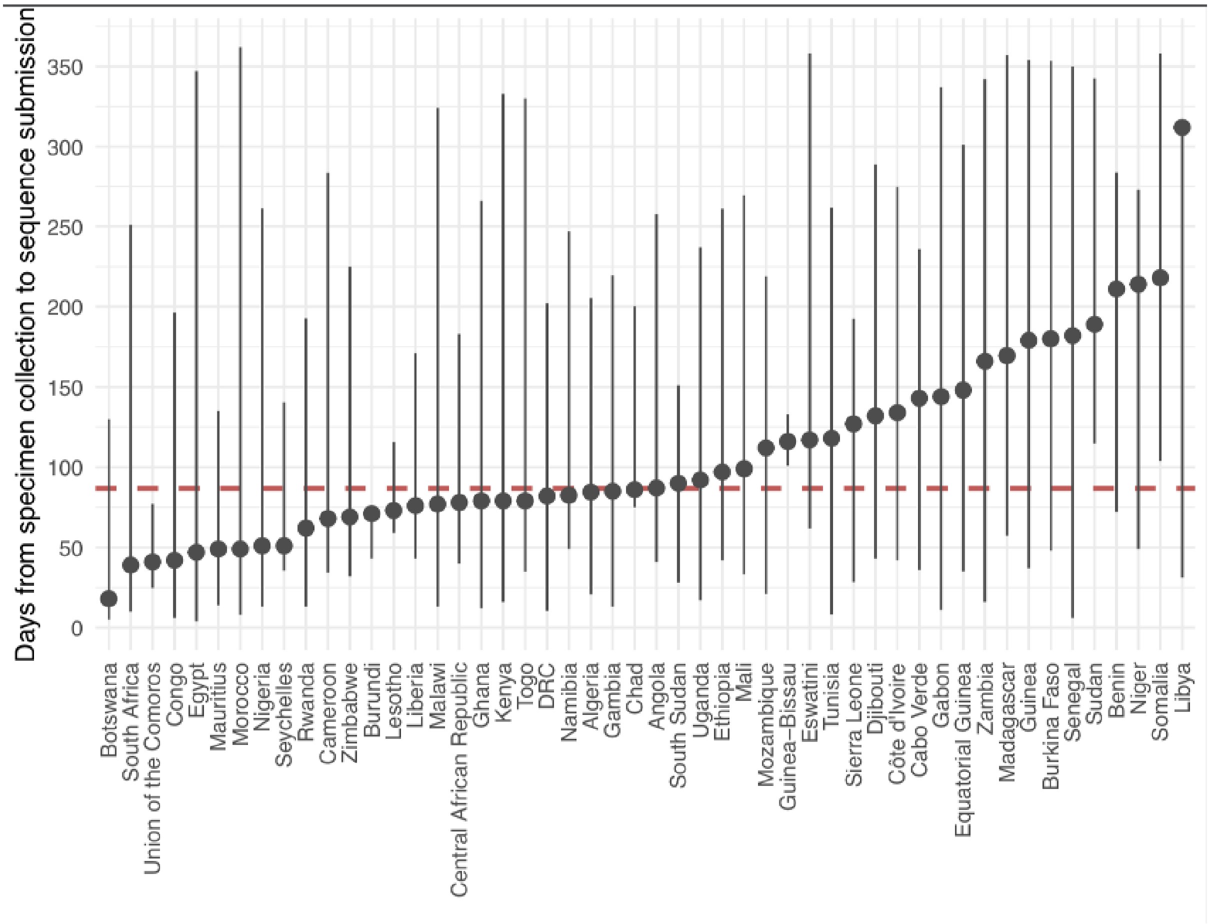

Supplementary Figure S8: Sequencing turnaround time average by country. The circles indicate the mean number of days between specimen collection and sequence submission to GISAID and the bars indicate the distribution of values for each country.

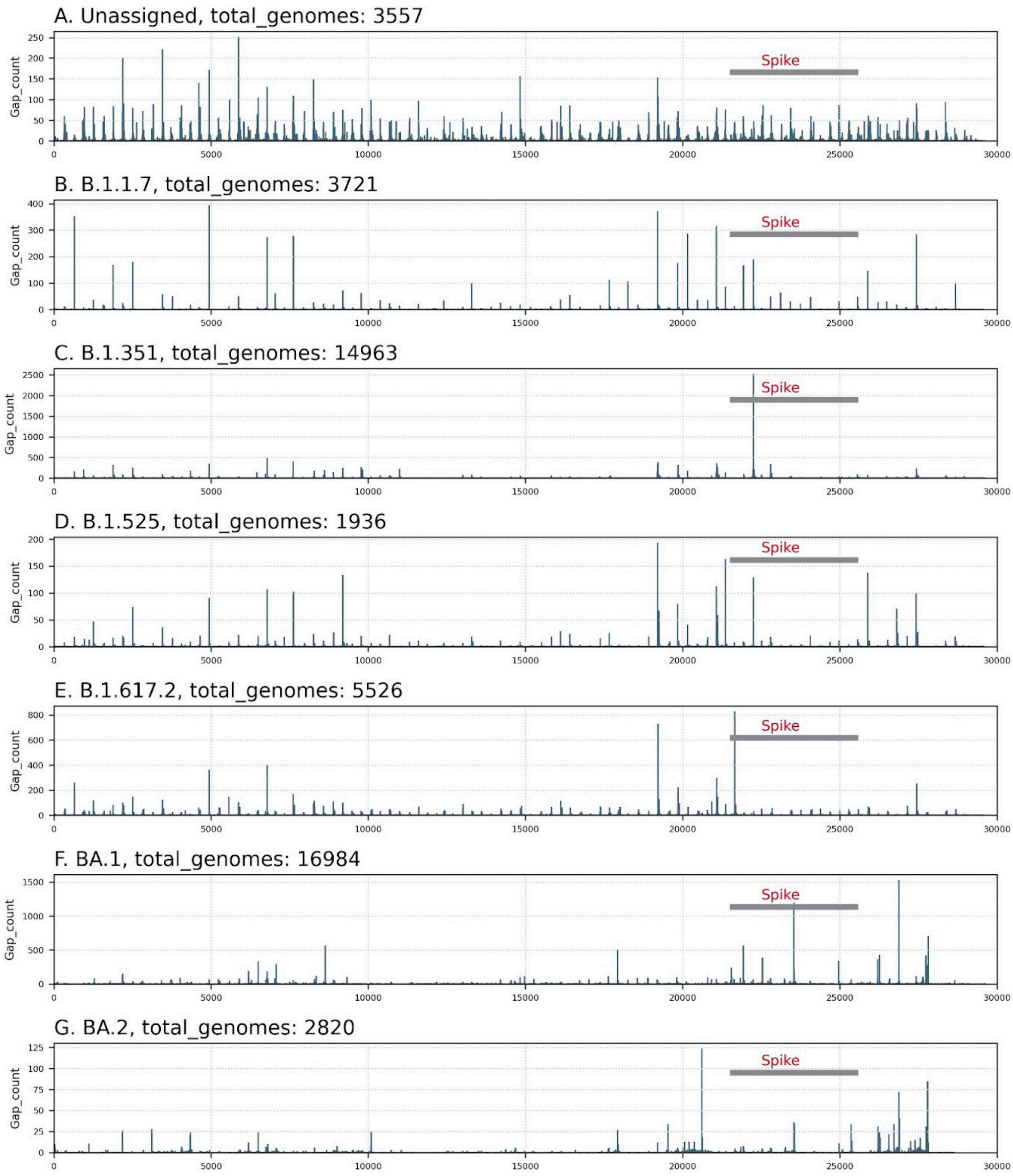

Supplementary Figure S9. Gapped positions in genomes by lineage. The positions of 200 nt N gaps in the 6 major SARS-CoV-2 lineages (plus unassigned entries) were plotted to document lineage-specific patterns in the genome entries. For each lineage (or unassigned entries) the number of 200nt N motifs was plotted by position. The lineage and the total number of genomes classified in the lineage are indicated above each panel. The position of the Spike coding region is indicated with a grey bar. A) Unassigned, B) B.1.1.7 (Alpha), C) B.1.351 (Beta), D) B.1.525 (Eta), E) B.1.617.2 (Delta), F) BA.1 (Omicron), G) BA.2 (Omicron).

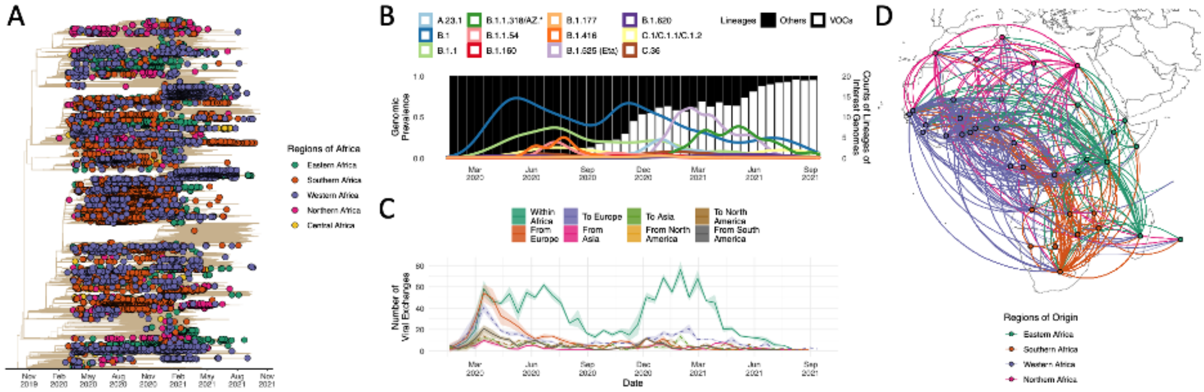

Supplementary Figure S10: Phylogenetic inference of non-VOC lineages in Africa. A) Maximum-Likelihood timetrees of non-VOC genomes in Africa from the beginning of the pandemic till March 2022 against a global reference with African genomes denoted by tippoint circles coloured by regions of Africa. B) Genomic prevalence of non-VOC (black) vs VOC (white) lineages in Africa overlaid by frequency progressions of some lineages of interest in Africa. C) *Inferred viral dissemination patterns of non-VOC lineages to, from and within the Africa continent from the beginning of the pandemic to October 2021. Introductions and viral transitions within Africa are shown in solid lines and exports from Africa are shown in dotted lines and these are coloured by continent. The shaded areas around the lines represent uncertainty of this analysis from ten replicates. D) Dissemination patterns of the non-VOC lineages within Africa, from inferred ancestral state reconstructions, annotated and coloured by region in Africa. The countries of origin of viral exchange routes are also shown with dots and the curves go from country of origin to destination country in an anti-clockwise direction.*

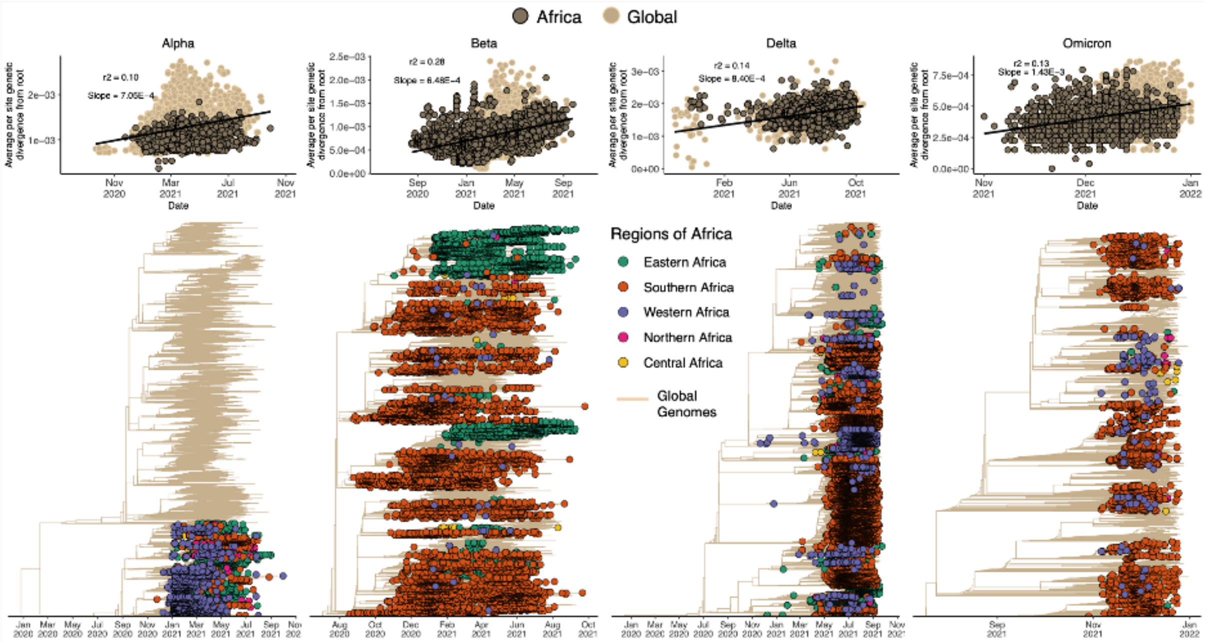

Supplementary Figure S11: Phylogenetic inference of VOCs in Africa using Africa-focused sampling. Top - Molecular clock evolution. Bottom - VOC-specific Maximum-Likelihood timetrees with African genomes denoted by tippoints coloured by regions of Africa.

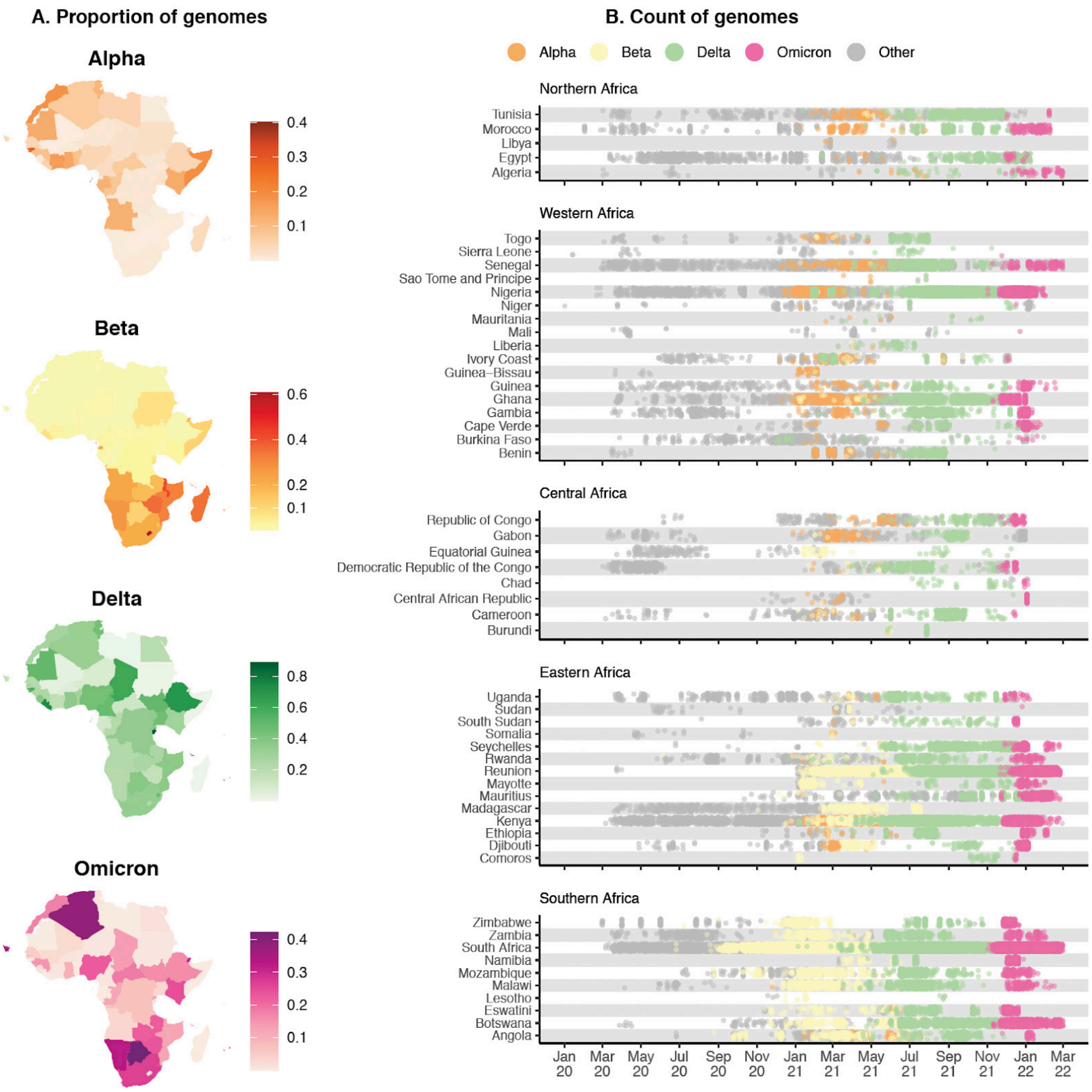

Supplementary Figure S12: Spatial and temporal circulation of four variants of concern (VOC) across Africa. A) The proportion of genomes of each VOC (of all genomes produced) sequenced per country. B) Scatterplot displaying the total number of genomes sequenced per VOC per country from January 2020 to March 2022. Alpha and Beta variants circulated within Africa at similar times in the epidemic however, a greater number of Alpha genomes were sequenced in Northern and Western African countries whereas Beta was more prevalent in Eastern and Southern African countries. Certain Central and Eastern African countries, such as Cameroon and Uganda, display a similar level of co-circulation of both variants. Thereafter, Delta and then Omicron, mostly dominated the landscape in isolation.

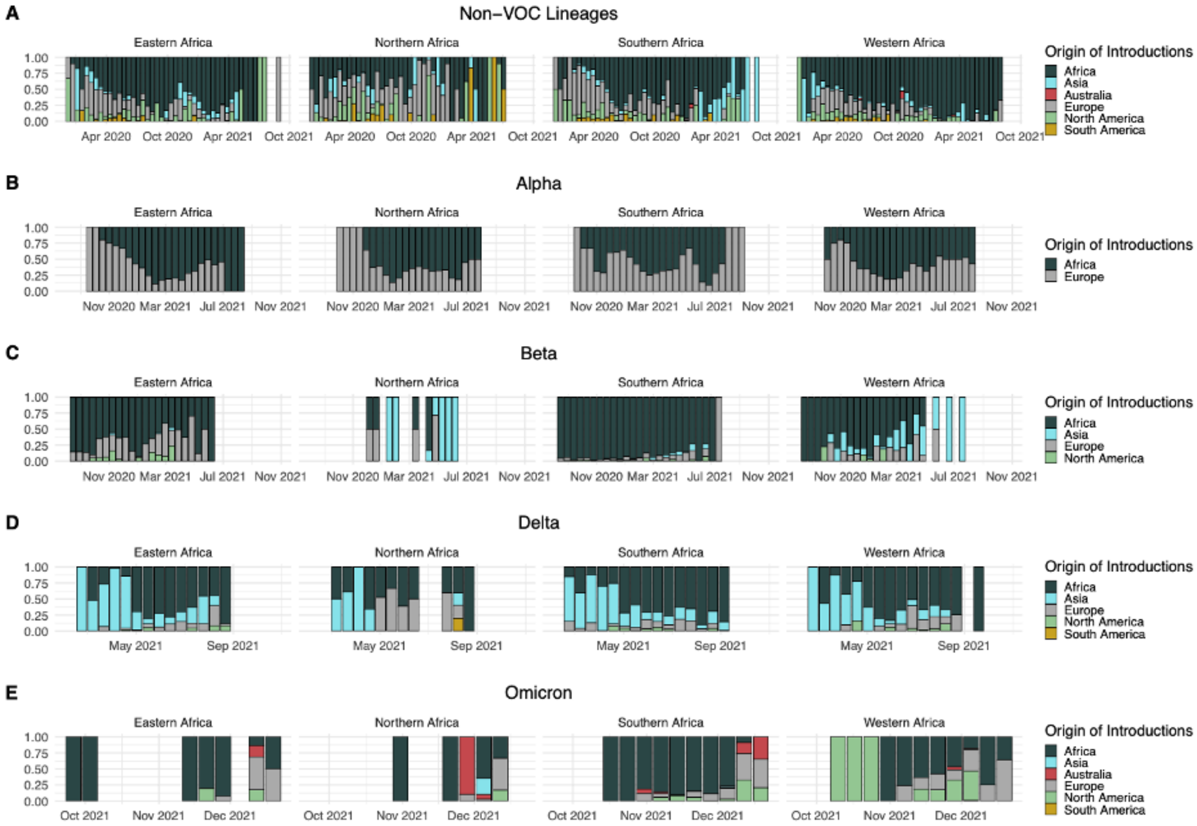

Supplementary Figure S13: Patterns of viral importations into different regions of Africa under an African-focused sampling strategy. Proportions of introductions into East, Northern, southern and West Africa attributed to specified origins for A) non-VOC lineages, B) Alpha VOC, C) Beta VOC, D) Delta VOC, and E) Omicron VOC during the relevant time periods are shown.

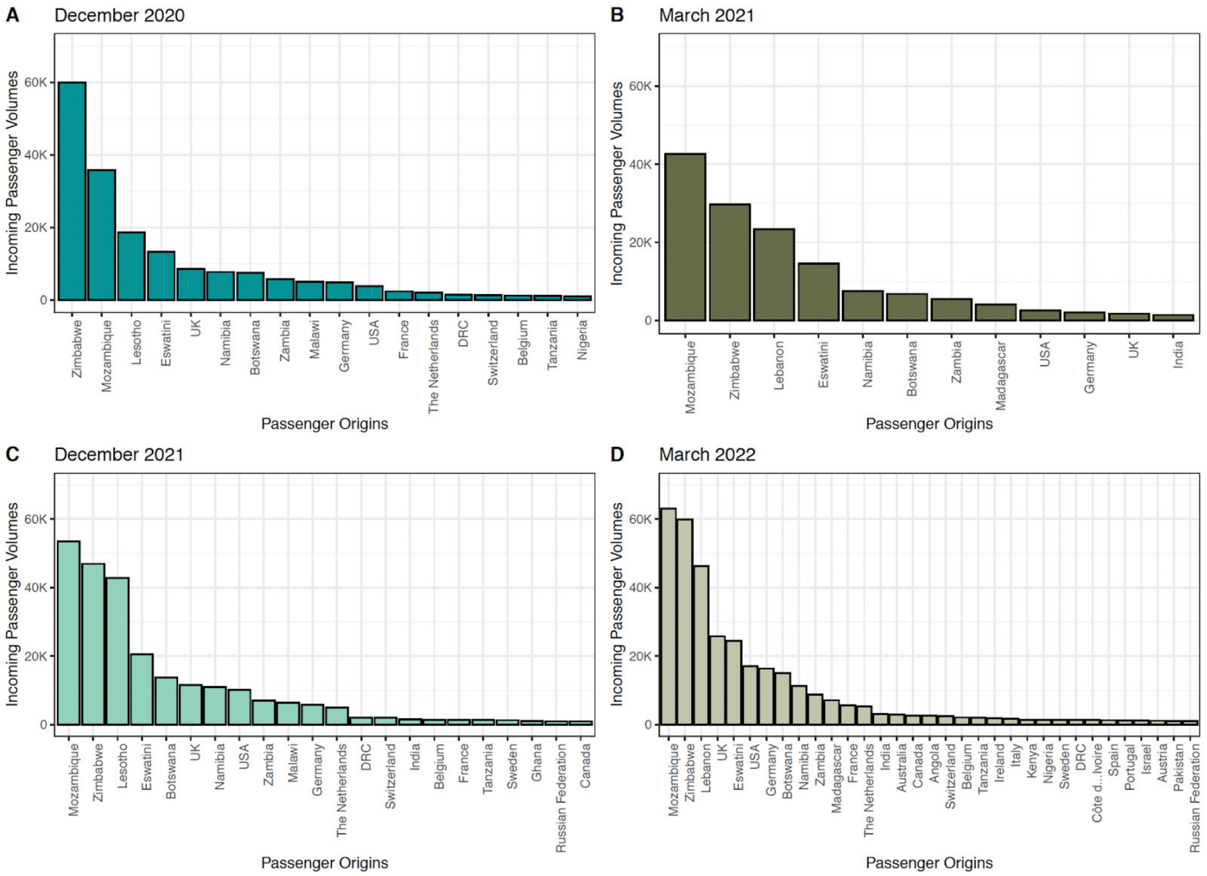

Supplementary Figure S14: Statistics of incoming passenger volume into South Africa by country of origin. Periods shown are December 2020 (A), March 2021 (B), December 2021 (C), and March 2022 (D). Data is obtained from Statistics South Africa Statistical Release P0351 - Tourism and Migration.

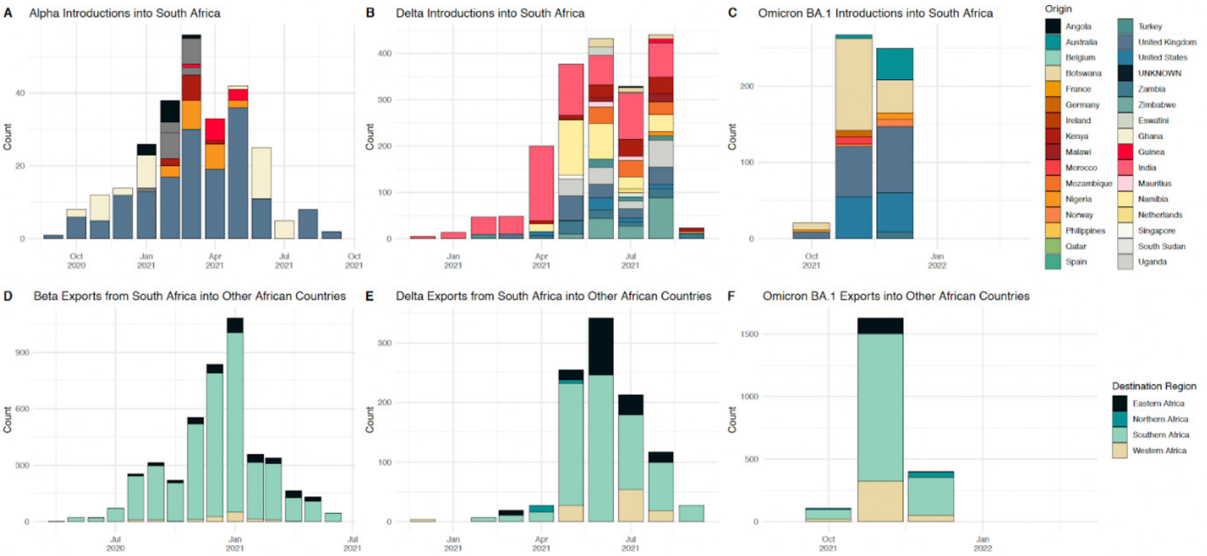

Supplementary Figure S15: Inferred introductions into and out of South Africa specified by country of inferred origin. (A) Alpha introductions into South Africa from Africa-focused phylogeography, (B) Delta introductions into South Africa from global case-sensitive phylogeography, (C) Omicron BA.1 introductions into South Africa from global case-sensitive

phylogeography, (D-E) Beta, Delta and Omicron BA.1 exports from South Africa into regions of Africa from Africa-focused phylogeography.

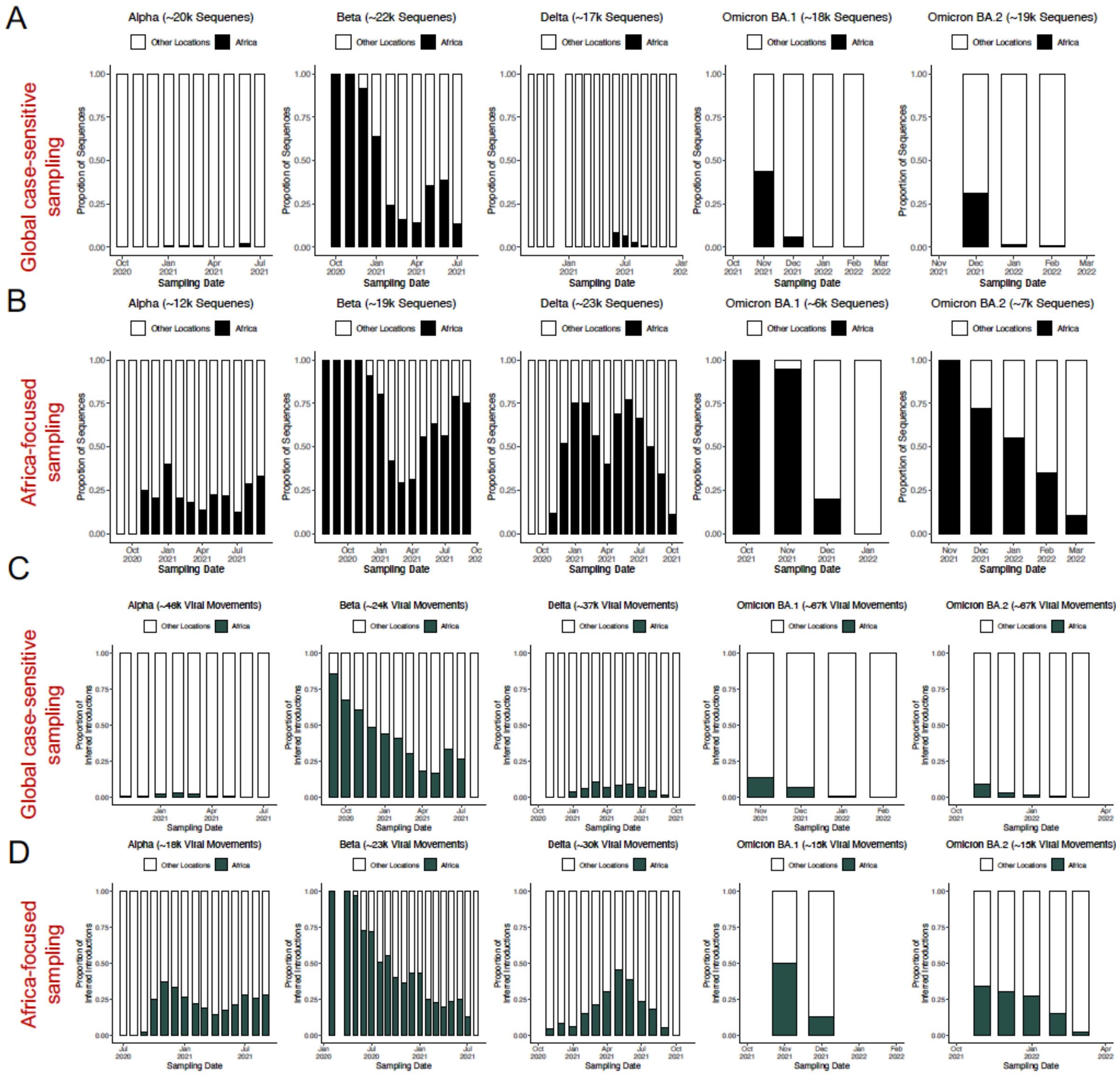

Supplementary Figure S16: Sensitivity analysis for phylogeography from ancestral state reconstruction. (A-B) Sampling proportions of African sequences in two strategies. (C-D) Proportion of inferred introductions into Africa in corresponding two strategies

Supplementary Table S1: NextStrain builds of the five major geographical regions in Africa. Each build focuses on a specific region within Africa and includes sequences from outside the region and the continent to place the regional sequences into context of the global pandemic. All the data used in the builds are publicly available on GISAID and are maintained and updated by the Africa CDC in collaboration with the NextStrain team on a weekly basis.

Region	Regional Builds
Central African Region	https://nextstrain.org/groups/africa-cdc/ncov/central-africa
Eastern African Region	https://nextstrain.org/groups/africa-cdc/ncov/eastern-africa
Northern African Region	https://nextstrain.org/groups/africa-cdc/ncov/northern-africa

Southern African Region	https://nextstrain.org/groups/africa-cdc/ncov/southern-africa
Western African Region	https://nextstrain.org/groups/africa-cdc/ncov/western-africa

Supplementary Table S2: Individual country specific NextStrain builds from the African continent. Each build focuses on a specific country within Africa and includes sequences from the rest of the world in order to place the country's sequences into context of the global pandemic. All the data used in the builds are publicly available on GISAID and are maintained and updated by the Africa CDC in collaboration with the NextStrain team on a weekly basis.

Region	Country	Build
Central African Region	Burundi	https://nextstrain.org/groups/africa-cdc/ncov/burundi
	Cameroon	https://nextstrain.org/groups/africa-cdc/ncov/cameroon
	Central African Republic	https://nextstrain.org/groups/africa-cdc/ncov/central-african-republic
	Chad	https://nextstrain.org/groups/africa-cdc/ncov/chad
	Republic of Congo	https://nextstrain.org/groups/africa-cdc/ncov/republic-of-the-congo
	Democratic Republic of Congo	https://nextstrain.org/groups/africa-cdc/ncov/democratic-republic-of-the-congo
	Equatorial Guinea	https://nextstrain.org/groups/africa-cdc/ncov/equatorial-guinea
	Gabon	https://nextstrain.org/groups/africa-cdc/ncov/gabon
Eastern African Region	Comoros	https://nextstrain.org/groups/africa-cdc/ncov/comoros
	Djibouti	https://nextstrain.org/groups/africa-cdc/ncov/djibouti
	Ethiopia	https://nextstrain.org/groups/africa-cdc/ncov/ethiopia
	Kenya	https://nextstrain.org/groups/africa-cdc/ncov/kenya
	Madagascar	https://nextstrain.org/groups/africa-cdc/ncov/madagascar

	Mauritius	https://nextstrain.org/groups/africa-cdc/ncov/mauritius
	Rwanda	https://nextstrain.org/groups/africa-cdc/ncov/rwanda
	Seychelles	https://nextstrain.org/groups/africa-cdc/ncov/seychelles
	Somalia	https://nextstrain.org/groups/africa-cdc/ncov/somalia
	South Sudan	https://nextstrain.org/groups/africa-cdc/ncov/south-sudan
	Sudan	https://nextstrain.org/groups/africa-cdc/ncov/sudan
	Uganda	https://nextstrain.org/groups/africa-cdc/ncov/uganda
Northern African Region	Algeria	https://nextstrain.org/groups/africa-cdc/ncov/algeria
	Egypt	https://nextstrain.org/groups/africa-cdc/ncov/egypt
	Libya	https://nextstrain.org/groups/africa-cdc/ncov/libya
	Morocco	https://nextstrain.org/groups/africa-cdc/ncov/morocco
	Tunisia	https://nextstrain.org/groups/africa-cdc/ncov/tunisia
Southern African Region	Angola	https://nextstrain.org/groups/africa-cdc/ncov/angola
	Botswana	https://nextstrain.org/groups/africa-cdc/ncov/botswana
	Eswatini	https://nextstrain.org/groups/africa-cdc/ncov/eswatini
	Lesotho	https://nextstrain.org/groups/africa-cdc/ncov/lesotho
	Malawi	https://nextstrain.org/groups/africa-cdc/ncov/malawi
	Mozambique	https://nextstrain.org/groups/africa-cdc/ncov/mozambique
	Namibia	https://nextstrain.org/groups/africa-cdc/ncov/namibia

	South Africa	https://nextstrain.org/groups/africa-cdc/ncov/south-africa
	Zambia	https://nextstrain.org/groups/africa-cdc/ncov/zambia
	Zimbabwe	https://nextstrain.org/groups/africa-cdc/ncov/zimbabwe
Western African Region	Benin	https://nextstrain.org/groups/africa-cdc/ncov/benin
	Burkina Faso	https://nextstrain.org/groups/africa-cdc/ncov/burkina-faso
	Cabo Verde	https://nextstrain.org/groups/africa-cdc/ncov/cabo-verde
	Côte d'Ivoire	https://nextstrain.org/groups/africa-cdc/ncov/cote-divoire
	Gambia	https://nextstrain.org/groups/africa-cdc/ncov/gambia
	Ghana	https://nextstrain.org/groups/africa-cdc/ncov/ghana
	Guinea	https://nextstrain.org/groups/africa-cdc/ncov/guinea
	Guinea-Bissau	https://nextstrain.org/groups/africa-cdc/ncov/guinea-bissau
	Liberia	https://nextstrain.org/groups/africa-cdc/ncov/liberia
	Mali	https://nextstrain.org/groups/africa-cdc/ncov/mali
	Niger	https://nextstrain.org/groups/africa-cdc/ncov/niger
	Nigeria	https://nextstrain.org/groups/africa-cdc/ncov/nigeria
	Senegal	https://nextstrain.org/groups/africa-cdc/ncov/senegal
	Sierra Leone	https://nextstrain.org/groups/africa-cdc/ncov/sierra-leone
	Togo	https://nextstrain.org/groups/africa-cdc/ncov/togo

Supplementary Table S3 (excel file): Sequencing and epidemiological reporting survey results. S3.1) Aggregate results of the survey with responses from 25 countries across the continent. S3.2) frequency of epidemiological reporting (i.e. of new cases and deaths) in the

different countries. S3.3) Sequencing strategies employed by different countries as the pandemic progressed. S3.4) The proportion of sequences from the major administrative regions of each country (i.e. provinces, districts or regions). The table can also be found at the github repository (https://github.com/CERI-KRISP/SARS-CoV-2-epidemic-in-Africa).

Supplementary Table S4 (excel file): GISAID acknowledgment table. The table can also be found at the github repository (https://github.com/CERI-KRISP/SARS-CoV-2-epidemic-in-Africa).

References and Notes

1. Q. Li, X. Guan, P. Wu, X. Wang, L. Zhou, Y. Tong, R. Ren, K. S. M. Leung, E. H. Y. Lau, J. Y. Wong, X. Xing, N. Xiang, Y. Wu, C. Li, Q. Chen, D. Li, T. Liu, J. Zhao, M. Liu, W. Tu, C. Chen, L. Jin, R. Yang, Q. Wang, S. Zhou, R. Wang, H. Liu, Y. Luo, Y. Liu, G. Shao, H. Li, Z. Tao, Y. Yang, Z. Deng, B. Liu, Z. Ma, Y. Zhang, G. Shi, T. T. Y. Lam, J. T. Wu, G. F. Gao, B. J. Cowling, B. Yang, G. M. Leung, Z. Feng, Early transmission dynamics in Wuhan, China, of novel coronavirus-infected pneumonia. *N. Engl. J. Med.* **382**, 1199–1207 (2020). doi:10.1056/NEJMoa2001316 Medline

2. J. Hasell, E. Mathieu, D. Beltekian, B. Macdonald, C. Giattino, E. Ortiz-Ospina, M. Roser, H. Ritchie, A cross-country database of COVID-19 testing. *Sci. Data* **7**, 345 (2020). doi:10.1038/s41597-020-00688-8 Medline

3. R. Viana, S. Moyo, D. G. Amoako, H. Tegally, C. Scheepers, C. L. Althaus, U. J. Anyaneji, P. A. Bester, M. F. Boni, M. Chand, W. T. Choga, R. Colquhoun, M. Davids, K. Deforche, D. Doolabh, L. du Plessis, S. Engelbrecht, J. Everatt, J. Giandhari, M. Giovanetti, D. Hardie, V. Hill, N. Y. Hsiao, A. Iranzadeh, A. Ismail, C. Joseph, R. Joseph, L. Koopile, S. L. Kosakovsky Pond, M. U. G. Kraemer, L. Kuate-Lere, O. Laguda-Akingba, O. Lesetedi-Mafoko, R. J. Lessells, S. Lockman, A. G. Lucaci, A. Maharaj, B. Mahlangu, T. Maponga, K. Mahlakwane, Z. Makatini, G. Marais, D. Maruapula, K. Masupu, M. Matshaba, S. Mayaphi, N. Mbhele, M. B. Mbulawa, A. Mendes, K. Mlisana, A. Mnguni, T. Mohale, M. Moir, K. Moruisi, M. Mosepele, G. Motsatsi, M. S. Motswaledi, T. Mphoyakgosi, N. Msomi, P. N. Mwangi, Y. Naidoo, N. Ntuli, M. Nyaga, L. Olubayo, S. Pillay, B. Radibe, Y. Ramphal, U. Ramphal, J. E. San, L. Scott, R. Shapiro, L. Singh, P. Smith-Lawrence, W. Stevens, A. Strydom, K. Subramoney, N. Tebeila, D. Tshiabuila, J. Tsui, S. van Wyk, S. Weaver, C. K. Wibmer, E. Wilkinson, N. Wolter, A. E. Zarebski, B. Zuze, D. Goedhals, W. Preiser, F. Treurnicht, M. Venter, C. Williamson, O. G. Pybus, J. Bhiman, A. Glass, D. P. Martin, A. Rambaut, S. Gaseitsiwe, A. von Gottberg, T. de Oliveira, Rapid epidemic expansion of the SARS-CoV-2 Omicron variant in southern Africa. *Nature* **603**, 679–686 (2022). doi:10.1038/s41586-022-04411-y Medline

4. H. Tegally, E. Wilkinson, M. Giovanetti, A. Iranzadeh, V. Fonseca, J. Giandhari, D. Doolabh, S. Pillay, E. J. San, N. Msomi, K. Mlisana, A. von Gottberg, S. Walaza, M. Allam, A. Ismail, T. Mohale, A. J. Glass, S. Engelbrecht, G. Van Zyl, W. Preiser, F. Petruccione, A. Sigal, D. Hardie, G. Marais, N. Y. Hsiao, S. Korsman, M. A. Davies, L. Tyers, I. Mudau, D. York, C. Maslo, D. Goedhals, S. Abrahams, O. Laguda-Akingba, A. Alisoltani-Dehkordi, A. Godzik, C. K. Wibmer, B. T. Sewell, J. Lourenço, L. C. J. Alcantara, S. L. Kosakovsky Pond, S. Weaver, D. Martin, R. J. Lessells, J. N. Bhiman, C. Williamson, T. de Oliveira, Detection of a SARS-CoV-2 variant of concern in South Africa. *Nature* **592**, 438–443 (2021). doi:10.1038/s41586-021-03402-9 Medline

5. D. P. Martin, S. Weaver, H. Tegally, J. E. San, S. D. Shank, E. Wilkinson, A. G. Lucaci, J. Giandhari, S. Naidoo, Y. Pillay, L. Singh, R. J. Lessells, R. K. Gupta, J. O. Wertheim, A. Nekturenko, B. Murrell, G. W. Harkins, P. Lemey, O. A. MacLean, D. L. Robertson, T. de Oliveira, S. L. Kosakovsky Pond, NGS-SA, COVID-19 Genomics UK (COG-UK), The emergence and ongoing convergent evolution of the SARS-CoV-2 N501Y lineages. *Cell* **184**, 5189–5200.e7 (2021). doi:10.1016/j.cell.2021.09.003 Medline

6. F. Campbell, B. Archer, H. Laurenson-Schafer, Y. Jinnai, F. Konings, N. Batra, B. Pavlin, K. Vandemaele, M. D. Van Kerkhove, T. Jombart, O. Morgan, O. le Polain de Waroux, Increased transmissibility and global spread of SARS-CoV-2 variants of concern as at June 2021. *Euro Surveill.* **26**, (2021). doi:10.2807/1560-7917.ES.2021.26.24.2100509 Medline

7. B. Korber, W. M. Fischer, S. Gnanakaran, H. Yoon, J. Theiler, W. Abfalterer, N. Hengartner, E. E. Giorgi, T. Bhattacharya, B. Foley, K. M. Hastie, M. D. Parker, D. G. Partridge, C. M. Evans, T. M. Freeman, T. I. de Silva, C. McDanal, L. G. Perez, H. Tang, A. Moon-Walker, S. P. Whelan, C. C. LaBranche, E. O. Saphire, D. C. Montefiori, A. Angyal, R. L. Brown, L. Carrilero, L. R. Green, D. C. Groves, K. J. Johnson, A. J. Keeley, B. B. Lindsey, P. J. Parsons, M. Raza, S. Rowland-Jones, N. Smith, R. M. Tucker, D. Wang, M. D. Wyles, Sheffield COVID-19 Genomics Group, Tracking changes in SARS-CoV-2 spike: Evidence that D614G increases infectivity of the COVID-19 virus. *Cell* **182**, 812–827.e19 (2020). doi:10.1016/j.cell.2020.06.043 Medline

8. E. Hacisuleyman, C. Hale, Y. Saito, N. E. Blachere, M. Bergh, E. G. Conlon, D. J. Schaefer-Babajew, J. DaSilva, F. Muecksch, C. Gaebler, R. Lifton, M. C. Nussenzweig, T. Hatziioannou, P. D. Bieniasz, R. B. Darnell, Vaccine breakthrough infections with SARS-CoV-2 variants. *N. Engl. J. Med.* **384**, 2212–2218 (2021). doi:10.1056/NEJMoa2105000 Medline

9. D. Planas, D. Veyer, A. Baidaliuk, I. Staropoli, F. Guivel-Benhassine, M. M. Rajah, C. Planchais, F. Porrot, N. Robillard, J. Puech, M. Prot, F. Gallais, P. Gantner, A. Velay, J. Le Guen, N. Kassis-Chikhani, D. Edriss, L. Belec, A. Seve, L. Courtellemont, H. Péré, L. Hocqueloux, S. Fafi-Kremer, T. Prazuck, H. Mouquet, T. Bruel, E. Simon-Lorière, F. A. Rey, O. Schwartz, Reduced sensitivity of SARS-CoV-2 variant Delta to antibody neutralization. *Nature* **596**, 276–280 (2021). doi:10.1038/s41586-021-03777-9 Medline

10. S. Yue, Z. Li, Y. Lin, Y. Yang, M. Yuan, Z. Pan, L. Hu, L. Gao, J. Zhou, J. Tang, Y. Wang, Q. Tian, Y. Hao, J. Wang, Q. Huang, L. Xu, B. Zhu, P. Liu, K. Deng, L. Wang, L. Ye, X. Chen, Sensitivity of SARS-CoV-2 variants to neutralization by convalescent sera and a VH3-30 monoclonal antibody. *Front. Immunol.* **12**, 751584 (2021). doi:10.3389/fimmu.2021.751584 Medline

11. S. Cele, I. Gazy, L. Jackson, S.-H. Hwa, H. Tegally, G. Lustig, J. Giandhari, S. Pillay, E. Wilkinson, Y. Naidoo, F. Karim, Y. Ganga, K. Khan, M. Bernstein, A. B. Balazs, B. I. Gosnell, W. Hanekom, M. S. Moosa, R. J. Lessells, T. de Oliveira, A. Sigal, Network for Genomic Surveillance in South Africa, COMMIT-KZN Team, Escape of SARS-CoV-2 501Y.V2 from neutralization by convalescent plasma. *Nature* **593**, 142–146 (2021). doi:10.1038/s41586-021-03471-w Medline

12. B. Meng, S. A. Kemp, G. Papa, R. Datir, I. A. T. M. Ferreira, S. Marelli, W. T. Harvey, S. Lytras, A. Mohamed, G. Gallo, N. Thakur, D. A. Collier, P. Mlcochova, L. M. Duncan, A. M. Carabelli, J. C. Kenyon, A. M. Lever, A. De Marco, C. Saliba, K. Culap, E. Cameroni, N. J. Matheson, L. Piccoli, D. Corti, L. C. James, D. L. Robertson, D. Bailey, R. K. Gupta, COVID-19 Genomics UK (COG-UK) Consortium, Recurrent emergence of SARS-CoV-2 spike deletion H69/V70 and its role in the Alpha variant B.1.1.7. *Cell Rep.* **35**, 109292 (2021). doi:10.1016/j.celrep.2021.109292 Medline

13. P. Mlcochova, S. A. Kemp, M. S. Dhar, G. Papa, B. Meng, I. A. T. M. Ferreira, R. Datir, D. A. Collier, A. Albecka, S. Singh, R. Pandey, J. Brown, J. Zhou, N. Goonawardane, S. Mishra, C. Whittaker, T. Mellan, R. Marwal, M. Datta, S. Sengupta, K. Ponnusamy, V. S. Radhakrishnan, A. Abdullahi, O. Charles, P. Chattopadhyay, P. Devi, D. Caputo, T. Peacock, C. Wattal, N. Goel, A. Satwik, R. Vaishya, M. Agarwal, A. Mavousian, J. H. Lee, J. Bassi, C. Silacci-Fegni, C. Saliba, D. Pinto, T. Irie, I. Yoshida, W. L. Hamilton, K. Sato, S. Bhatt, S. Flaxman, L. C. James, D. Corti, L. Piccoli, W. S. Barclay, P. Rakshit, A. Agrawal, R. K. Gupta, Indian SARS-CoV-2 Genomics Consortium (INSACOG), Genotype to Phenotype Japan (G2P-Japan) Consortium, CITIID-NIHR BioResource COVID-19 Collaboration, SARS-CoV-2 B.1.617.2 Delta variant replication and immune evasion. *Nature* **599**, 114–119 (2021). doi:10.1038/s41586-021-03944-y Medline

14. N. R. Faria, T. A. Mellan, C. Whittaker, I. M. Claro, D. D. S. Candido, S. Mishra, M. A. E. Crispim, F. C. S. Sales, I. Hawryluk, J. T. McCrone, R. J. G. Hulswit, L. A. M. Franco, M. S. Ramundo, J. G. de Jesus, P. S. Andrade, T. M. Coletti, G. M. Ferreira, C. A. M. Silva, E. R. Manuli, R. H. M. Pereira, P. S. Peixoto, M. U. G. Kraemer, N. Gaburo Jr., C. D. C. Camilo, H. Hoeltgebaum, W. M. Souza, E. C. Rocha, L. M. de Souza, M. C. de Pinho, L. J. T. Araujo, F. S. V. Malta, A. B. de Lima, J. D. P. Silva, D. A. G. Zauli, A. C. S. Ferreira, R. P. Schnekenberg, D. J. Laydon, P. G. T. Walker, H. M. Schlüter, A. L. P. Dos Santos, M. S. Vidal, V. S. Del Caro, R. M. F. Filho, H. M. Dos Santos, R. S. Aguiar, J. L. Proença-Modena, B. Nelson, J. A. Hay, M. Monod, X. Miscouridou, H. Coupland, R. Sonabend, M. Vollmer, A. Gandy, C. A. Prete Jr., V. H. Nascimento, M. A. Suchard, T. A. Bowden, S. L. K. Pond, C.-H. Wu, O. Ratmann, N. M. Ferguson, C. Dye, N. J. Loman, P. Lemey, A. Rambaut, N. A. Fraiji, M. D. P. S. S. Carvalho, O. G. Pybus, S. Flaxman, S. Bhatt, E. C. Sabino, Genomics and epidemiology of the P.1 SARS-CoV-2 lineage in Manaus, Brazil. *Science* **372**, 815–821 (2021). doi:10.1126/science.abh2644 Medline

15. E. Wilkinson, M. Giovanetti, H. Tegally, J. E. San, R. Lessells, D. Cuadros, D. P. Martin, D. A. Rasmussen, A. N. Zekri, A. K. Sangare, A.-S. Ouedraogo, A. K. Sesay, A. Priscilla, A.-S. Kemi, A. M. Olubusuyi, A. O. O. Oluwapelumi, A. Hammami, A. A. Amuri, A. Sayed, A. E. O. Ouma, A. Elargoubi, N. A. Ajayi, A. F. Victoria, A. Kazeem, A. George, A. J. Trotter, A. A. Yahaya, A. K. Keita, A. Diallo, A. Kone, A. Souissi, A. Chtourou, A. V. Gutierrez, A. J. Page, A. Vinze, A. Iranzadeh, A. Lambisia, A. Ismail, A. Rosemary, A. Sylverken, A. Femi, A. Ibrahimi, B. Marycelin, B. S. Oderinde, B. Bolajoko, B. Dhaala, B. L. Herring, B.-M. Njanpop-Lafourcade, B. Kleinhans, B. McInnis, B. Tegomoh, C. Brook, C. B. Pratt, C. Scheepers, C. G. Akoua-Koffi, C. N. Agoti, C. Peyrefitte, C. Daubenberger, C. M. Morang'a, D. J. Nokes, D. G. Amoako, D. L. Bugembe, D. Park, D. Baker, D. Doolabh, D. Ssemwanga, D. Tshiabuila, D. Bassirou, D. S. Y. Amuzu, D. Goedhals, D. O. Omuoyo, D. Maruapula, E. Foster-Nyarko, E. K. Lusamaki, E. Simulundu, E. M. Ong'era, E. N. Ngabana, E. Shumba, E. El Fahime, E. Lokilo, E. Mukantwari, E. Philomena, E. Belarbi, E. Simon-Loriere, E. A. Anoh, F. Leendertz, F. Ajili, F. O. Enoch, F. Wasfi, F. Abdelmoula, F. S. Mosha, F. T. Takawira, F. Derrar, F. Bouzid, F. Onikepe, F. Adeola, F. M. Muyembe, F. Tanser, F. A. Dratibi, G. K. Mbunsu, G. Thilliez, G. L. Kay, G. Githinji, G. van Zyl, G. A. Awandare, G. Schubert, G. P. Maphalala, H. C. Ranaivoson, H. Lemriss, H. Anise, H. Abe, H. H. Karray, H. Nansumba, H. A. Elgahzaly, H. Gumbo, I. Smeti, I. B. Ayed, I. Odia, I. B. Ben Boubaker, I. Gaaloul, I. Gazy, I. Mudau, I. Ssewanyana, I. Konstantinus, J. B. Lekana-Douk, J.-C. C. Makangara, J. M. Tamfum, J.-M. Heraud, J. G. Shaffer, J.

Giandhari, J. Li, J. Yasuda, J. Q. Mends, J. Kiconco, J. M. Morobe, J. O. Gyapong, J. C. Okolie, J. T. Kayiwa, J. A. Edwards, J. Gyamfi, J. Farah, J. Nakaseegu, J. M. Ngoi, J. Namulondo, J. C. Andeko, J. J. Lutwama, J. O'Grady, K. Siddle, K. T. Adeyemi, K. A. Tumedi, K. M. Said, K. Hae-Young, K. O. Duedu, L. Belyamani, L. Fki-Berrajah, L. Singh, L. O. Martins, L. Tyers, M. Ramuth, M. Mastouri, M. Aouni, M. El Hefnawi, M. I. Matsheka, M. Kebabonye, M. Diop, M. Turki, M. Paye, M. M. Nyaga, M. Mareka, M.-M. Damaris, M. W. Mburu, M. Mpina, M. Nwando, M. Owusu, M. R. Wiley, M. T. Youtchou, M. O. Ayekaba, M. Abouelhoda, M. G. Seadawy, M. K. Khalifa, M. Sekhele, M. Ouadghiri, M. M. Diagne, M. Mwenda, M. Allam, M. V. T. Phan, N. Abid, N. Touil, N. Rujeni, N. Kharrat, N. Ismael, N. Dia, N. Mabunda, N. Y. Hsiao, N. B. Silochi, N. Nsenga, N. Gumede, N. Mulder, N. Ndodo, N. H. Razanajatovo, N. Iguosadolo, O. Judith, O. C. Kingsley, O. Sylvanus, O. Peter, O. Femi, O. Idowu, O. Testimony, O. E. Chukwuma, O. E. Ogah, C. K. Onwuamah, O. Cyril, O. Faye, O. Tomori, P. Ondoa, P. Combe, P. Semanda, P. E. Oluniyi, P. Arnaldo, P. K. Quashie, P. Dussart, P. A. Bester, P. K. Mbala, R. Ayivor-Djanie, R. Njouom, R. O. Phillips, R. Gorman, R. A. Kingsley, R. A. A. Carr, S. El Kabbaj, S. Gargouri, S. Masmoudi, S. Sankhe, S. B. Lawal, S. Kassim, S. Trabelsi, S. Metha, S. Kammoun, S. Lemriss, S. H. A. Agwa, S. Calvignac-Spencer, S. F. Schaffner, S. Doumbia, S. M. Mandanda, S. Aryeetey, S. S. Ahmed, S. Elhamoumi, S. Andriamandimby, S. Tope, S. Lekana-Douki, S. Prosolek, S. Ouangraoua, S. A. Mundeke, S. Rudder, S. Panji, S. Pillay, S. Engelbrecht, S. Nabadda, S. Behillil, S. L. Budiaki, S. van der Werf, T. Mashe, T. Aanniz, T. Mohale, T. Le-Viet, T. Schindler, U. J. Anyaneji, U. Chinedu, U. Ramphal, U. Jessica, U. George, V. Fonseca, V. Enouf, V. Gorova, W. H. Roshdy, W. K. Ampofo, W. Preiser, W. T. Choga, Y. Bediako, Y. Naidoo, Y. Butera, Z. R. de Laurent, A. A. Sall, A. Rebai, A. von Gottberg, B. Kouriba, C. Williamson, D. J. Bridges, I. Chikwe, J. N. Bhiman, M. Mine, M. Cotten, S. Moyo, S. Gaseitsiwe, N. Saasa, P. C. Sabeti, P. Kaleebu, Y. K. Tebeje, S. K. Tessema, C. Happi, J. Nkengasong, T. de Oliveira, A year of genomic surveillance reveals how the SARS-CoV-2 pandemic unfolded in Africa. *Science* **374**, 423–431 (2021). doi:10.1126/science.abj4336 Medline

16. Y. Shu, J. McCauley, GISAID: Global initiative on sharing all influenza data - from vision to reality. *Euro Surveill.* **22**, 30494 (2017). doi:10.2807/1560-7917.ES.2017.22.13.30494 Medline

17. C. Kuiken, B. Korber, R. W. Shafer, HIV sequence databases. *AIDS Rev.* **5**, 52–61 (2003). Medline

18. D. L. Bugembe, J. Kayiwa, M. V. T. Phan, P. Tushabe, S. Balinandi, B. Dhaala, J. Lexow, H. Mwebesa, J. Aceng, H. Kyobe, D. Ssemwanga, J. Lutwama, P. Kaleebu, M. Cotten, Main routes of entry and genomic diversity of SARS-CoV-2, Uganda. *Emerg. Infect. Dis.* **26**, 2411–2415 (2020). doi:10.3201/eid2610.202575 Medline

19. T. Mashe, F. T. Takawira, L. de Oliveira Martins, M. Gudza-Mugabe, J. Chirenda, M. Munyanyi, B. V. Chaibva, A. Tarupiwa, H. Gumbo, A. Juru, C. Nyagupe, V. Ruhanya, I. Phiri, P. Manangazira, A. Goredema, S. Danda, I. Chabata, J. Jonga, R. Munharira, K. Masunda, I. Mukeredzi, D. Mangwanya, A. Trotter, T. Le Viet, S. Rudder, G. Kay, D. Baker, G. Thilliez, A. V. Gutierrez, J. O'Grady, M. Hove, S. Mutapuri-Zinyowera, A. J. Page, R. A. Kingsley, G. Mhlanga, COVID-19 Genomics UK Consortium, SARS-CoV-2 Research Group, Genomic epidemiology and the role of international and regional travel in the SARS-CoV-2 epidemic in Zimbabwe: A

retrospective study of routinely collected surveillance data. *Lancet Glob. Health* **9**, e1658–e1666 (2021). doi:10.1016/S2214-109X(21)00434-4 Medline

20. A. Chouikha, W. Fares, A. Laamari, S. Haddad-Boubaker, Z. Belaiba, K. Ghedira, W. Kammoun Rebai, K. Ayouni, M. Khedhiri, S. Ben Halima, H. Krichen, H. Touzi, I. Ben Dhifallah, F. Z. Guerfali, C. Atri, S. Azouz, O. Khamessi, M. Ardhaoui, M. Safer, N. Ben Alaya, I. Guizani, R. Kefi, M. Gdoura, H. Triki, Molecular epidemiology of SARS-CoV-2 in Tunisia (North Africa) through several successive waves of COVID-19. *Viruses* **14**, 624 (2022). doi:10.3390/v14030624 Medline

21. F. Ntoumi, C. C. Mfoutou Mapanguy, A. Tomazatos, S. R. Pallerla, L. T. K. Linh, N. Casadei, A. Angelov, M. Sonnabend, S. Peter, P. G. Kremsner, T. P. Velavan, Genomic surveillance of SARS-CoV-2 in the Republic of Congo. *Int. J. Infect. Dis.* **105**, 735–738 (2021). doi:10.1016/j.ijid.2021.03.036 Medline

22. Y. Butera, E. Mukantwari, M. Artesi, J. D'Arc Umuringa, Á. N. O'Toole, V. Hill, S. Rooke, S. L. Hong, S. Dellicour, O. Majyambere, S. Bontems, B. Boujemla, J. Quick, P. C. Resende, N. Loman, E. Umumararungu, A. Kabanda, M. M. Murindahabi, P. Tuyisenge, M. Gashegu, N. Rujeni, Genomic sequencing of SARS-CoV-2 in Rwanda: Evolution and regional dynamics. medRxiv 2021.04.02.21254839 [Preprint] (2021); https://doi.org/10.1101/2021.04.02.21254839.

23. C. N Agoti, G. Githinji, K. S Mohammed, A. W Lambisia, Z. R de Laurent, M. W Mburu, E. M Ong'era, J. M Morobe, E. Otieno, H. Abdou Azali, K. Said Abdallah, A. Diarra, A. Ahmed Yahaya, P. Borus, N. Gumede Moeletsi, D. Fred Athanasius, B. Tsofa, P. Bejon, D. James Nokes, L. Isabella Ochola-Oyier, Detection of SARS-CoV-2 variant 501Y.V2 in Comoros Islands in January 2021. *Wellcome Open Res.* **6**, 192 (2021). doi:10.12688/wellcomeopenres.16889.1 Medline

24. J. M. Morobe, B. Pool, L. Marie, D. Didon, A. W. Lambisia, T. Makori, K. S. Mohammed, Z. R. de Laurent, L. Ndwiga, M. W. Mburu, E. Moraa, N. Murunga, J. Musyoki, J. Mwacharo, L. Nyamako, D. Riako, P. Ephnatus, F. Gambo, J. Naimani, J. Namulondo, S. Z. Tembo, E. Ogendi, T. Balde, F. A. Dratibi, A. A. Yahaya, N. Gumede, R. A. Achilla, P. K. Borus, D. W. Wanjohi, S. K. Tessema, J. Mwangangi, P. Bejon, D. J. Nokes, L. I. Ochola-Oyier, G. Githinji, L. Biscornet, C. N. Agoti, Genomic Epidemiology of SARS-CoV-2 in Seychelles, 2020-2021. *Viruses* **14**, 1318 (2022). doi:10.3390/v14061318 Medline

25. C. M. Morang'a, J. M. Ngoi, J. Gyamfi, D. S. Y. Amuzu, B. D. Nuertey, P. M. Soglo, V. Appiah, I. A. Asante, P. Owusu-Oduro, S. Armoo, D. Adu-Gyasi, N. Amoako, J. Oliver-Commey, M. Owusu, A. Sylverken, E. D. Fenteng, V. V. M'cormack, F. Tei-Maya, E. B. Quansah, R. Ayivor-Djanie, E. K. Amoako, I. T. Ogbe, B. K. Yemi, I. Osei-Wusu, D. N. A. Mettle, S. Saiid, K. Tapela, F. Dzabeng, V. Magnussen, J. Quaye, P. C. Opurum, R. A. Carr, P. T. Ababio, A. K. Abass, S. K. Akoriyea, E. Amoako, F. Kumi-Ansah, O. D. Boakye, D. K. Mibut, T. Odoom, L. Ofori-Boadu, E. Allegye-Cudjoe, S. Dassah, V. Asoala, K. P. Asante, R. O. Phillips, M. Y. Osei-Atweneboana, J. O. Gyapong, P. Kuma-Aboagye, W. K. Ampofo, K. O. Duedu, N. T. Ndam, Y. Bediako, P. K. Quashie, L. N. Amenga-Etego, G. A. Awandare, Genetic diversity of SARS-CoV-2 infections in Ghana from 2020-2021. *Nat. Commun.* **13**, 2494 (2022). doi:10.1038/s41467-022-30219-5 Medline

26. C. N. Agoti, L. I. Ochola-Oyier, S. Dellicour, K. S. Mohammed, A. W. Lambisia, Z. R. de Laurent, J. M. Morobe, M. W. Mburu, D. O. Omuoyo, E. M. Ongera, L. Ndwiga, E. Maitha, B. Kitole, T. Suleiman, M. Mwakinangu, J. K. Nyambu, J. Otieno, B.

Salim, J. Musyoki, N. Murunga, E. Otieno, J. N. Kiiru, K. Kasera, P. Amoth, M. Mwangangi, R. Aman, S. Kinyanjui, G. Warimwe, M. Phan, A. Agweyu, M. Cotten, E. Barasa, B. Tsofa, D. J. Nokes, P. Bejon, G. Githinji, Transmission networks of SARS-CoV-2 in Coastal Kenya during the first two waves: A retrospective genomic study. *eLife* **11**, e71703 (2022). doi:10.7554/eLife.71703 Medline

27. S. P. C. Brand, J. Ojal, R. Aziza, V. Were, E. A. Okiro, I. K. Kombe, C. Mburu, M. Ogero, A. Agweyu, G. M. Warimwe, J. Nyagwange, H. Karanja, J. N. Gitonga, D. Mugo, S. Uyoga, I. M. O. Adetifa, J. A. G. Scott, E. Otieno, N. Murunga, M. Otiende, L. I. Ochola-Oyier, C. N. Agoti, G. Githinji, K. Kasera, P. Amoth, M. Mwangangi, R. Aman, W. Ng'ang'a, B. Tsofa, P. Bejon, M. J. Keeling, D. J. Nokes, E. Barasa, COVID-19 transmission dynamics underlying epidemic waves in Kenya. *Science* **374**, 989–994 (2021). doi:10.1126/science.abk0414 Medline

28. G. Githinji, Z. R. de Laurent, K. S. Mohammed, D. O. Omuoyo, P. M. Macharia, J. M. Morobe, E. Otieno, S. M. Kinyanjui, A. Agweyu, E. Maitha, B. Kitole, T. Suleiman, M. Mwakinangu, J. Nyambu, J. Otieno, B. Salim, K. Kasera, J. Kiiru, R. Aman, E. Barasa, G. Warimwe, P. Bejon, B. Tsofa, L. I. Ochola-Oyier, D. J. Nokes, C. N. Agoti, Tracking the introduction and spread of SARS-CoV-2 in coastal Kenya. *Nat. Commun.* **12**, 4809 (2021). doi:10.1038/s41467-021-25137-x Medline

29. H. Tegally, E. Wilkinson, R. J. Lessells, J. Giandhari, S. Pillay, N. Msomi, K. Mlisana, J. N. Bhiman, A. von Gottberg, S. Walaza, V. Fonseca, M. Allam, A. Ismail, A. J. Glass, S. Engelbrecht, G. Van Zyl, W. Preiser, C. Williamson, F. Petruccione, A. Sigal, I. Gazy, D. Hardie, N. Y. Hsiao, D. Martin, D. York, D. Goedhals, E. J. San, M. Giovanetti, J. Lourenço, L. C. J. Alcantara, T. de Oliveira, Sixteen novel lineages of SARS-CoV-2 in South Africa. *Nat. Med.* **27**, 440–446 (2021). doi:10.1038/s41591-021-01255-3 Medline

30. W. H. Roshdy, M. K. Khalifa, J. E. San, H. Tegally, E. Wilkinson, S. Showky, D. P. Martin, M. Moir, A. Naguib, N. Elguindy, M. R. Gomaa, M. Fahim, H. A. Elsood, A. A. Mohsen, R. Galal, M. Hassany, R. J. Lessells, A. A. Al Karmalawy, R. E. L. Shesheny, A. M. Kandeil, T. de Oliveira, SARS-CoV-2 Genetic diversity and lineage dynamics of in Egypt. medRxiv 2022.01.05.22268646 [Preprint] (2022); https://doi.org/10.1101/2022.01.05.22268646.

31. C. Scheepers, J. Everatt, D. G. Amoako, H. Tegally, C. K. Wibmer, A. Mnguni, A. Ismail, B. Mahlangu, B. E. Lambson, D. P. Martin, E. Wilkinson, J. E. San, J. Giandhari, N. Manamela, N. Ntuli, P. Kgagudi, S. Cele, S. I. Richardson, S. Pillay, T. Mohale, U. Ramphal, Y. Naidoo, Z. T. Khumalo, G. Kwatra, G. Gray, L.-G. Bekker, S. A. Madhi, V. Baillie, W. C. Van Voorhis, F. K. Treurnicht, M. Venter, K. Mlisana, N. Wolter, A. Sigal, C. Williamson, N. Y. Hsiao, N. Msomi, T. Maponga, W. Preiser, Z. Makatini, R. Lessells, P. L. Moore, T. de Oliveira, A. von Gottberg, J. N. Bhiman, Emergence and phenotypic characterization of the global SARS-CoV-2 C.1.2 lineage. *Nat. Commun.* **13**, 1976 (2022). doi:10.1038/s41467-022-29579-9 Medline

32. G. Dudas, S. L. Hong, B. I. Potter, S. Calvignac-Spencer, F. S. Niatou-Singa, T. B. Tombolomako, T. Fuh-Neba, U. Vickos, M. Ulrich, F. H. Leendertz, K. Khan, C. Huber, A. Watts, I. Olendraitė, J. Snijder, K. N. Wijnant, A. M. J. J. Bonvin, P. Martres, S. Behillil, A. Ayouba, M. F. Maidadi, D. M. Djomsi, C. Godwe, C. Butel, A. Šimaitis, M. Gabrielaitė, M. Katėnaitė, R. Norvilas, L. Raugaitė, G. W. Koyaweda, J. K. Kandou, R. Jonikas, I. Nasvytienė, Ž. Žemeckienė, D. Gečys, K. Tamušauskaitė, M. Norkienė, E. Vasiliūnaitė, D. Žiogienė, A. Timinskas, M. Šukys, M. Šarauskas, G.

Alzbutas, A. A. Aziza, E. K. Lusamaki, J. M. Cigolo, F. M. Mawete, E. L. Lofiko, P. M. Kingebeni, J. M. Tamfum, M. R. D. Belizaire, R. G. Essomba, M. C. O. Assoumou, A. B. Mboringong, A. B. Dieng, D. Juozapaitė, S. Hosch, J. Obama, M. O. Ayekaba, D. Naumovas, A. Pautienius, C. D. Rafaï, A. Vitkauskienė, R. Ugenskienė, A. Gedvilaitė, D. Čereškevičius, V. Lesauskaitė, L. Žemaitis, L. Griškevičius, G. Baele, Emergence and spread of SARS-CoV-2 lineage B.1.620 with variant of concern-like mutations and deletions. *Nat. Commun.* **12**, 5769 (2021). doi:10.1038/s41467-021-26055-8 Medline

33. D. L. Bugembe, M. V. T. Phan, I. Ssewanyana, P. Semanda, H. Nansumba, B. Dhaala, S. Nabadda, Á. N. O'Toole, A. Rambaut, P. Kaleebu, M. Cotten, Emergence and spread of a SARS-CoV-2 lineage A variant (A.23.1) with altered spike protein in Uganda. *Nat. Microbiol.* **6**, 1094–1101 (2021). doi:10.1038/s41564-021-00933-9 Medline

34. H. Tegally, M. Moir, J. Everatt, M. Giovanetti, C. Scheepers, E. Wilkinson, K. Subramoney, Z. Makatini, S. Moyo, D. G. Amoako, C. Baxter, C. L. Althaus, U. J. Anyaneji, D. Kekana, R. Viana, J. Giandhari, R. J. Lessells, T. Maponga, D. Maruapula, W. Choga, M. Matshaba, M. B. Mbulawa, N. Msomi, Y. Naidoo, S. Pillay, T. J. Sanko, J. E. San, L. Scott, L. Singh, N. A. Magini, P. Smith-Lawrence, W. Stevens, G. Dor, D. Tshiabuila, N. Wolter, W. Preiser, F. K. Treurnicht, M. Venter, G. Chiloane, C. McIntyre, A. O'Toole, C. Ruis, T. P. Peacock, C. Roemer, S. L. Kosakovsky Pond, C. Williamson, O. G. Pybus, J. N. Bhiman, A. Glass, D. P. Martin, B. Jackson, A. Rambaut, O. Laguda-Akingba, S. Gaseitsiwe, A. von Gottberg, T. de Oliveira, NGS-SA Consortium, Emergence of SARS-CoV-2 Omicron lineages BA.4 and BA.5 in South Africa. *Nat. Med.* (2022). doi:10.1038/s41591-022-01911-2 Medline

35. S. A. Madhi, G. Kwatra, J. E. Myers, W. Jassat, N. Dhar, C. K. Mukendi, A. J. Nana, L. Blumberg, R. Welch, N. Ngorima-Mabhena, P. C. Mutevedzi, Population immunity and Covid-19 severity with omicron variant in South Africa. *N. Engl. J. Med.* **386**, 1314–1326 (2022). doi:10.1056/NEJMoa2119658 Medline

36. N. Wolter, W. Jassat, S. Walaza, R. Welch, H. Moultrie, M. Groome, D. G. Amoako, J. Everatt, J. N. Bhiman, C. Scheepers, N. Tebeila, N. Chiwandire, M. du Plessis, N. Govender, A. Ismail, A. Glass, K. Mlisana, W. Stevens, F. K. Treurnicht, Z. Makatini, N. Y. Hsiao, R. Parboosing, J. Wadula, H. Hussey, M. A. Davies, A. Boulle, A. von Gottberg, C. Cohen, Early assessment of the clinical severity of the SARS-CoV-2 omicron variant in South Africa: A data linkage study. *Lancet* **399**, 437–446 (2022). doi:10.1016/S0140-6736(22)00017-4 Medline

37. H. C. Lewis, H. Ware, M. Whelan, L. Subissi, Z. Li, X. Ma, A. Nardone, M. Valenciano, B. Cheng, K. Noel, C. Cao, M. Yanes-Lane, B. L. Herring, A. Talisuna, N. Ngoy, T. Balde, D. Clifton, M. D. Van Kerkhove, D. Buckeridge, N. Bobrovitz, J. Okeibunor, R. K. Arora, I. Bergeri, UNITY Studies Collaborator Group, SARS-CoV-2 infection in Africa: A systematic review and meta-analysis of standardised seroprevalence studies, from January 2020 to December 2021. *BMJ Glob. Health* **7**, e008793 (2022). doi:10.1136/bmjgh-2022-008793 Medline

38. R. M. Barber, R. J. D. Sorensen, D. M. Pigott, C. Bisignano, A. Carter, J. O. Amlag, J. K. Collins, C. Abbafati, C. Adolph, A. Allorant, A. Y. Aravkin, B. L. Bang-Jensen, E. Castro, S. Chakrabarti, R. M. Cogen, E. Combs, H. Comfort, K. Cooperrider, X. Dai, F. Daoud, A. Deen, L. Earl, M. Erickson, S. B. Ewald, A. J. Ferrari, A. D. Flaxman, J. J. Frostad, N. Fullman, J. R. Giles, G. Guo, J. He, M. Helak, E. N. Hulland, B. M.

Huntley, A. Lazzar-Atwood, K. E. LeGrand, S. S. Lim, A. Lindstrom, E. Linebarger, R. Lozano, B. Magistro, D. C. Malta, J. Månsson, A. M. Mantilla Herrera, A. H. Mokdad, L. Monasta, M. Naghavi, S. Nomura, C. M. Odell, L. T. Olana, S. M. Ostroff, M. Pasovic, S. A. Pease, R. C. Reiner Jr., G. Reinke, A. L. P. Ribeiro, D. F. Santomauro, A. Sholokhov, E. E. Spurlock, R. Syailendrawati, R. Topor-Madry, A. T. Vo, T. Vos, R. Walcott, A. Walker, K. E. Wiens, C. S. Wiysonge, N. A. Worku, P. Zheng, S. I. Hay, E. Gakidou, C. J. L. Murray, COVID-19 Cumulative Infection Collaborators, Estimating global, regional, and national daily and cumulative infections with SARS-CoV-2 through Nov 14, 2021: A statistical analysis. *Lancet* **399**, 2351–2380 (2022). doi:10.1016/S0140-6736(22)00484-6 Medline

39. J. Quick, nCoV-2019 sequencing protocol v3 (LoCost) (2020).

40. J. R. Tyson, P. James, D. Stoddart, N. Sparks, A. Wickenhagen, G. Hall, J. H. Choi, H. Lapointe, K. Kamelian, A. D. Smith, N. Prystajecky, I. Goodfellow, S. J. Wilson, R. Harrigan, T. P. Snutch, N. J. Loman, J. Quick, Improvements to the ARTIC multiplex PCR method for SARS-CoV-2 genome sequencing using nanopore. bioRxiv 2020.09.04.283077 [Preprint] (2020); https://doi.org/10.1101/2020.09.04.283077.

41. M. Cotten, D. Lule Bugembe, P. Kaleebu, M. V T Phan, Alternate primers for whole-genome SARS-CoV-2 sequencing. *Virus Evol.* **7**, veab006 (2021). doi:10.1093/ve/veab006 Medline

42. H. Tegally, M. Ramuth, D. Amoaka, C. Scheepers, E. Wilkinson, M. Giovanetti, R. J. Lessells, J. Giandhari, A. Ismail, D. Martin, E. J. San, M. Crawford, R. S. Daniels, R. Harvey, S. Bahadoor, J. Sonoo, M. Timol, L. Veerapa-Mangroo, A. von Gottberg, J. Bhiman, T. de Oliveira, S. Manraj, A novel and expanding SARS-CoV-2 variant, B.1.1.318, dominates infections in Mauritius. medRxiv 2021.06.16.21259017 [Preprint] (2021); https://doi.org/10.1101/2021.06.16.21259017.

43. A. N. Zekri, A. A. Bahnasy, M. M. Hafez, Z. K. Hassan, O. S. Ahmed, H. K. Soliman, E. R. El-Sisi, M. H. S. E. Dine, M. S. Solimane, L. S. A. Latife, M. G. Seadawy, A. S. Elsafty, M. Abouelhoda, Characterization of the SARS-CoV-2 genomes in Egypt in first and second waves of infection. *Sci. Rep.* **11**, 21632 (2021). doi:10.1038/s41598-021-99014-4 Medline

44. C. Nasimiyu, D. Matoke-Muhia, G. K. Rono, E. Osoro, D. O. Obado, J. M. Mwangi, N. Mwikwabe, K. Thiongâ O, J. Dawa, I. Ngere, J. Gachohi, S. Kariuki, E. Amukoye, M. Mureithi, P. Ngere, P. Amoth, I. Were, L. Makayotto, V. Nene, E. O. Abworo, M. K. Njenga, S. N. Seifert, S. O. Oyola, Imported SARS-COV-2 variants of concern drove spread of infections across Kenya during the second year of the pandemic. *COVID* **2**, 586–598 (2022). doi:10.3390/covid2050044

45. M. U. G. Kraemer, V. Hill, C. Ruis, S. Dellicour, S. Bajaj, J. T. McCrone, G. Baele, K. V. Parag, A. L. Battle, B. Gutierrez, B. Jackson, R. Colquhoun, Á. O'Toole, B. Klein, A. Vespignani, E. Volz, N. R. Faria, D. M. Aanensen, N. J. Loman, L. du Plessis, S. Cauchemez, A. Rambaut, S. V. Scarpino, O. G. Pybus, COVID-19 Genomics UK (COG-UK) Consortium, Spatiotemporal invasion dynamics of SARS-CoV-2 lineage B.1.1.7 emergence. *Science* **373**, 889–895 (2021). doi:10.1126/science.abj0113 Medline

46. S. J. Lycett, J. Hughes, M. P. McHugh, A. da Silva Filipe, R. Dewar, L. Lu, T. Doherty, A. Shepherd, R. Inward, G. Rossi, D. Balaz, R. R. Kao, S. Rooke, S. Cotton, M. D. Gallagher, C. B. Lopez, Á. O'Toole, E. Scher, V. Hill, J. T. McCrone, R. M. Colquhoun, B. Jackson, T. C. Williams, K. A. Williamson, N. Johnson, K. Smollett,

D. Mair, S. Carmichael, L. Tong, J. Nichols, K. Brunker, J. G. Shepherd, K. Li, E. Aranday-Cortes, Y. A. Parr, A. Broos, K. Nomikou, S. E. McDonald, M. Niebel, P. Asamaphan, I. Starinskij, N. Jesudason, R. Shah, V. B. Sreenu, T. Stanton, S. Shaaban, A. MacLean, M. Woolhouse, R. Gunson, K. Templeton, E. C. Thomson, A. Rambaut, M. T. G. Holden, D. L. Robertson, COVID-19 Genomics UK (COG-UK) Consortium, Epidemic waves of COVID-19 in Scotland: a genomic perspective on the impact of the introduction and relaxation of lockdown on SARS-CoV-2. medRxiv 2021.01.08.20248677 [Preprint] (2021); https://doi.org/10.1101/2021.01.08.20248677.

47. P. W. G. Mallon, F. Crispie, G. Gonzalez, W. Tinago, A. A. Garcia Leon, M. McCabe, E. de Barra, O. Yousif, J. S. Lambert, C. J. Walsh, J. G. Kenny, E. Feeney, M. Carr, P. Doran, P. D. Cotter, Whole-genome sequencing of SARS-CoV-2 in the Republic of Ireland during waves 1 and 2 of the pandemic. medRxiv 2021.02.09.21251402 [Preprint] (2021); https://doi.org/10.1101/2021.02.09.21251402.

48. H. Tegally, E. Wilkinson, C. L. Althaus, M. Giovanetti, J. E. San, J. Giandhari, S. Pillay, Y. Naidoo, U. Ramphal, N. Msomi, K. Mlisana, D. G. Amoako, J. Everatt, T. Mohale, A. Nguni, B. Mahlangu, N. Ntuli, Z. T. Khumalo, Z. Makatini, N. Wolter, C. Scheepers, A. Ismail, D. Doolabh, R. Joseph, A. Strydom, A. Mendes, M. Davis, S. H. Mayaphi, Y. Ramphal, A. Maharaj, W. A. Karim, D. Tshiabuila, U. J. Anyaneji, L. Singh, S. Engelbrecht, V. Fonseca, K. Marais, S. Korsman, D. Hardie, N. Hsiao, T. Maponga, G. van Zyl, G. Marais, A. Iranzadeh, D. Martin, L. C. J. Alcantara, P. A. Bester, M. M. Nyaga, K. Subramoney, F. K. Treurnicht, M. Venter, D. Goedhals, W. Preiser, J. N. Bhiman, A. Gottberg, C. Williamson, R. J. Lessells, T. de Oliveira, Rapid replacement of the Beta variant by the Delta variant in South Africa. medRxiv 2021.09.23.21264018 [Preprint] (2021); https://doi.org/10.1101/2021.09.23.21264018.

49. S. Chang, E. Pierson, P. W. Koh, J. Gerardin, B. Redbird, D. Grusky, J. Leskovec, Mobility network models of COVID-19 explain inequities and inform reopening. *Nature* **589**, 82–87 (2021). doi:10.1038/s41586-020-2923-3 Medline

50. M. Chinazzi, J. T. Davis, M. Ajelli, C. Gioannini, M. Litvinova, S. Merler, A. Pastore Y Piontti, K. Mu, L. Rossi, K. Sun, C. Viboud, X. Xiong, H. Yu, M. E. Halloran, I. M. Longini Jr., A. Vespignani, The effect of travel restrictions on the spread of the 2019 novel coronavirus (COVID-19) outbreak. *Science* **368**, 395–400 (2020). doi:10.1126/science.aba9757 Medline

51. M. U. G. Kraemer, C.-H. Yang, B. Gutierrez, C.-H. Wu, B. Klein, D. M. Pigott, L. du Plessis, N. R. Faria, R. Li, W. P. Hanage, J. S. Brownstein, M. Layan, A. Vespignani, H. Tian, C. Dye, O. G. Pybus, S. V. Scarpino, Open COVID-19 Data Working Group, The effect of human mobility and control measures on the COVID-19 epidemic in China. *Science* **368**, 493–497 (2020). doi:10.1126/science.abb4218 Medline

52. P. Nouvellet, S. Bhatia, A. Cori, K. E. C. Ainslie, M. Baguelin, S. Bhatt, A. Boonyasiri, N. F. Brazeau, L. Cattarino, L. V. Cooper, H. Coupland, Z. M. Cucunuba, G. Cuomo-Dannenburg, A. Dighe, B. A. Djaafara, I. Dorigatti, O. D. Eales, S. L. van Elsland, F. F. Nascimento, R. G. FitzJohn, K. A. M. Gaythorpe, L. Geidelberg, W. D. Green, A. Hamlet, K. Hauck, W. Hinsley, N. Imai, B. Jeffrey, E. Knock, D. J. Laydon, J. A. Lees, T. Mangal, T. A. Mellan, G. Nedjati-Gilani, K. V. Parag, M. Pons-Salort, M. Ragonnet-Cronin, S. Riley, H. J. T. Unwin, R. Verity, M. A. C. Vollmer, E. Volz, P. G. T. Walker, C. E. Walters, H. Wang, O. J. Watson, C. Whittaker, L. K. Whittles, X.

Xi, N. M. Ferguson, C. A. Donnelly, Reduction in mobility and COVID-19 transmission. *Nat. Commun.* **12**, 1090 (2021). doi:10.1038/s41467-021-21358-2 Medline

53. C. Xiong, S. Hu, M. Yang, W. Luo, L. Zhang, Mobile device data reveal the dynamics in a positive relationship between human mobility and COVID-19 infections. *Proc. Natl. Acad. Sci. U.S.A.* **117**, 27087–27089 (2020). doi:10.1073/pnas.2010836117 Medline

54. S. Pillay, J. Giandhari, H. Tegally, E. Wilkinson, B. Chimukangara, R. Lessells, Y. Moosa, S. Mattison, I. Gazy, M. Fish, L. Singh, K. S. Khanyile, J. E. San, V. Fonseca, M. Giovanetti, L. C. Alcantara Jr., T. de Oliveira, Whole genome sequencing of SARS-CoV-2: Adapting Illumina protocols for quick and accurate outbreak investigation during a pandemic. *Genes* **11**, 949 (2020). doi:10.3390/genes11080949 Medline

55. L. Singh, J. E. San, H. Tegally, P. M. Brzoska, U. J. Anyaneji, E. Wilkinson, L. Clark, J. Giandhari, S. Pillay, R. J. Lessells, D. P. Martin, M. Furtado, A. M. Kiran, T. de Oliveira, Targeted Sanger sequencing to recover key mutations in SARS-CoV-2 variant genome assemblies produced by next-generation sequencing. *Microb. Genom.* **8**, (2022). doi:10.1099/mgen.0.000774 Medline

56. A. J. Page, A. E. Mather, T. Le-Viet, E. J. Meader, N.-F. Alikhan, G. L. Kay, L. de Oliveira Martins, A. Aydin, D. J. Baker, A. J. Trotter, S. Rudder, A. P. Tedim, A. Kolyva, R. Stanley, M. Yasir, M. Diaz, W. Potter, C. Stuart, L. Meadows, A. Bell, A. V. Gutierrez, N. M. Thomson, E. M. Adriaenssens, T. Swingler, R. A. J. Gilroy, L. Griffith, D. K. Sethi, D. Aggarwal, C. S. Brown, R. K. Davidson, R. A. Kingsley, L. Bedford, L. J. Coupland, I. G. Charles, N. Elumogo, J. Wain, R. Prakash, M. A. Webber, S. J. L. Smith, M. Chand, S. Dervisevic, J. O'Grady, The Covid-Genomics Uk Cog-Uk Consortium, Large-scale sequencing of SARS-CoV-2 genomes from one region allows detailed epidemiology and enables local outbreak management. *Microb. Genom.* **7**, (2021). doi:10.1099/mgen.0.000589 Medline

57. N. De Maio, C. Walker, R. Borges, L. Weilguny, G. Slodkowicz, N. Goldman, Issues with SARS-CoV-2 sequencing data, Virological.org (2020); https://virological.org/t/issues-with-sars-cov-2-sequencing-data/473.

58. N. E. Freed, M. Vlková, M. B. Faisal, O. K. Silander, Rapid and inexpensive whole-genome sequencing of SARS-CoV-2 using 1200 bp tiled amplicons and Oxford Nanopore Rapid Barcoding. *Biol. Methods Protoc.* **5**, bpaa014 (2020). doi:10.1093/biomethods/bpaa014 Medline

59. J.-S. Eden, R. Rockett, I. Carter, H. Rahman, J. de Ligt, J. Hadfield, M. Storey, X. Ren, R. Tulloch, K. Basile, J. Wells, R. Byun, N. Gilroy, M. V. O'Sullivan, V. Sintchenko, S. C. Chen, S. Maddocks, T. C. Sorrell, E. C. Holmes, D. E. Dwyer, J. Kok, 2019-nCoV Study Group, An emergent clade of SARS-CoV-2 linked to returned travellers from Iran. *Virus Evol.* **6**, veaa027 (2020). doi:10.1093/ve/veaa027 Medline

60. A. S. Gonzalez-Reiche, M. M. Hernandez, M. J. Sullivan, B. Ciferri, H. Alshammary, A. Obla, S. Fabre, G. Kleiner, J. Polanco, Z. Khan, B. Alburquerque, A. van de Guchte, J. Dutta, N. Francoeur, B. S. Melo, I. Oussenko, G. Deikus, J. Soto, S. H. Sridhar, Y.-C. Wang, K. Twyman, A. Kasarskis, D. R. Altman, M. Smith, R. Sebra, J. Aberg, F. Krammer, A. García-Sastre, M. Luksza, G. Patel, A. Paniz-Mondolfi, M. Gitman, E. M. Sordillo, V. Simon, H. van Bakel, Introductions and early spread of SARS-CoV-2 in the New York City area. *Science* **369**, 297–301 (2020). doi:10.1126/science.abc1917 Medline

61. K. Itokawa, T. Sekizuka, M. Hashino, R. Tanaka, M. Kuroda, Disentangling primer interactions improves SARS-CoV-2 genome sequencing by multiplex tiling PCR. *PLOS ONE* **15**, e0239403 (2020). doi:10.1371/journal.pone.0239403 Medline

62. A. X. Han, A. Toporowski, J. A. Sacks, M. Perkins, S. Briand, M. van Kerkhove, E. Hannay, S. Carmona, B. Rodriguez, E. Parker, B. E. Nichols, C. A. Russell, Low testing rates limit the ability of genomic surveillance programs to monitor SARS-CoV-2 variants: a mathematical modelling study. medRxiv 2022.05.20.22275319 [Preprint] (2022); https://doi.org/10.1101/2022.05.20.22275319.

63. H. Wang, K. R. Paulson, S. A. Pease, S. Watson, H. Comfort, P. Zheng, A. Y. Aravkin, C. Bisignano, R. M. Barber, T. Alam, J. E. Fuller, E. A. May, D. P. Jones, M. E. Frisch, C. Abbafati, C. Adolph, A. Allorant, J. O. Amlag, B. Bang-Jensen, G. J. Bertolacci, S. S. Bloom, A. Carter, E. Castro, S. Chakrabarti, J. Chattopadhyay, R. M. Cogen, J. K. Collins, K. Cooperrider, X. Dai, W. J. Dangel, F. Daoud, C. Dapper, A. Deen, B. B. Duncan, M. Erickson, S. B. Ewald, T. Fedosseeva, A. J. Ferrari, J. J. Frostad, N. Fullman, J. Gallagher, A. Gamkrelidze, G. Guo, J. He, M. Helak, N. J. Henry, E. N. Hulland, B. M. Huntley, M. Kereselidze, A. Lazzar-Atwood, K. E. LeGrand, A. Lindstrom, E. Linebarger, P. A. Lotufo, R. Lozano, B. Magistro, D. C. Malta, J. Månsson, A. M. Mantilla Herrera, F. Marinho, A. H. Mirkuzie, A. T. Misganaw, L. Monasta, P. Naik, S. Nomura, E. G. O'Brien, J. K. O'Halloran, L. T. Olana, S. M. Ostroff, L. Penberthy, R. C. Reiner Jr., G. Reinke, A. L. P. Ribeiro, D. F. Santomauro, M. I. Schmidt, D. H. Shaw, B. S. Sheena, A. Sholokhov, N. Skhvitaridze, R. J. D. Sorensen, E. E. Spurlock, R. Syailendrawati, R. Topor-Madry, C. E. Troeger, R. Walcott, A. Walker, C. S. Wiysonge, N. A. Worku, B. Zigler, D. M. Pigott, M. Naghavi, A. H. Mokdad, S. S. Lim, S. I. Hay, E. Gakidou, C. J. L. Murray, COVID-19 Excess Mortality Collaborators, Estimating excess mortality due to the COVID-19 pandemic: A systematic analysis of COVID-19-related mortality, 2020-21. *Lancet* **399**, 1513–1536 (2022). doi:10.1016/S0140-6736(21)02796-3 Medline

64. F. Wu, S. Zhao, B. Yu, Y.-M. Chen, W. Wang, Z.-G. Song, Y. Hu, Z.-W. Tao, J.-H. Tian, Y.-Y. Pei, M.-L. Yuan, Y.-L. Zhang, F.-H. Dai, Y. Liu, Q.-M. Wang, J.-J. Zheng, L. Xu, E. C. Holmes, Y.-Z. Zhang, A new coronavirus associated with human respiratory disease in China. *Nature* **579**, 265–269 (2020). doi:10.1038/s41586-020-2008-3 Medline

65. I. Aksamentov, C. Roemer, E. Hodcroft, R. Neher, Nextclade: Clade assignment, mutation calling and quality control for viral genomes. *J. Open Source Softw.* **6**, 3773 (2021). doi:10.21105/joss.03773

66. D. P. Martin, A. Varsani, P. Roumagnac, G. Botha, S. Maslamoney, T. Schwab, Z. Kelz, V. Kumar, B. Murrell, RDP5: A computer program for analyzing recombination in, and removing signals of recombination from, nucleotide sequence datasets. *Virus Evol.* **7**, veaa087 (2020). doi:10.1093/ve/veaa087 Medline

67. M. N. Price, P. S. Dehal, A. P. Arkin, FastTree 2—Approximately maximum-likelihood trees for large alignments. *PLOS ONE* **5**, e9490 (2010). doi:10.1371/journal.pone.0009490 Medline

68. J. Felsenstein, Confidence limits on phylogenies: An approach using the bootstrap. *Evolution* **39**, 783–791 (1985). doi:10.1111/j.1558-5646.1985.tb00420.x Medline

69. A. Rambaut, T. T. Lam, L. Max Carvalho, O. G. Pybus, Exploring the temporal structure of heterochronous sequences using TempEst (formerly Path-O-Gen). *Virus Evol.* **2**, vew007 (2016). doi:10.1093/ve/vew007 Medline

70. A.-A. Popescu, K. T. Huber, E. Paradis, ape 3.0: New tools for distance-based phylogenetics and evolutionary analysis in R. *Bioinformatics* **28**, 1536–1537 (2012). doi:10.1093/bioinformatics/bts184 Medline

71. P. Sagulenko, V. Puller, R. A. Neher, TreeTime: Maximum-likelihood phylodynamic analysis. *Virus Evol.* **4**, vex042 (2018). doi:10.1093/ve/vex042 Medline

72. S. Wang, X. Xu, C. Wei, S. Li, J. Zhao, Y. Zheng, X. Liu, X. Zeng, W. Yuan, S. Peng, Molecular evolutionary characteristics of SARS-CoV-2 emerging in the United States. *J. Med. Virol.* **94**, 310–317 (2022). doi:10.1002/jmv.27331 Medline

73. G. Yu, Using ggtree to visualize data on tree-like structures. *Curr. Protoc. Bioinformatics* **69**, e96 (2020). doi:10.1002/cpbi.96 Medline

74. H. Wickham, ggplot2. *Wiley Interdiscip. Rev. Comput. Stat.* **3**, 180–185 (2011). doi:10.1002/wics.147

75. J. Hadfield, C. Megill, S. M. Bell, J. Huddleston, B. Potter, C. Callender, P. Sagulenko, T. Bedford, R. A. Neher, Nextstrain: Real-time tracking of pathogen evolution. *Bioinformatics* **34**, 4121–4123 (2018). doi:10.1093/bioinformatics/bty407 Medline

76. S. E. James, CERI-KRISP/SARS-CoV-2-epidemic-in-Africa: Expanding Africa SARS-CoV-2 sequencing capacity in a fast evolving pandemic analysis. Zenodo (2022); https://doi.org/10.5281/zenodo.7006806.

77. T. Ward, A. Johnsen, Understanding an evolving pandemic: An analysis of the clinical time delay distributions of COVID-19 in the United Kingdom. *PLOS ONE* **16**, e0257978 (2021). doi:10.1371/journal.pone.0257978 Medline
