# Supplementary material for: The evolving SARS-CoV-2 epidemic in Africa: Insights from rapidly expanding genomic surveillance
Source: Science. 2022 Sep 15:eabq5358. doi: 10.1126/science.abq5358 (PMC9529057; doi:10.1126/science.abq5358)
Supplement: Supplementary file 2 — MDAR Reproducibility Checklist [file science.abq5358_mdar_reproducibility_checklist.pdf]

Materials Design Analysis Reporting (MDAR)
Checklist for Authors

The MDAR framework establishes a minimum set of requirements in transparent reporting applicable to studies in the life sciences (see Statement of Task: doi:10.31222/osf.io/9sm4x.). The MDAR checklist is a tool for authors, editors, and others seeking to adopt the MDAR framework for transparent reporting in manuscripts and other outputs. Please refer to the MDAR Elaboration Document for additional context for the MDAR framework.

For all that apply, please note where in the manuscript the required information is provided.

Materials:

Newly created materials	indicate where provided: page no/section/legend)	n/a
The manuscript includes a dedicated "materials availability statement" providing transparent disclosure about availability of newly created materials including details on how materials can be accessed and describing any restrictions on access.	Not applicable.	X

Antibodies	indicate where provided: page no/section/legend)	n/a
For commercial reagents, provide supplier name, catalogue number and RRID, if available.	Not applicable.	X

DNA and RNA sequences	indicate where provided: page no/section/legend)	n/a
**Short novel DNA or RNA including primers, probes:** Sequences should be included or deposited in a public repository.	All sequences used in the study are publicly available from GISAID.	X

Cell materials	indicate where provided: page no/section/legend	n/a
**Cell lines:** Provide species information, strain. Provide accession number in repository **OR** supplier name, catalog number, clone number, **OR** RRID.	Not applicable.	X
**Primary cultures:** Provide species, strain, sex of origin, genetic modification status.	Not applicable.	X

Experimental animals	indicate where provided: page no/section/legend)	n/a
**Laboratory animals or Model organisms:** Provide species, strain, sex, age, genetic modification status. Provide accession number in repository **OR** supplier name, catalog number, clone number, **OR** RRID.	Not applicable.	X
**Animal observed in or captured from the field:** Provide species, sex, and age where possible.	Not applicable.	X

Plants and microbes	indicate where provided: page no/section/legend)	n/a
**Plants:** provide species and strain, ecotype and cultivar where relevant, unique accession number if available, and source (including location for collected wild specimens).	Not applicable.	X
**Microbes:** provide species and strain, unique accession number if available, and source.	Not applicable.	X

Human research participants	indicate where provided: page no/section/legend) or state if these demographics were not collected	n/a
If collected and within the bounds of privacy constraints report on age, sex and gender or ethnicity for all study participants.	All age, sex, gender and ethnicity details are captured on GISAID.	X

Design:

Study protocol	indicate where provided: page no/section/legend)	n/a
If study protocol has been pre-registered, provide DOI. For clinical trials, provide the trial registration number **OR** cite DOI.	Not applicable.	X

Laboratory protocol	indicate where provided: page no/section/legend)	n/a
Provide DOI **OR** other citation details if detailed step-by-step protocols are available.	Not applicable.	X

Experimental study design (statistics details)		
**For in vivo studies:** State whether and how the following have been done	**indicate where provided: page no/section/legend. If it could have been done, but was not, write not done**	**n/a**
Sample size determination	100 000 African SARS-CoV-2 genomes	
Randomisation	Not applicable.	X
Blinding	Not applicable.	X
Inclusion/exclusion criteria	Inclusion and exclusion criteria explained in the methods section. See page 25 paragraph final paragraph.	

Sample definition and in-laboratory replication	indicate where provided: page no/section/legend	n/a
State number of times the experiment was replicated in laboratory.	Not applicable.	X
Define whether data describe technical or biological replicates.	Not applicable.	X

Ethics	indicate where provided: page no/section/legend	n/a
**Studies involving human participants:** State details of authority granting ethics approval (IRB or equivalent committee(s), provide reference number for approval.	This project relied on sequence data and associated metadata publicly shared by the GISAID data repository and adhere to the terms and conditions laid out by GISAID. The African samples processed in this study were obtained anonymously from material exceeding the routine diagnosis of SARS-CoV-2 in African public and private health laboratories. Individual institutional review board (IRB) references or material transfer agreements (MTAs) for countries are listed below.<br><br>Angola - (MTA - CON8260), Botswana - Genomic surveillance in Botswana was approved by the Health Research and Development Committee (Protocol HPDME 13/18/1), Egypt - Surveillance in Egypt was approved by the Research Ethics Committee of the National Research Centre (Egypt) (protocol number 14 155, dated March 22, 2020), Kenya - samples were collected under the Ministry of Health protocols as part of the national COVID-19 public health response. The whole genome sequencing study protocol was reviewed and approved by the Scientific and Ethics Review Committee (SERU) at Kenya Medical Research Institute (KEMRI), Nairobi, Kenya (SERU protocol #4035), Nigeria – (NHREC/01/01/2007), Mali - study of the sequence of SARS-CoV-2 isolates in Mali - Letter of Ethical Committee (N0-2020 /201/CE/FMPOS/FAPH of 09/17/2020), Mozambique - (MTA - CON7800), Malawi - (MTA - CON8265), South Africa - The use of South	

	African samples for sequencing and genomic surveillance were approved by University of KwaZulu-Natal Biomedical Research Ethics Committee (ref. BREC/00001510/2020); the University of the Witwatersrand Human Research Ethics Committee (HREC) (ref. M180832); Stellenbosch University HREC (ref. N20/04/008_COVID-19); the University of the Free State Research Ethics Committee (ref. UFS-HSD2020/1860/2710) and the University of Cape Town HREC (ref. 383/2020), Tunisia - for sequences derived from sampling in Tunisia, all patients provided their informed consent to use their samples for sequencing of the viral genomes. The ethical agreement was provided to the research project ADAGE (PRFCOVID19GP2) by the Committee of protection of persons (Tunisian Ministry of Health) under the reference (CPP SUD N 0265/2020), Uganda - The use of samples and sequences from Uganda were approved by the Uganda Virus Research Institute - Research and Ethics Committee UVRI-REC Federalwide Assurance [FWA] FWA No. 00001354, study reference - GC/127/20/04/771 and by the Uganda National Council for Science and Technology, reference number - HS936ES) and Zimbabwe (MTA - CON8271).<br><br>See page 24 under "Ethics statement".	
**Studies involving experimental animals:** State details of authority granting ethics approval (IRB or equivalent committee(s), provide reference number for approval.	Not applicable.	X
**Studies involving specimen and field samples:** State if relevant permits obtained, provide details of authority approving study; if none were required, explain why.	Not applicable.	X

Dual Use Research of Concern (DURC)	indicate where provided: page no/section/legend	n/a
If study is subject to dual use research of concern regulations, state the authority granting approval and reference number for the regulatory approval.	Not applicable.	X

Analysis:

Attrition	indicate where provided: page no/section/legend	n/a
Describe whether exclusion criteria were preestablished. Report if sample or data points were omitted from analysis. If yes report if this was due to attrition or intentional exclusion and provide justification.	Exclusion criteria were preestablished and discussed in methods (see page 25). Briefly, all African sequences were passed through the NextClade analysis pipeline in order to identify and exclude: (*i*) sequences missing >10% of the SARS-CoV-2 genome, (*ii*) sequences that deviate by >70 nucleotides from the Wuhan reference strain, (*iii*) sequences with >10 ambiguous bases, (*iv*) clustered mutations, and (*v*) sequences flagged as problematic by NextClade. Additionally, Omicron variants were screened for traces of viral recombination with RDP5.23 using a P-value 0.05 or lower cut-off as evidence of recombination. A large number of sequences were removed (n=57 421) with incomplete sequences (<90% genome coverage) being the biggest contributor.	

Statistics	indicate where provided: page no/section/legend	n/a
Describe statistical tests used and justify choice of tests.	We used a negative binomial regression, with a log link function and maximum likelihood estimation of theta, to investigate the relationship between the number of SARS-CoV-2 genomes produced per week and the weekly number of reported COVID-19 cases in Africa. The number of genomes and reported cases per million were summed into corresponding weekly intervals. Both variables are count data and were skewed with high variance about the mean, with low frequency of zero values. A negative binomial regression was the most suitable model (rather than a Poisson regression as supported by likelihood-ratio test) to use with this dataset wherein the number of cases was used as a predictor of number of genomes (response variable).<br><br>Statistical tests were sited in figure legends.	

Data availability	indicate where provided: page no/section/legend	n/a
For newly created and reused datasets, the manuscript includes a data availability statement that provides details for access or notes restrictions on access.	Study does not contain new data.	X
If newly created datasets are publicly available, provide accession number in repository **OR** DOI **OR** URL and licensing details where available.	Study does not contain new data.	X
If reused data is publicly available provide accession number in repository **OR** DOI **OR** URL, **OR** citation.	Made available on GitHub (https://github.com/CERI-KRISP/SARS-CoV-2-epidemic-in-Africa). Repo contains all metadata files (containing GISAID sequence IDs), raw maximum likelihood trees, all time scaled and annotated tree topologies as well as all scripts and data to reproduce figures and statistical tests.<br><br>See Data Availability section on page 41.	

Code availability	indicate where provided: page no/section/legend	n/a
For all newly generated custom computer code/software/mathematical algorithm or re-used code essential for replicating the main findings of the study, the manuscript includes a data availability statement that provides details for access or notes restrictions.	Does not contain new code.	X

If newly generated code is publicly available, provide accession number in repository, **OR** DOI **OR** URL and licensing details where available. State any restrictions on code availability or accessibility.	Does not contain new code.	X
If reused code is publicly available provide accession number in repository **OR** DOI **OR** URL, **OR** citation.	Made available on GitHub (https://github.com/CERI-KRISP/SARS-CoV-2-epidemic-in-Africa).<br><br>See Data Availability section on page 41.	

Reporting

MDAR framework recommends adoption of discipline-specific guidelines, established and endorsed through community initiatives. Journals have their own policy about requiring specific guidelines and recommendations to complement MDAR.

Adherence to community standards	indicate where provided: page no/section/legend	n/a
State if relevant guidelines (e.g., ICMJE, MIBBI, ARRIVE) have been followed, and whether a checklist (e.g., CONSORT, PRISMA, ARRIVE) is provided with the manuscript.	Not applicable.	X
