# Supplementary material for: The evolving SARS-CoV-2 epidemic in Africa: Insights from rapidly expanding genomic surveillance
Source: Science. 2022 Sep 15:eabq5358. doi: 10.1126/science.abq5358 (PMC9529057; doi:10.1126/science.abq5358)
Supplement: Supplementary file 3 — Tables S3 and S4 [file science.abq5358_tables_s3_and_s4.zip › science.abq5358_captions_for_tables_s3_and_s4.pdf]

Supplementary Table S3 (excel file): Sequencing and epidemiological reporting survey results. S3.1) Aggregate results of the survey with responses from 25 countries across the continent. S3.2) frequency of epidemiological reporting (i.e. of new cases and deaths) in the different countries. S3.3) Sequencing strategies employed by different countries as the pandemic progressed. S3.4) The proportion of sequences from the major administrative regions of each country (i.e. provinces, districts or regions). The table can also be found at the github repository (https://github.com/CERI-KRISP/SARS-CoV-2-epidemic-in-Africa).

Supplementary Table S4 (excel file): GISAID acknowledgment table. The table can also be found at the github repository (https://github.com/CERI-KRISP/SARS-CoV-2-epidemic-in-Africa).
